# Multi-omics analysis reveals genomic, clinical and immunological features of SARS-CoV-2 virus target genes in pan-cancer

**DOI:** 10.3389/fimmu.2023.1112704

**Published:** 2023-02-17

**Authors:** Yong Liao, Jiaojiao Wang, Jiami Zou, Yong Liu, Zhiping Liu, Zunnan Huang

**Affiliations:** ^1^ Key Laboratory of Computer-Aided Drug Design of Dongguan City, The First Dongguan Affiliated Hospital, Guangdong Medical University, Dongguan, China; ^2^ Department of Pharmacy, Maoming People’s Hospital, Maoming, China; ^3^ Key Laboratory of Big Data Mining and Precision Drug Design of Guangdong Medical University, Key Laboratory for Research and Development of Natural Drugs of Guangdong Province, School of Pharmacy, Guangdong Medical University, Dongguan, China; ^4^ Center of Scientific Research, Department of Cardiology, Maoming People’s Hospital, Maoming, China; ^5^ Guangdong Province Key Laboratory of Pharmacodynamic Constituents of TCM and New Drugs Research, College of Pharmacy, Jinan University, Guangzhou, China

**Keywords:** SARS-CoV-2, pan-cancer, multi-omics, target genes, immunological features

## Abstract

The SARS-CoV-2 virus, also known as the severe acute respiratory syndrome coronavirus 2, has raised great threats to humans. The connection between the SARS-CoV-2 virus and cancer is currently unclear. In this study, we thus evaluated the multi-omics data from the Cancer Genome Atlas (TCGA) database utilizing genomic and transcriptomic techniques to fully identify the SARS-CoV-2 target genes (STGs) in tumor samples from 33 types of cancers. The expression of STGs was substantially linked with the immune infiltration and may be used to predict survival in cancer patients. STGs were also substantially associated with immunological infiltration, immune cells, and associated immune pathways. At the molecular level, the genomic changes of STGs were frequently related with carcinogenesis and patient survival. In addition, pathway analysis revealed that STGs were involved in the control of signaling pathways associated with cancer. The prognostic features and nomogram of clinical factors of STGs in cancers have been developed. Lastly, by mining the cancer drug sensitivity genomics database, a list of potential STG-targeting medicines was compiled. Collectively, this work demonstrated comprehensively the genomic alterations and clinical characteristics of STGs, which may offer new clues to explore the mechanisms on a molecular level between SARS-CoV-2 virus and cancers as well as provide new clinical guidance for cancer patients who are threatened by the COVID-19 epidemic.

## 1 Introduction

Cancer represents a leading cause of death and a serious obstacle to the global improvement of life quality. In 2020, 19.3 million new cancer cases and approximately 10 million cancer-related deaths are expected to occur globally, based on the statistics from GLOBOCAN (1). The burden of cancer incidence and mortality is still speedily growing all over the world. Seeking new treatment options is extremely urgent for cancer patients.

The COVID-19 pandemic caused by the severe acute respiratory syndrome coronavirus 2 (SARS-CoV-2), is currently having a profound influence on global health (2). The influence of COVID-19 has been unprecedented thus far, and long-term symptoms could have unexpectedly devastating effects (3, 4). Increasing evidence indicates that a variety of symptoms can persevere after the acute infection has been cleared in many COVID-19 patients. As a result, symptoms may have appeared as a result of the altered immune microenvironment caused by COVID-19. Given the long existence of the symptoms and the huge population infected by SARS-CoV-2 virus, the influence of COVID-19 on cancer, which is also tightly tied to the immune system, needs extra caution. Furthermore, viruses and cancers have significant correlations, which have been proven by numerous studies. More than 15% of malignancies are directly caused by viruses (5). Specifically, multiple human oncogenic viruses, including human hepatitis B (HBV) and C (HCV) viruses, human papillomavirus (HPV), Epstein-Barr virus (EBV), and Kaposi’s sarcoma-associated herpesvirus (KSHV), have been identified after an exhaustive search for viruses related to human cancers (6–9). SARS-CoV-2 has recently been discovered to prevent the growth of Hodgkin’s lymphoma (10). The human immune system can be strengthened by SARS-CoV-2 RNA vaccines to eradicate cancer (11). The connection between malignancies and SARS-CoV-2, however, has not been fully investigated. Viruses can affect tumor growth through specific target genes, hence, the involvement of SARS-CoV-2 target genes in cancers is worth investigating (12).

**Figure 1:**
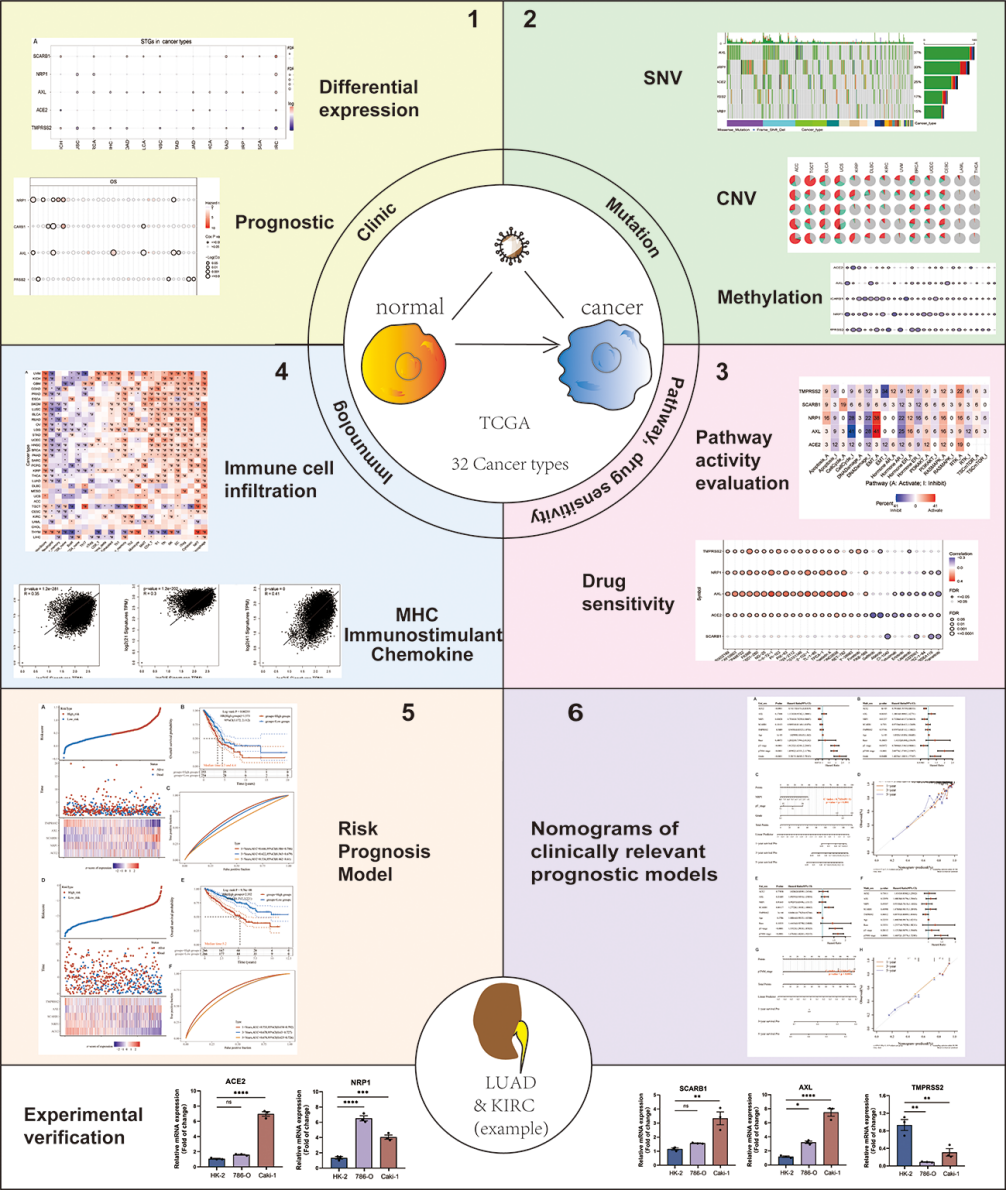
Flow chart of the study. Note: #: FDR ≤ 0.05; *: *p* ≤ 0.05; **: *p* ≤ 0.01; ***: *p* ≤ 0.005; ****: *p* ≤ 0.001; ns, not significant. *p* which represents the statistically significant need to be always italicized and we also unified it as a lowercase character.

This study thoroughly evaluated the genomic mutation, clinical characteristics, methylation, the activation of signature-related pathways and immunological characteristics of SARS-CoV-2 target Genes (STGs) in thirty-three solid tumors. At the same time, taking lung adenocarcinoma (LUAD) and kidney renal clear cell carcinoma (KIRC) as examples, risk prognostic models based on STGs were constructed, and nomogram prognostic models were constructed by further integrating clinical features (**Figure 1**). This study examines the close correlation between these features and clinical survival in many types of malignancy. This will further reveal the important role of STGs in tumors. Therefore, targeting STGs may be a promising strategy for treating patients with cancer, as well as a potential resource for research on the relationship between coronaviruses and tumors, and may further provide potential ideas for viral therapies that target tumors. At the same time, with the prevalence of COVID-19, this study will be more helpful for us to understand the specific response of tumor patients to SARS-CoV-2 virus.

## 2 Materials and methods

### 2.1 Dataset download and processing

The STGs were retrieved from the VThunter database (https://db.cngb.org/VThunter) (13) and literature. Clinical parameters (n = 11,160) as well as gene expression (n = 10,471), Single-nucleotide variations (SNV) (n = 10,234), copy number variation (CNV) (n = 11,461), and methylation (n = 10,063) were obtained and processed from GSCA (Gene Set Cancer Analysis, http://bioinfo.life.hust.edu.cn/GSCA/#/), which collected the pan-cancer data of the Cancer Genome Atlas (TCGA) database (https://portal.gdc.cancer.gov/) (14–16). Reverse phase protein array (RPPA) data from The Cancer Proteome Atlas (TCPA) (https://tcpaportal.org/tcpa/index.html) was used to investigate pathways (17). The analysis between the gene expression and drug sensitivity was based on the Genomics of Drug Sensitivity in Cancer (GDSC) database (www.cancerrxgene.org) (18). The deadline for database website access and data download is September 15, 2022. Thirty-three cancer types with the number of samples included in this study (**Table 1**).

**Table 1:** Abbreviation of cancer types and the number of samples.

Cancer type	Abbreviation	n
Adrenocortical carcinoma	ACC	92
Breast cancer	BRCA	1,218
Bladder uroepithelial carcinoma	BLCA	411
Cervical squamous cell carcinoma and endocervical adenocarcinoma	CESC	310
Cholangiocarcinoma	CHOL	45
Colon adenocarcinoma	COAD	329
Lymphoid neoplasm diffuse large B-cell lymphoma	DLBC	48
Head and neck squamous cell carcinoma	HNSC	566
Esophageal carcinoma	ESCA	196
Glioblastoma multiforme	GBM	174
Kidney chromophobe	KICH	91
Kidney renal clear cell carcinoma	KIRC	606
Kidney renal papillary cell carcinoma	KIRP	323
Acute myeloid leukemia	LAML	173
Brain lower grade glioma	LGG	534
Liver hepatocellular carcinoma	LIHC	359
Lung adenocarcinoma	LUAD	576
Lung squamous cell carcinoma	LUSC	554
Mesothelioma	MESO	87
Ovarian serous cystadenocarcinoma	OV	309
Pancreatic adenocarcinoma;	PAAD	183
Pheochromocytoma and paraganglioma	PCPG	187
Prostate adenocarcinoma	PRAD	550
Rectal adenocarcinoma	READ	105
Sarcoma	SARC	265
Skin cutaneous melanoma	SKCM	474
Stomach adenocarcinoma	STAD	450
Testicular germ cell tumor	TGCT	156
Thyroid cancer	THCA	572
Thymoma	THYM	122
Uterine corpus endometrial carcinoma	UCEC	201
Uterine carcinosarcoma	UCS	57
Uveal melanoma	UVM	80

### 2.2 Differential gene expression and prognostic analysis

In the analysis of mRNA expression levels, only 14 cancer types (COAD, ESCA, KIRC, HNSC, PRAD, BRCA, BLCA, THCA, STAD, KIRP, LUAD, LIHC, and KICH) were included because they contained more than 10 pairs of tumor and normal samples. The values of mRNA expressions from TCGA database were normalized by RNA-Seq by Expectation-Maximization (RSEM) values. The mean (tumor)/mean (normal) was used to calculate the fold change. *P*-values were calculated by t-test and adjusted by the false discovery rate (FDR). The expression of STGs in 33 malignancies as well as the related clinical survival data were integrated to stratify tumor samples into low and high expression groups for analysis of survival (19).

### 2.3 Single-nucleotide variant analysis

The TCGA database was accessed for SNV data. Seven mutation types which referred as disadvantageous mutations were included in this study: missense-mutation, nonsense-mutation, frame-shift-insertion, in-frame-insertion, frame-shift-deletion and splice-site.

### 2.4 Copy number variation analysis

Raw CNV data were obtained from the TCGA database and processed with GISTICS 2.0 to detect highly amplified or deleted areas (20). The level of mRNA expressions and CNV were combined and quantified by using the Spearman correlation analysis to determine the relationships (21). FDR was used to adjust *p*-values. The Log-rank test was applied to determine the survival rates of different groups.

### 2.5 Methylation analysis

Methylation analysis was performed on the selected 14 cancer types which the paired data was available. T-test was utilized to evaluate the changes in methylation levels between tumor and normal groups. Relationships between STGs’ mRNA expression and their methylation levels were determined by using Spearman analysis. FDR was used to adjust p-values. Median methylation values were employed to stratify tumor samples into hypermethylated and hypomethylated groups for further survival analysis.

### 2.6 Related pathway analysis

Based on 10 pathways closely related with cancers, the pathway activity scores in thirty-three tumor samples were calculated, respectively. The pathways were listed as below: TSC/mTOR, RTK, RAS/MAPK, PI3K/AKT, hormone estrogen receptor (ER), hormone androgen receptor (AR), epithelial–mesenchymal transition (EMT), DNA damage response, cell cycle, and apoptotic pathway. STGs were grouped into low and high groups based on their expression levels between different pathway activity status (activation or repression), which were defined by the median pathway scores (22). The distinction in pathway activity score (PAS) across groups was determined by t-test. *P*-values were adjusted by FDR. We anticipated that gene “A” was a particular activator when PAS of Gene “A” with high expression was greater than PAS of Gene “A” with low expression. Likewise, when PAS (high expression of Gene “A”) was lower than PAS (low expression of Gene “A”), we reasoned that gene “A” was a repressor (23).

### 2.7 Drug sensitivity analysis

The IC50 of 265 small molecular compounds in 860 cell lines and STGs expression under the drug treatment were retrieved from the GDSC database. We performed Pearson correlation analysis to establish the association between STGs mRNA expression and the IC50 concentration of the drugs. FDR was utilized to adjust *p*-values. A positive correlation suggested that upregulated gene expression was involved in the development of drug resistance.

### 2.8 Analysis of the relationship between STGs and immunity

Correlation coefficients were calculated by using the ImmuCellAI algorithm to evaluate the infiltration of twenty-four types of immune cells (24). Spearman correlation was utilized to analyze the link between immune cell infiltration and GSVA score of STGs, with *P*-values adjusted by FDR. Markers of the three immune-related pathways: chemokines, MHC pathway, and immunostimulants were obtained from the TISIDB database (http://cis.hku.hk/TISIDB/) (25). The GEPIA2 database was further used to investigate the link (Pearson coefficient) between STGs expression and the above three immune-related pathways (26).

### 2.9 Establishment and validation of risk model

Multivariate Cox regression analysis was performed to create a risk model of prognosis by using the R software’s SURVIVAL package. The risk model was a RiskScore formula that included multiple genes with a weight. A negative score indicated that the certain gene was a risk factor, whereas a positive score indicated that it was a protective factor. Based on the median values of the calculated scores, patients were split into high-risk and low-risk groups. Log-rank test was applied to examine Kaplan-Meier survival. Additionally, ROC was utilized to evaluate the prediction accuracy of the model and analyze the interaction of genes within risk. The *p*-values and hazard ratios (HR) with 95 percent confidence intervals (CI) for Kaplan-Meier curves were calculated using the log-rank test and univariate Cox regression.

### 2.10 Nomogram analysis

The “forestplot” program was used for both univariate and multivariate cox regression analyses to create forest plots that show each variable’s *p*-value, HR as well as 95 percent CI. The “rms” software was used to generate nomograms on the basis of the multivariate Cox proportional risk analysis to forecast overall survival at one, three and five years, respectively. The nomograms graphically showed the outcomes of these variables and predicted each patient’s prognosis risk using the points assigned to each risk factor.

### 2.11 qRT-PCR analysis

Cell lines of HK-2, 786-O and Caki-1 were obtained from the American Type Culture Collection^®^ (ATCC, Virginia, US). In a 6-well plate, cells were seeded at a density of 50,000. When the cells had reached confluence, total RNA was extracted using Trizol reagent (Invitrogen, NY, US). cDNA was synthesized using an iScript cDNA synthesis kit (Bio Rad, Hercules, US). Using a qPCR equipment (LightCycler^®^ 480, Roche Life Sciences), real-time PCR was done with Power SYBR Green PCR Master Mix (Thermo Fisher Scientific, Massachusetts, US) and the gene-specific primers mentioned in **Table S1**. The β-actin served as an internal control. Using the efficiency-corrected 2^−△△^CT approach, the relative difference was reported as the fold matched control values.

## 3 Results

### 3.1 SARS-CoV-2 virus target gene identification

Four STGs including ACE2 (Angiotensin-converting enzyme 2), NRP1 (Neuropilin-1), SCARB1 (Scavenger Receptor Class B Member 1), and AXL were identified and retrieved from the VThunter database by querying the human receptors to which the SARS-CoV-2 virus targets (13). The developers of the database manually selected up-to-date datasets generated in animal scRNA-seq research, evaluated them using a single processing pipeline, categorized 107 viral receptors in 142 viruses, and derived correct expression signatures in 2100962 cells from 47 animal species (13).. Suggested by one of the reviewers, the additional STG TMPRSS2 (Transmembrane serine protease 2) that is also involved in the viral entry and spread of coronaviruses like ACE2, was further added for analysis. Information on these STGs and the relevant experimental supporting literature can be found in **Table 2**.

**Table 2:** STGs and the relevant experimental supporting literature.

Gene name	Gene symbol	Literature
Angiotensin I converting enzyme 2	ACE2	PMID: 32142651 (27)
Neuropilin 1	NRP1	PMID: 33082294 (28)
Scavenger receptor class B member 1	SCARB1	PMID: 33244168 (29)
AXL receptor tyrosine kinase	AXL	PMID: 33420426 (30)
Transmembrane serine protease 2	TMPRSS2	PMID: 32276929 (31) PMID: 34159616 (32)

### 3.2 Significant expression differences of STGs between tumor and normal tissues

**Figure 2:**
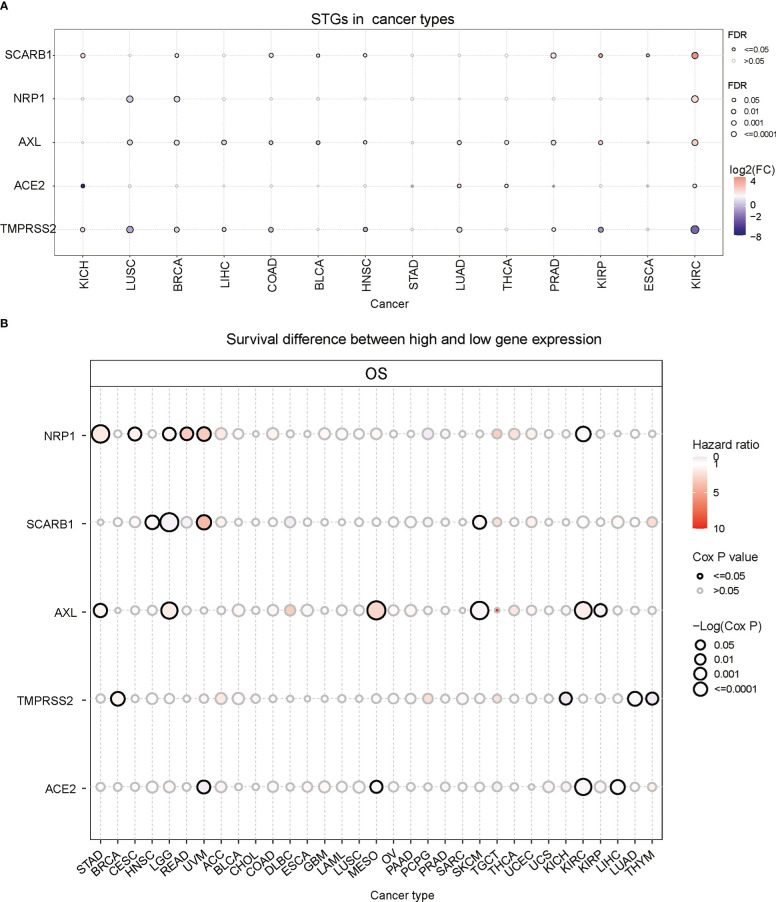
Gene expression and survival analysis of STGs. All genes with significant differential expression are displayed. **(A)** STGs expression differences between normal and tumor samples. **(B)** Survival analysis of STGs. The size of the dots represents the significance of the effect of the gene on survival for each cancer type; *p*-value was calculated based on Kaplan-Meier analysis. Red or blue dots showed that the expression of a certain gene was respectively associated with a poor or good survival of patients in the indicated cancer type.

STGs were aberrantly expressed in 13 solid tumors (*P*<0.05, **Figure 2A** and **Table S2**), including KICH, LUSC, BRCA, LIHC, BLCA, COAD, PRAD, THCA, LUAD, HNSC, ESCA, KIRP and KIRC, but not STAD. Particularly, SCARB1 expression levels were considerably elevated (*P*<0.05) in several malignancies. Except for TMPRSS2, which was down-regulated, the other four STGs were up-regulated in KIRC (*P*<0.05).

Then the correlation between gene expression and survival was further analyzed. The results showed that NRP1 expression was related to poor survival and regarded as a risk factor in STAD, CESC, READ and UVM (HR>1) but correlated with high survival as a protective factor in KIRC (HR<1) (**Figure 2B** and **Table S3**). SCARB1 was linked to poor survival (HR>1) in HNSC, UVM and SKCM but to high survival as a protective factor in LGG (HR<1). Additionally, AXL was a risk factor (HR>1) in STAD, LGG, MESO, and KIRC, but a protective factor (HR<1) in SKCM and KIRP. TMPRSS2 was a risk factor (HR>1) in BRCA, but a protective factor (HR<1) in KICH, LUAD and THYM. Finally, ACE2 was a protective factor (HR<1) in UVM, MESO, KIRC and LIHC. These findings implied that abnormal STG expression may influence the occurrence and prognosis of multiple types of cancers *via* different mechanisms.

### 3.3 Somatic mutations of STGs

**Figure 3:**
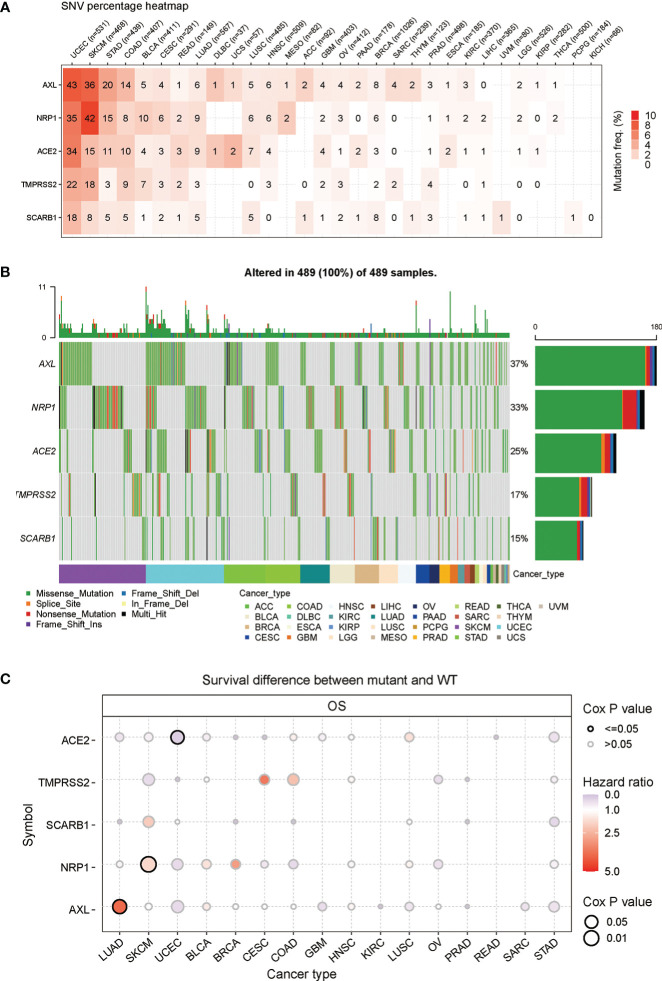
Frequencies of single nucleotide variants (SNVs) and variant types of STGs. **(A)** Mutation frequencies of STGs. Numbers represented the mutations harbored of the indicated gene in a particular cancer type. “0” indicated no mutations in the gene’s coding region; and “null” indicated no mutations in any region of the gene. **(B)** A waterfall plot of tumor distribution showed the distribution of mutations in STGs and the classification of SNV types. **(C)** The relationship between SNV and STGs survival. Risk ratios and Cox *P* values were presented by bubble color and size. The color of the bubbles, from blue to red, represented low to high hazard ratios; and bubble size was positively associated with the significance of Cox *P* value. Black border outlines indicated Cox *P* values ≤ 0.05.

Based on the previous analyses, the gene expression of STGs in different types of cancers and the relationship with survival have been showed. It suggested that STGs may be largely linked to multiple cancers. As we know, somatic mutations of certain genes are often considered the initiation of cancers. Hence, in this part, the single-nucleotide polymorphism (SNP) information of STGs was examined to determine the frequency and variation type presented in each cancer type. As shown in **Figure 3A** and **Table S4** mutations of STGs were existed in all the cancer types which were included in this study except KICH. Notably, STGs were more frequently mutated (up to 9%) in UCEC and SKCM compared to other cancers. Furthermore, the missense mutation was the main type of mutation. Based on the SNV percentage analysis, the missense mutation rates were ranked as follows: AXL (37%), NRP1 (33%), ACE2 (25%), TMPRSS2 (17%) and SARB1 (15%) (**Figure 3B**). Next, the analyses showed that the somatic mutations of ACE2 were protective factors in UCEC (HR<1), while the mutations of AXL in LUAD, NRP1 in SKCM were risk factors (HR>1). These findings implied that the mutations of STGs significantly affected the prognosis of multiple malignancies (**Figure 3C** and **Table S5**).

### 3.4 Copy number variation of STGs

**Figure 4:**
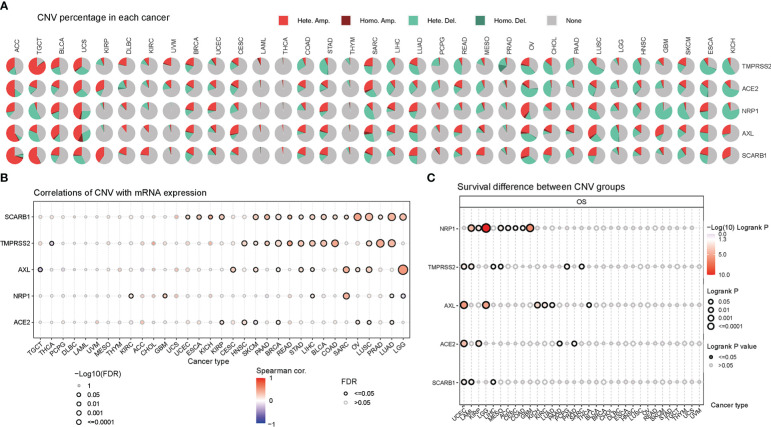
Copy number variation (CNV) was an influential factor in the abnormal expression of STGs. **(A)** Distribution of CNVs in thirty-three cancer types. Pie charts depicted the proportion of various CNV forms of a certain gene in a particular tumor, with different colors representing various CNV types. **(B)** Bubble plots indicated the correlations between the mRNA expression of STGs and CNV levels. Blue or red bubbles respectively represented the negative or positive correlation. The darker the bubble color, the stronger the association. The larger the bubble size, the greater the FDR significance. The black border outlines signified FDR ≤ 0.05. **(C)** Relationship between CNV and survival of STGs in cancer. The log-rank *P* values were represented by the size and color of the bubbles. The bubble size was positively connected with the significance of Log-rank *P* value. The bubble color from blue to red showed the relevance of Log-rank *P* value from low to high. The black border outlines indicated the Log-rank *P* value ≤ 0.05.

Except Single-nucleotide polymorphism, copy number variation also contributes largely to the genetic structural variation of the genomes. Thus, the CNV changes of STGs in cancers were further explored. Firstly, as seen in **Figure 4A** and **Table S6**, heterozygous amplification and deletion were the two primary CNV types of STGs (Typical CNV types included: TMPRSS2, ACE2, NRP1, AXL, SCARB1). The expression of STGs has significant correlation with CNV, such as SKCM, LUAD, LUSC, OV in SCARB1, LGG in AXL, and SARC in NRP1(**Figure 4B** and **Table S7**). In **Figure 4C** and **Table S8**, it is showed that CNV alterations of STGs were a risk factor in UCEC, LAML, LGG and GBM, which were inversely linked with survival. According to these findings, heterozygous amplification and deletion made up the majority of CNV alterations in STGs, which are correlated with STG expression and tumor prognosis, which may be indicative of a poor prognosis.

### 3.5 Methylation analysis of STGs

**Figure 5:**
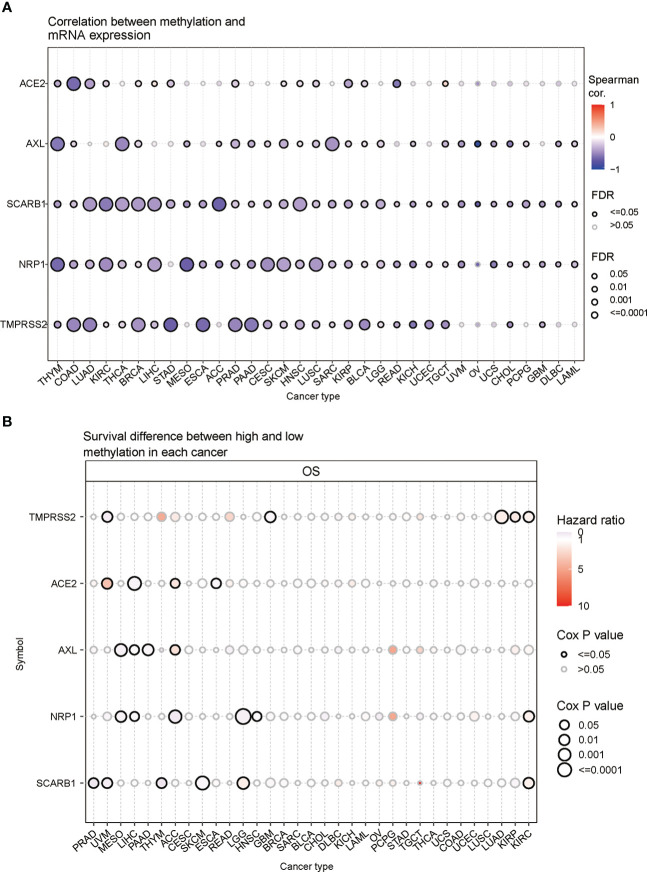
Methylation of STGs. **(A)** Correlation of methylation with STGs expression. Blue or red dots respectively represented negative or positive correlations The darker the color, the greater the association. **(B)** Disparities in survival between hypermethylated and hypomethylated STG samples. Risk ratios and Cox *P* values were indicated by the size and color of the bubbles. The color of the bubbles, from blue to red, showed low to high hazard ratios, and their size was positively connected with the significance of the Cox *P* value. Cox *P* values ≤ 0.05 were denoted by black border outlines.

In order to understand epigenetic regulation of STGs, the methylation status of STGs was investigated. The correlation studies revealed a negative relationship between the expression and methylation levels of STGs in the majority of malignancies (**Figure 5A** and **Table S9**). In **Figure 5B** and **Table S10**, hypermethylation of STGs was shown to play an important role in several tumors, as TMPRSS2 in UVM and GBM; ACE2 in LIHC and ESCA; AXL in MESO, LIHC and PAAD; NRP1 in MESO, LIHC, ACC, LGG and HNSC; and SCARB1 in PRAD, THYM, SKCM, UVM were considered as protective factors for good survival (HR<1), while TMPRSS2 in LUAD, KIRP and KIRC; ACE2 in ACC and UVM; AXL in ACC; NRP1 in KIRC; and SCARB1 in LGG and KIRC were regarded as risk factors for poor survival (HR>1).

### 3.6 Analysis of the relationship between STGs and cancer-related pathways

**Figure 6:**
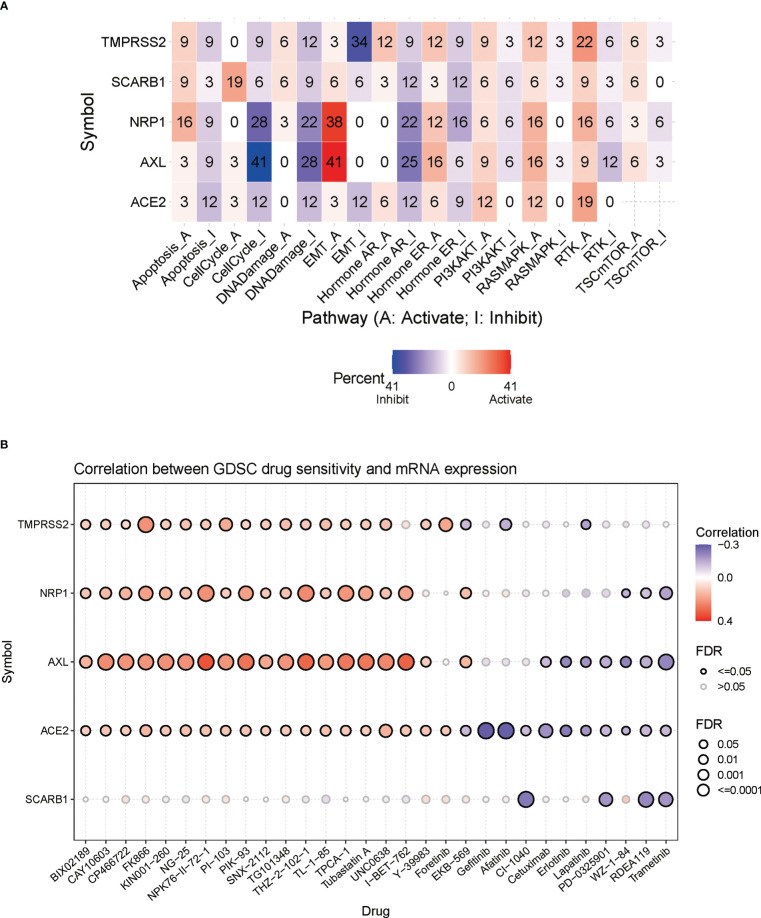
Correlation of STGs with cancer pathways and drug sensitivity. **(A)** Percentage of gene mutations of STGs’ potential impact on the pathway activity. The percentage indicated the mutations existed in a certain gene which had an effect on the pathways (FDR <= 0.05) over all the cancer types listed in this study. **(B)** Bubble plots summarized the correlation between STGs expression and drug IC50 (tumor drug resistance). Blue or red bubbles respectively represented negative or positive correlations. The deeper the color, the stronger the association. The larger the size, the greater FDR significance. Black border outlines denoted FDR ≤ 0.05.

In this part, the connections between STGs and pathways that are connected to cancer were explored. All five STGs were found to be heavily involved in a number of signaling pathways of cancer showing in **Figure 6A**, including apoptosis, cell cycle, DNA damage, EMT, hormone AR&ER, PI3K/AKT, RAS/MAPK, RTK, and TSC/mTOR. The number in each cell of the graph indicated a certain gene involved in the pathways of cancer types over all cancer types. In particular, AXL was deeply involved in the inhibition of cell cycle pathways but the activation of EMT pathways. These results indicated that STGs played an important role in the regulation of cancer-related pathways.

### 3.7 Analysis of STGs and tumor drug resistance

Genomic changes affect patient clinical response to chemotherapy and targeted therapies. In **Figure 6B**, it showed a significant relationship between STG expression and tumor resistance to 30 anti-tumor drugs (calculations by IC50). It suggested that aberrant STG expression may be a mediator of tumor resistance to chemotherapy and targeted therapy.

### 3.8 Relationship between STGs and immunity

**Figure 7:**
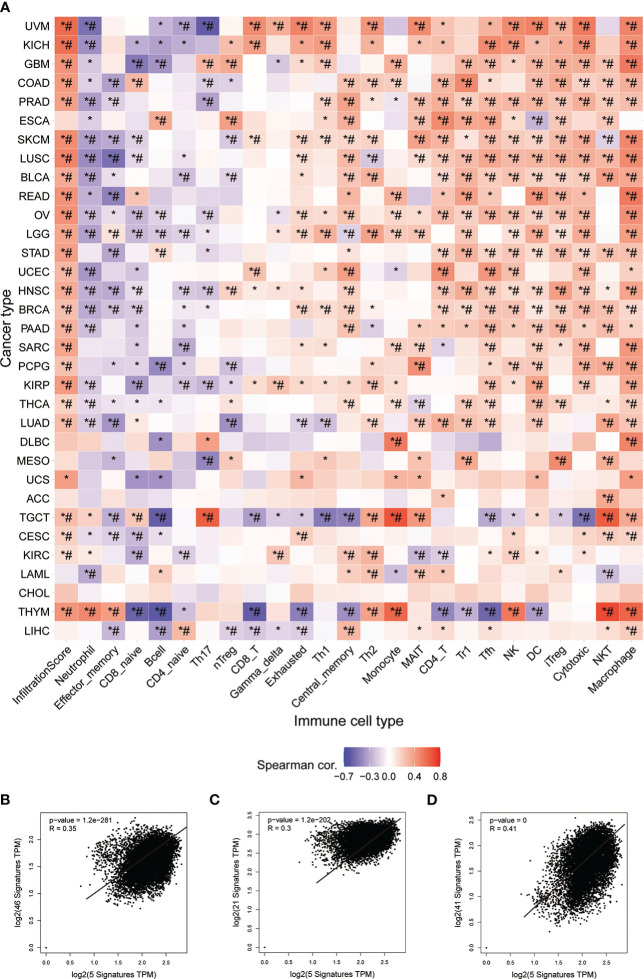
Immunoassay of STGs. **(A)** Association between immune cell infiltration and the Score of STGs. Heat map summarized the significance of *P* values and FDR based on the Pearman correlation analysis between input gene set GSVA scores and immune cell infiltration. Blue or red dots respectively indicated negative or positive correlations, where the darker the color, the stronger the correlation. *P value ≤ 0.05; ^#^FDR ≤ 0.05. **(B)** Correlation between STGs and immunostimulant immune pathway. R represented the correlation, and R>0 indicated the positive correlation. **(C)** Correlation of STGs with MHC immune pathway. R represented the correlation, R>0 indicated the positive correlation. **(D)** Correlation of STGs with chemokine immune pathway. R represented the correlation, and R>0 indicated the positive correlation.

InfiltrationScore in cancers and GSVA scores of STGs were associated with each other. In fact, the expression of the respective one-third or two-third immune infiltrating cells was negatively or positively correlated with GSVA scores of STGs in the majority of cancers (*: *P*-value ≤ 0.05; #: FDR ≤ 0.05) in **Figure 7A**. In particular, the expression of STGs was significantly and positively correlated with the immune infiltration fraction in most of the tumors except ESCA, DLBC, MESO, ACC, LAML, CHOL, LIHC. Our analyses also showed favorable correlations between STGs and immunostimulatory pathways (R = 0.35), MHC immune pathways (R = 0.3) as well as chemokine immune pathways (R = 0.41) (**Figures 7B-D**). According to these findings, STGs were linked to tumor immunity and may affect tumor growth by promoting tumor immune pathways.

### 3.9 Predictive characteristics of STGs in LUAD and KIRC

**Figure 8:**
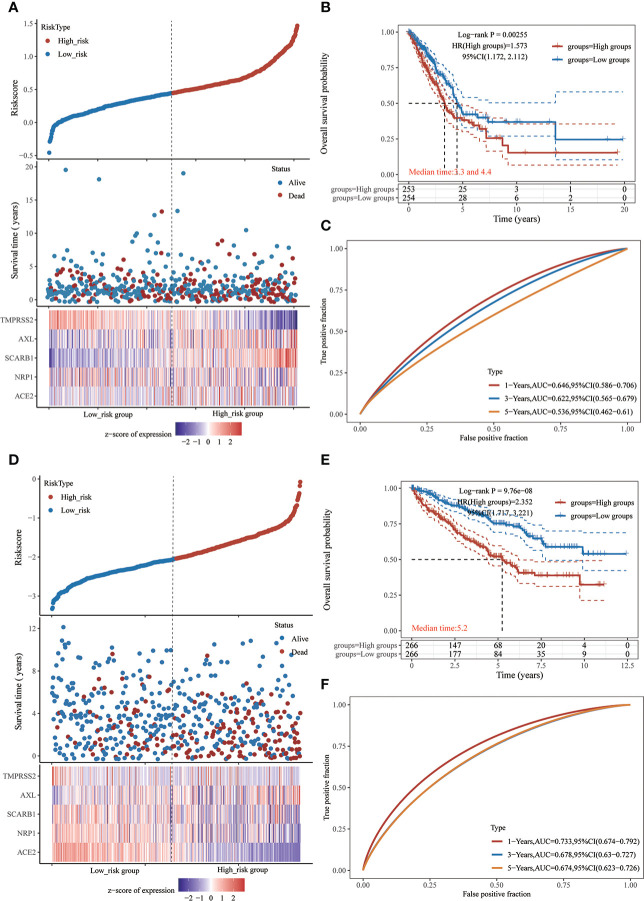
Prognostic risk model of STGs in LUAD and KIRC. **(A-C)** LUAD. **(D-F)** KIRC. **(A, D)** Sample distribution of risk score analysis according to the prognostic risk model of STGs. Different patterns of survival status and time for low- and high-risk clusters. Heat map of cluster analysis showed the expression of STGs of each patient based on the risk model. **(B, E)** Kaplan-Meier survival curves for patients OS in the low and high-risk groups. HR > 1 suggests a risk model while HR < 1 suggests a protection model; 95% CL denotes the HR confidence interval; Median survival time denotes the time on the basis of the survival rate at 50% in both low-risk and high-risk groups in years. **(C, F)** The ROC curves of the risk models at various times with larger AUC values indicating greater predictive power.

Because all five STGs are abnormally expressed in KIRC, this study considered KIRC for evaluating a risk prognostic model based on STGs. Meanwhile, as the SARS-CoV-2 is a respiratory virus, we also considered LUAD. Five STGs were identified by multivariate Cox regression analysis and were utilized to create predictive features in LUAD and KIRC. In LUAD, Riskscore = (0.135) *ACE2 + (0.001) * NRP1 + (0.1469) * SCARB1 + (0.1219) * AXL + (-0.1506) * TMPRSS2. In KIRC, riskscore = (-0.2469) * ACE2 + (-0.3409) * NRP1 + (-0.0526) * SCARB1 + (0.2364) * AXL + (-0.1833) * TMPRSS2. Based on the median cut-off point of the risk score, all patients were divided into high- and low-risk groups according to their prognosis scores. Each STGs expression value in the formula connected to the risk score was shown on the heat map. In both LUAD and KIRC, when the risk score rose, the survival decreased and the cancer-related mortality increased (**Figures 8A, D**). Patients with low risk scores were considered to have a greater chance of achieving the same survival time than those with high risk scores (**Figures 8B, E**). The AUC value of one-year survival analyzed by ROC for prognostic characteristics was 0.646 in LUAD, while the AUC value of one-year survival analyzed by ROC for prognostic characteristics was 0.733 in KIRC (**Figures 8C, F**). This result demonstrates the possibility of validity of STGs for tumor prediction and, more importantly, the important correlation between STGs and tumors. Interestingly, the prognostic value of LUAD is worse than that of KIRC, which may be due to severe LUAD and superimposition of diseases such as COVID-19.

### 3.10 Nomogram analysis of STGs and clinical factors in LUAD and KIRC

**Figure 9:**
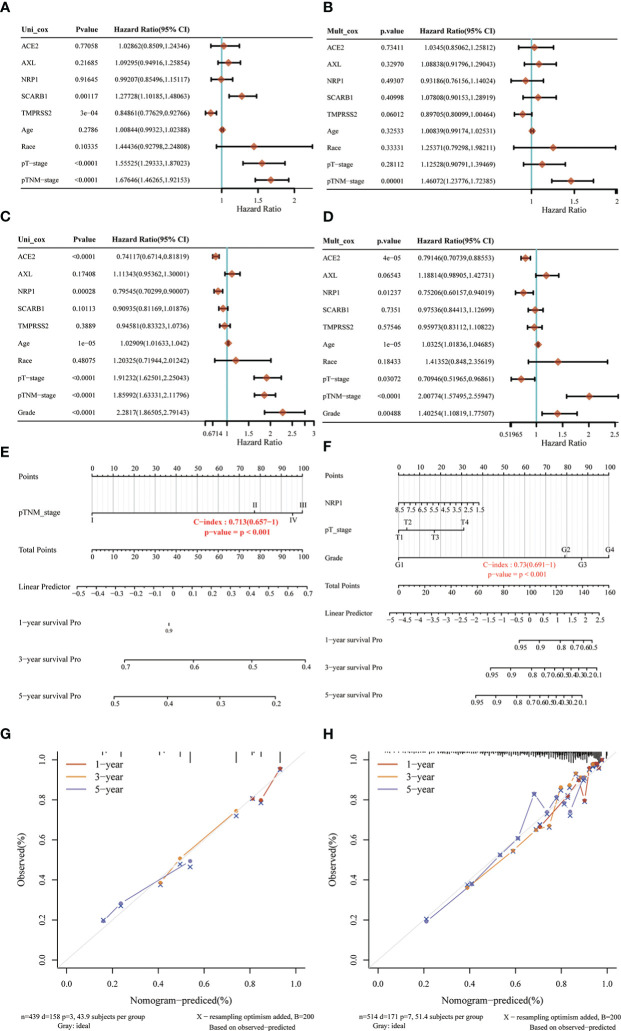
Nomogram analysis of STGs and clinical factors in LUAD and KIRC. **(A, B, E, G)** LUAD, **(C, D, F, H)** KIRC. **(A, C)** Forest map of univariate survival analysis. **(B, D)** Forest map of multivariate survival analysis, *p*-value<0.05 represents significant relation to OS. **(E, F)** Developed nomogram of KIRC patients. The nomogram was developed with the NRP1, pTstage and grade. **(G, H)** Calibration of nomograms. Calibration curves of the nomogram.

In order to develop a clinically applicable method to evaluate the chance of patient survival, it is needed to construct a prediction model that took clinicopathological parameters into consideration. Based on univariate and multivariate analyses of OS rates in LUAD (**Figures 9A, B**) and KIRC **(** **Figures 9C, D**), the nomogram line plots were built. They used Cox regression algorithm to predict 1-year, 3-year, and 5-year OS in the discovery group, and the predictors included NRP1, pT-stage, and grade. The C-index was found to be 0.713 in LUAD (**Figure 9E**) and 0.73 in KIRC (**Figure 9F**), which had predictive power. In comparison to ideal models in the whole cohort, calibration plots for the 1-year, 3-year, and 5-year OS rates in LUAD (**Figure 9G**) and in KIRC (**Figure 9H**) showed correct predictions.

### 3.11 qRT-PCR analysis of STGs in KIRC

**Figure 10:**
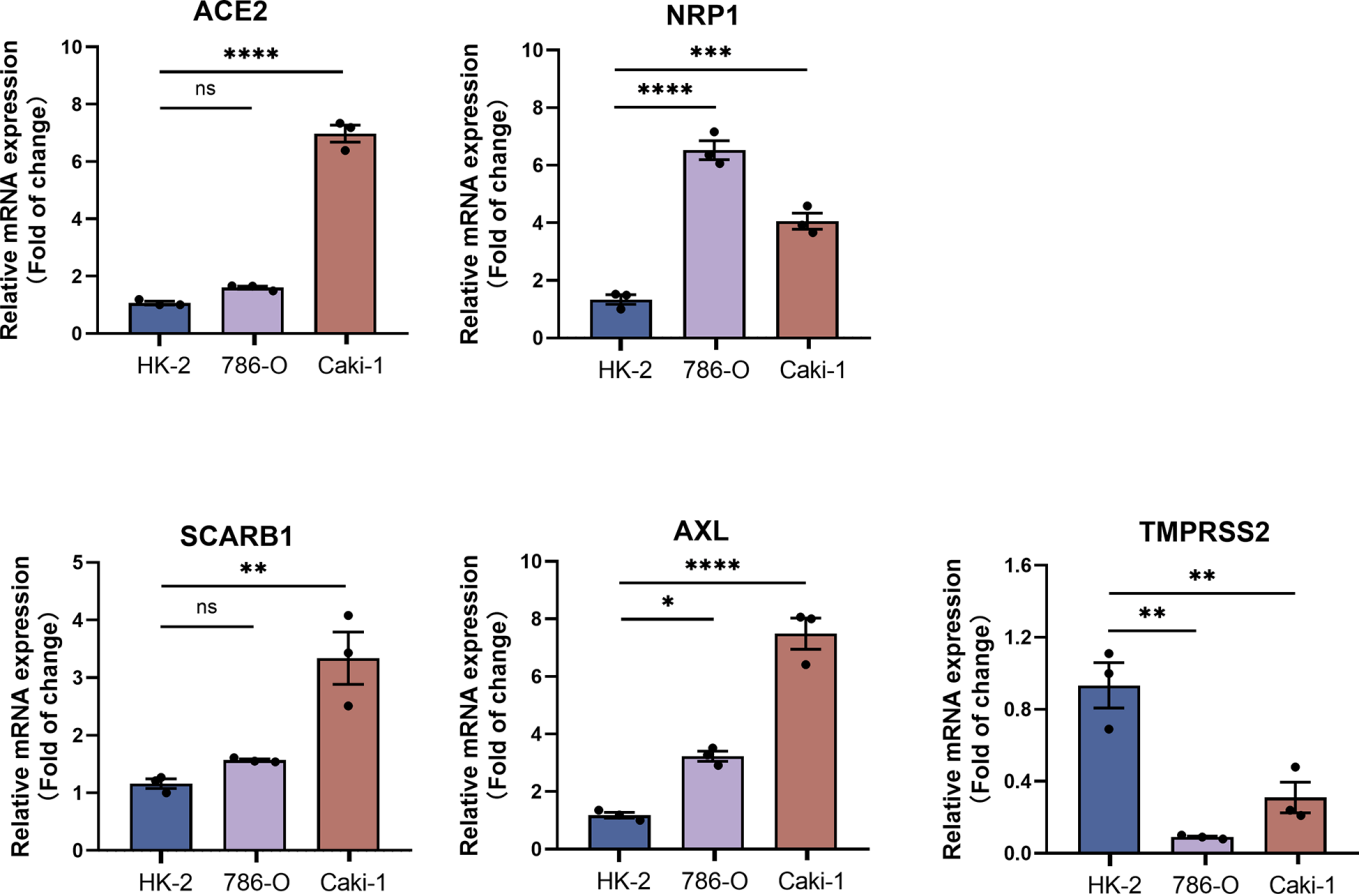
qRT-PCR analysis of STGs in KIRC (n = 3). *p*<0.05 was considered significant. *p<0.05, **p<0.01, ***p<0.001, ****p<0.001, ns, not significant.

The results showed that the transcript levels of all the STGs (ACE2, NRB1, SCARB1, AXL) were upregulated in KIRC. TMPRSS2 was downregulated in KIRC. Besides, the basal expression of TMPRSS2 is very low in the KIRC cell lines (**Figure 10**). This result experimentally validates that STGs are aberrantly expressed in tumors.

## 4 Discussion

Since it is largely unknown how SARS-CoV-2 virus and malignancies interact, more research is required. We extensively characterized SARS-CoV-2 virus target genes in multiple samples of thirty-three cancers by multi-omics data analysis. It has been shown that SARS-COV-2 infection can modulate the lung tumor microenvironment by disrupting the vulnerable immune mechanisms that lead to cytokine storms and cellular metabolic variation, resulting in increased severity (33). Potential immunosuppression, upregulated cytokine levels, changed expression of ACE-2 and TMPRSS2, and a prothrombotic state may exacerbate the effects of SARS-CoV-2 on cancer patients, have the potential to be exploited as biomarkers for serious diseases and therapeutic targets; and preliminary reports suggest that susceptibility to SARS-CoV-2 virus infection may be higher in cancer patients, but current evidence remains poor (34). Meanwhile, there is still a lack of research between neocoronavirus and cancer at the level of targeted genes. Our findings established a plausible link between SARS-CoV-2 virus target genes and tumors, as well as new information for cancer patients who are more vulnerable to virus infection.

**Figure 11:**
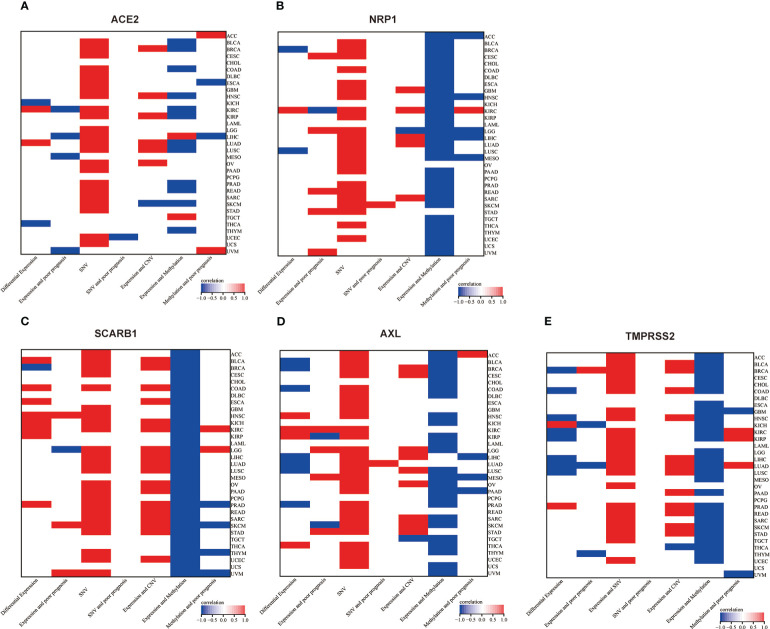
Two-dimensional map (heat map) of correlation of related factors based on genes. Red represents upward adjustment, positive correlation or presence. Blue represents down-regulation, negative correlation. White represents insignificant, non-existent or missing correlation. **(A)** ACE2, **(B)** NRP1, **(C)** SCARB1, **(D)** AXL, **(E)** TMPRSS2.

Firstly, we assessed the STG genetic signature in thirty-three malignancies. We discovered that five STGs expressed abnormally in a variety of malignancies and participated in carcinogenesis, which may have an impact on tumor prognosis. As shown in **Figure 11**, TMPRSS2 and AXL was down-regulated often in cancers but SCARB1 showed the reverse trends, while all of three was shown to be expressed abnormally more frequently than ACE2 and NRP1, which could not provide an expression patten possibly due to their limited situations. Interestingly, three STGs including NRP1, SCARB1, and AXL were demonstrated that their over-expressions were related with the bad prognosis more than the good prognosis of cancer patients, while the down-regulation of ACE2 and TMPRSS2 was exhibited to be more frequently associated with the poor prognosis. The results of the present study are in better agreement with the results of previous experimental studies. ACE2, a well-known host receptor of SARS-CoV-2 virus, could be a prognostic biomarker of BRCA, and its over-expression was associated with good prognosis (35, 36). TMPRSS2, a key gene mediating the entry of SARS-CoV-2 into humans, is highly expressed in prostate cancer and its high expression could promote the development of prostate cancer (37–40). NRP1, serving as a key factor of SARS-CoV-2 virus infection (41), could increase the susceptibility of cancer patients to SARS-CoV-2 virus and targeting (42), and its overexpression is associated with poor prognosis in bladder cancer cells and hepatocellular carcinoma, and inhibiting its expression could promote tumor cell apoptosis (43, 44). In addition, the increased expression of SCARB1 could promote cell transformation towards malignancy of clear cell renal cell carcinoma leading to poor prognosis of patients (45), while the over-expression of AXL could accelerate EGFR mutation and promote the metastasis, invasion or drug resistance of tumors such as lung cancer (46), colorectal cancer (47), pancreatic cancer (48) and prostate tumor (49).

Then, genetic and epigenetic studies have also shown that high frequencies of SNV, CNV, and methylation were noted in these five STGs, which may impact STGs expression in malignancies and affect the prognosis of cancer patients. **Figure 11** showed the SNVs of STGs occurred in most tumor types, however, they were not correlated with the tumor prognosis except for a few situations. In addition, the CNVs of the five STGs were all connected with their expressions in many tumors (SCARB1 most in 18 cancers, TMPRSS2 in 13 cancers, followed by AXL in 10 cancers, ACE2 in seven cancers and NRP1 in six cancers) and the majority of the associations showed positively, indicating that CNVs of STGs were normally associated with the up-regulation of STGs expression and were implicated in the promotion of tumor growth. Moreover, the methylations of STGs were also linked with their expressions in tumors. In fact, their associations (SCARB1 most in all investigated 33 cancers, followed by NRP1 in 31 cancers, TMPRSS2 in 27 cancers, AXL in 25 cancers and ACE2 in 13 cancers) appeared in more cancer types than those of CNVs with the same trends among the five STGs. However, the majority of the associations showed negatively, indicating aberrant hypermethylation of STGs promoted down-regulation of STGs expression in cancers and was involved in the control of tumor growth. Finally, though the association of methylations with the tumor prognosis were shown in no more than six cancer types, among which 5/6, 3/4, 2/3, 1/2, and 2/5 of them showed positively in NRP1, AXL, SCARB1 ACE2 and TMPRSS2 respectively, indicating a good outcome at least 40% of these STGs methylations in malignancies. On the basis of these findings, we hypothesized that genetic and epigenetic modifications in STGs would regulate the initiation and progression of multiple malignancies.

In this study, one point that can’t be ignored is that the role of immunity in tumor and COVID-19 is discussed through STGs. ACE2 and NRP1 play an important role in SARS-CoV-2 virus infection and tumor progression (50). The outcome of SARS-CoV-2 infection can be determined by the immune microenvironment of the tumor, and immune mechanisms in different tumors may have influenced the SARS-CoV-2 receptor expression among other factors leading to different evolutionary trajectories (51). Studying the potential immune impact between STGs and pan-cancer may be beneficial to further explore the different immune responses and mechanisms of SARS-CoV-2 infection in patients with different tumors and to improve immunotherapy for cancer. Recently, the human cell receptor ACE2 of SARS-CoV-2 has received extensive attention due to its role in SARS Co V-2 infection, and the up-regulation of ACE2 is related to the anti-tumor immune characteristics, the increase of PD-L1 expression and the good anti-PD-1/PD-L1/CTLA-4 immunotherapy response. Before being a common mediator of SARS-CoV-2 virus infection, NRP1 has been a regular visitor in cancer research, and it has been proved to be related to the immune system (28, 52). NRP1 is a specific surface marker of CD4 + 25regulatory T cells, maintaining the stability and function of regulatory T cells (53, 54). Because of its unique significance for cancer immunology and immunotherapy, it can be used as a potential target for immunotherapy (55, 56). AXL is also a research hotspot of tumor molecular targeted therapy, and Axl signal transduction promotes immunosuppression and tumor induction microenvironment by changing the secretion of cytokines that regulate the transport, migration, polarization and adhesion of immune cells (57–59). SCARB1 can be used as a receptor target to mediate drug regulation of immune microenvironment for the treatment of tumors (60, 61).

Each of the five STGs has proved its importance in the immune system and may affect the balance of the immune system. In this study, among 33 kinds of tumors, these five STGs are taken as a whole to study their relationship with immunology, it is found that among 33 kinds of tumors, they have significantly positive correlation with immune infiltration and also have significant correlation with various immune cells. At the same time, the STGs are positively correlated with MHC, immune system and chemokine. The results of this study indicate the immune importance of the five STGs in this study, and also reflect the possibility of SARS-CoV-2 virus infection profoundly affecting human immune system. Previous results and the results of this study show that these STGs play important roles in virus immunology and tumor immunology. The changes of these STGs may affect the immune system, and then affect virus infection and tumor occurrence and development. Both tumors and SARS-CoV-2 virus destroy the balance of the immune system through STGs, which may create an immune microenvironment that is easy for the other party to invade. The unbalanced immune microenvironment becomes a bridge between SARS-CoV-2 virus and tumors.

STGs were identified in the pathway investigation as significant cancer-related signaling pathway regulators. Different STGs cause erratic activation or inhibition and are connected to distinct signaling pathways which are relevant to cancer. These results raised the possibility that STGs work as a network of links among signaling pathways linked to cancer and may aid in the development of tumors. Furthermore, by providing potential directions for immunotherapy improvement, the data demonstrated that STGs play a crucial role in tumor immunotherapy. In the meanwhile, STGs were associated with tumor drug resistance based on the analyses we conducted. Therefore, targeting STGs for the treatment of cancer patients who are infected by SARS-CoV-2 virus might be an appropriate option.

In order to support the association between SARS-CoV-2 virus and cancers, this study examined the predictive properties of STGs in patients with malignancies and developed a prognostic risk model for KIRC based on STGs. Progression of cancers has been proven to be highly correlated with STGs which were used for risk modeling in this study. According to the study’s survival analysis, the established risk model was qualified to predict the survival of KIRC patients. It also developed a prognosis columnar map of KIRC that comprised clinical factors and STGs. In KIRC, NRP1 can not only build a risk prediction model with good prediction performance together with the other three STGs (**Figure 8**), but also show its key position in KIRC in univariate analysis and multivariate analysis, and can build a robust nomogram prognosis model with clinical factors (**Figure 9**). These two prediction models had strong predictive performance and further clarified the potential of NRP1 in STGs as a crucial gene for prognostic diagnosis choice. We discovered that these findings supported the prognostic and predictive significance of STGs in malignancies.

The results of this study are significant and offer fresh perspectives on the investigation of the mechanism underlying the interaction between malignancies and COVID-19. Secondly, STGs are altered at the most important regulatory levels, including the genetic and epigenetic levels, the milieu of immune infiltration, and the route level. These modifications may then result in variations in pharmacological effects, therapeutic response, and patient survival. The findings of this study also point to the genomic and clinical characteristics of STGs in tumors, revealing a close connection between SARS-CoV-2 virus and pan-cancer. COVID-19 may also be able to affect tumorigenesis and prognosis through STGs, and they are frequently immune-related, suggesting a potential resource with useful referential properties for viral and immunotherapy in tumors. The key drawbacks of this study are the paucity of studies to explore the influence of SARS-CoV-2 virus infection on tumors *via* STGs. Additional research is required to validate these findings.

## 5 Conclusion

In summary, the multi-omics analysis elucidated the genomic and clinical features of SARS-CoV-2 virus target genes in various cancers. The outcomes demonstrated a correlation between the expression of SARS-CoV-2 target genes and tumor prognosis, immune and drug sensitivity. In addition, there are a number of intriguing mechanisms linking SARS-CoV-2 virus target genes to cancer-related pathways. SARS-CoV-2 virus and cancers may have a close relationship, and a novel and essential cancer therapy may be derived from the SARS-CoV-2 virus and immunity.

## Data availability statement

Publicly available datasets were analyzed in this study. This data can be found here: https://portal.gdc.cancer.gov/.

## Author contributions

YLia and ZH designed the project. YLia and JW performed the analysis. JW, YLia, JZ and YLiu wrote the manuscript with the supervision of ZL and ZH. All authors approved the submitted version.
