# Supplementary material for: Multi-omics analysis reveals genomic, clinical and immunological features of SARS-CoV-2 virus target genes in pan-cancer
Source: Front Immunol. 2023 Feb 17;14:1112704. doi: 10.3389/fimmu.2023.1112704 (PMC9982007; doi:10.3389/fimmu.2023.1112704)
Supplement: Supplementary file 1 [file Table_1.doc]

**Multi-omics analysis reveals genomic, clinical and immunological features of SARS-CoV-2 virus target genes in pan-cancer**

Yong Liao^#^, Jiaojiao Wang^#^, Jiami Zou^#^, Yong Liu, Zhiping Liu^*^, Zunnan Huang^*^

**Table S1.** Human primers

| **Gene** | **Primers** |
| --- | --- |
| ***ACE2*** | Forward：CGAAGCCGAAGACCTGTTCTA |
|  | Reverse：GGGCAAGTGTGGACTGTTCC |
| ***NRP1*** | Forward：TCGCATTTTTCACTTGGGTGAT |
|  | Reverse：GGCGCTTTTCGCAACGATAAA |
| ***SCARB1*** | Forward：CCTATCCCCTTCTATCTCTCCG |
|  | Reverse：GGATGTTGGGCATGACGATGT |
| ***AXL*** | Forward：GTGGGCAACCCAGGGAATATC |
|  | Reverse：GTACTGTCCCGTGTCGGAAAG |
| ***TMPRSS2*** | Forward：GTCCCCACTGTCTACGAGGT |
|  | Reverse：CAGACGACGGGGTTGGAAG |

Table S2. DifferentialExpressionTable

| cancertype | symbol | tumor | normal | fc | pval | fdr | n_tumor | n_normal |
| --- | --- | --- | --- | --- | --- | --- | --- | --- |
| BLCA | ACE2 | 86.6667 | 86.9658 | 0.9966 | 0.9947 | 0.9966 | 19 | 19 |
| BLCA | AXL | 713.0885 | 2385.9306 | 0.2989 | 0.0002 | 0.0038 | 19 | 19 |
| BLCA | NRP1 | 1082.7064 | 1610.4891 | 0.6723 | 0.0261 | 0.0873 | 19 | 19 |
| BLCA | SCARB1 | 1114.0975 | 632.8115 | 1.7604 | 0.0075 | 0.0363 | 19 | 19 |
| BLCA | TMPRSS2 | 1582.8318 | 1074.6080 | 1.4729 | 0.3128 | 0.4673 | 19 | 19 |
| BRCA | ACE2 | 43.7550 | 30.9472 | 1.4125 | 0.4095 | 0.4690 | 114 | 114 |
| BRCA | AXL | 1335.8521 | 1902.2113 | 0.7023 | 0.0000 | 0.0000 | 114 | 114 |
| BRCA | NRP1 | 2553.7220 | 4912.5910 | 0.5198 | 0.0000 | 0.0000 | 114 | 114 |
| BRCA | SCARB1 | 1678.2644 | 2079.6261 | 0.8070 | 0.0035 | 0.0064 | 114 | 114 |
| BRCA | TMPRSS2 | 413.0251 | 887.9333 | 0.4652 | 0.0000 | 0.0000 | 114 | 114 |
| COAD | ACE2 | 957.8254 | 698.4534 | 1.3713 | 0.3197 | 0.4073 | 26 | 26 |
| COAD | AXL | 623.1880 | 1318.2195 | 0.4728 | 0.0011 | 0.0033 | 26 | 26 |
| COAD | NRP1 | 1004.8600 | 1213.6018 | 0.8280 | 0.1696 | 0.2424 | 26 | 26 |
| COAD | SCARB1 | 2098.0927 | 1228.1858 | 1.7082 | 0.0000 | 0.0001 | 26 | 26 |
| COAD | TMPRSS2 | 3604.6822 | 10130.0198 | 0.3558 | 0.0000 | 0.0000 | 26 | 26 |
| ESCA | ACE2 | 654.2742 | 64.6498 | 10.1062 | 0.2325 | 0.4313 | 11 | 11 |
| ESCA | AXL | 1106.9665 | 2061.1180 | 0.5371 | 0.2014 | 0.3992 | 11 | 11 |
| ESCA | NRP1 | 1847.0629 | 1682.4504 | 1.0978 | 0.8124 | 0.8878 | 11 | 11 |
| ESCA | SCARB1 | 1565.4057 | 428.8536 | 3.6496 | 0.0034 | 0.0481 | 11 | 11 |
| ESCA | TMPRSS2 | 3069.3491 | 5966.6705 | 0.5144 | 0.1054 | 0.2814 | 11 | 11 |
| HNSC | ACE2 | 88.5563 | 115.4274 | 0.7674 | 0.1106 | 0.1861 | 43 | 43 |
| HNSC | AXL | 1285.6518 | 676.4039 | 1.9006 | 0.0083 | 0.0225 | 43 | 43 |
| HNSC | NRP1 | 1497.7459 | 1327.7309 | 1.1280 | 0.4196 | 0.5198 | 43 | 43 |
| HNSC | SCARB1 | 967.1829 | 564.9332 | 1.7119 | 0.0051 | 0.0152 | 43 | 43 |
| HNSC | TMPRSS2 | 263.6102 | 1387.3585 | 0.1901 | 0.0000 | 0.0000 | 43 | 43 |
| KICH | ACE2 | 4.8151 | 815.0469 | 0.0060 | 0.0005 | 0.0016 | 25 | 25 |
| KICH | AXL | 671.7481 | 624.1902 | 1.0762 | 0.8795 | 0.9031 | 25 | 25 |
| KICH | NRP1 | 1929.0823 | 2919.9159 | 0.6607 | 0.1371 | 0.1995 | 25 | 25 |
| KICH | SCARB1 | 568.5729 | 261.3826 | 2.1748 | 0.0000 | 0.0000 | 25 | 25 |
| KICH | TMPRSS2 | 6743.7763 | 2985.8464 | 2.2585 | 0.0000 | 0.0001 | 25 | 25 |
| KIRC | ACE2 | 2583.9938 | 1505.2982 | 1.7166 | 0.0015 | 0.0028 | 72 | 72 |
| KIRC | AXL | 2692.5846 | 878.1448 | 3.0660 | 0.0000 | 0.0000 | 72 | 72 |
| KIRC | NRP1 | 10831.7992 | 4810.9085 | 2.2515 | 0.0000 | 0.0000 | 72 | 72 |
| KIRC | SCARB1 | 5351.2307 | 379.3114 | 14.1043 | 0.0000 | 0.0000 | 72 | 72 |
| KIRC | TMPRSS2 | 116.6373 | 2744.7067 | 0.0425 | 0.0000 | 0.0000 | 72 | 72 |
| KIRP | ACE2 | 2541.1759 | 1828.5420 | 1.3897 | 0.1890 | 0.2814 | 32 | 32 |
| KIRP | AXL | 1865.1734 | 692.3006 | 2.6939 | 0.0001 | 0.0004 | 32 | 32 |
| KIRP | NRP1 | 4161.4641 | 3783.0270 | 1.1000 | 0.5332 | 0.6216 | 32 | 32 |
| KIRP | SCARB1 | 2177.0629 | 326.2154 | 6.6720 | 0.0012 | 0.0043 | 32 | 32 |
| KIRP | TMPRSS2 | 335.6300 | 2736.0557 | 0.1227 | 0.0000 | 0.0000 | 32 | 32 |
| LIHC | ACE2 | 153.3388 | 171.2664 | 0.8954 | 0.7146 | 0.7708 | 50 | 50 |
| LIHC | AXL | 351.0017 | 777.2661 | 0.4517 | 0.0000 | 0.0000 | 50 | 50 |
| LIHC | NRP1 | 2583.5523 | 2190.5590 | 1.1794 | 0.0752 | 0.1319 | 50 | 50 |
| LIHC | SCARB1 | 11564.5927 | 10012.3731 | 1.1550 | 0.1841 | 0.2677 | 50 | 50 |
| LIHC | TMPRSS2 | 1133.5900 | 2351.2671 | 0.4821 | 0.0000 | 0.0002 | 50 | 50 |
| LUAD | ACE2 | 180.6979 | 59.3060 | 3.0434 | 0.0009 | 0.0022 | 58 | 58 |
| LUAD | AXL | 1579.2428 | 2436.0366 | 0.6483 | 0.0002 | 0.0005 | 58 | 58 |
| LUAD | NRP1 | 4196.8091 | 4252.0435 | 0.9870 | 0.8694 | 0.8920 | 58 | 58 |
| LUAD | SCARB1 | 988.1336 | 916.2641 | 1.0784 | 0.5094 | 0.5700 | 58 | 58 |
| LUAD | TMPRSS2 | 2046.6836 | 3886.2681 | 0.5267 | 0.0000 | 0.0000 | 58 | 58 |
| LUSC | ACE2 | 117.7594 | 67.0441 | 1.7553 | 0.1033 | 0.1412 | 51 | 51 |
| LUSC | AXL | 1303.8224 | 2513.6914 | 0.5187 | 0.0000 | 0.0000 | 51 | 51 |
| LUSC | NRP1 | 1851.8104 | 4653.0463 | 0.3980 | 0.0000 | 0.0000 | 51 | 51 |
| LUSC | SCARB1 | 1151.7017 | 1056.9018 | 1.0897 | 0.3635 | 0.4200 | 51 | 51 |
| LUSC | TMPRSS2 | 697.1141 | 3788.0625 | 0.1841 | 0.0000 | 0.0000 | 51 | 51 |
| PRAD | ACE2 | 10.2703 | 169.4410 | 0.0612 | 0.2401 | 0.3406 | 52 | 52 |
| PRAD | AXL | 707.6484 | 1134.0499 | 0.6240 | 0.0000 | 0.0000 | 52 | 52 |
| PRAD | NRP1 | 2114.6561 | 1738.1707 | 1.2166 | 0.2060 | 0.3015 | 52 | 52 |
| PRAD | SCARB1 | 1611.4695 | 921.0144 | 1.7496 | 0.0000 | 0.0000 | 52 | 52 |
| PRAD | TMPRSS2 | 34770.6061 | 23207.0722 | 1.4983 | 0.0002 | 0.0009 | 52 | 52 |
| STAD | ACE2 | 473.8498 | 3605.1028 | 0.1315 | 0.0539 | 0.1227 | 32 | 32 |
| STAD | AXL | 1649.2170 | 2277.7465 | 0.7241 | 0.2555 | 0.3858 | 32 | 32 |
| STAD | NRP1 | 1837.4012 | 1286.1393 | 1.4286 | 0.0413 | 0.1008 | 32 | 32 |
| STAD | SCARB1 | 1686.3980 | 990.5119 | 1.7025 | 0.1105 | 0.2075 | 32 | 32 |
| STAD | TMPRSS2 | 2664.2752 | 3742.4064 | 0.7119 | 0.1113 | 0.2087 | 32 | 32 |
| THCA | ACE2 | 43.4308 | 54.3495 | 0.7995 | 0.0109 | 0.0219 | 59 | 59 |
| THCA | AXL | 1042.3636 | 743.6202 | 1.4017 | 0.0000 | 0.0001 | 59 | 59 |
| THCA | NRP1 | 2261.9744 | 1990.5435 | 1.1364 | 0.0667 | 0.1087 | 59 | 59 |
| THCA | SCARB1 | 700.7444 | 593.3181 | 1.1810 | 0.0852 | 0.1343 | 59 | 59 |
| THCA | TMPRSS2 | 742.8893 | 506.5300 | 1.4665 | 0.0514 | 0.0863 | 59 | 59 |

Table S3. ExpressionAndSurvivalTable

| cancertype | symbol | sur_type | hr_categorical(H/L) | coxp_categorical | logrankp | higher_risk_of_death |
| --- | --- | --- | --- | --- | --- | --- |
| ACC | ACE2 | OS | 0.5383 | 0.1111 | 0.1060 | Lower expr. |
| ACC | ACE2 | PFS | 0.6874 | 0.2261 | 0.2231 | Lower expr. |
| ACC | ACE2 | DSS | 0.5418 | 0.1294 | 0.1241 | Lower expr. |
| ACC | ACE2 | DFI | 0.2154 | 0.0521 | 0.0337 | Lower expr. |
| ACC | AXL | OS | 0.8117 | 0.5832 | 0.5842 | Lower expr. |
| ACC | AXL | PFS | 0.7488 | 0.3454 | 0.3461 | Lower expr. |
| ACC | AXL | DSS | 0.7205 | 0.4104 | 0.4096 | Lower expr. |
| ACC | AXL | DFI | 0.9134 | 0.8815 | 0.8814 | Lower expr. |
| ACC | NRP1 | OS | 1.9742 | 0.0831 | 0.0776 | Higher expr. |
| ACC | NRP1 | PFS | 1.7301 | 0.0815 | 0.0773 | Higher expr. |
| ACC | NRP1 | DSS | 1.7979 | 0.1446 | 0.1390 | Higher expr. |
| ACC | NRP1 | DFI | 4.0427 | 0.0418 | 0.0285 | Higher expr. |
| ACC | SCARB1 | OS | 1.5959 | 0.2283 | 0.2250 | Higher expr. |
| ACC | SCARB1 | PFS | 2.0205 | 0.0268 | 0.0239 | Higher expr. |
| ACC | SCARB1 | DSS | 1.6964 | 0.1908 | 0.1865 | Higher expr. |
| ACC | SCARB1 | DFI | 1.6609 | 0.4046 | 0.3998 | Higher expr. |
| ACC | TMPRSS2 | OS | 1.9461 | 0.1005 | 0.0947 | Higher expr. |
| ACC | TMPRSS2 | PFS | 2.2846 | 0.0138 | 0.0112 | Higher expr. |
| ACC | TMPRSS2 | DSS | 1.8271 | 0.1567 | 0.1510 | Higher expr. |
| ACC | TMPRSS2 | DFI | 2.8299 | 0.1281 | 0.1119 | Higher expr. |
| BLCA | ACE2 | OS | 0.9563 | 0.7662 | 0.7659 | Lower expr. |
| BLCA | ACE2 | PFS | 0.8831 | 0.3479 | 0.3477 | Lower expr. |
| BLCA | ACE2 | DSS | 0.8908 | 0.5261 | 0.5262 | Lower expr. |
| BLCA | ACE2 | DFI | 0.9944 | 0.9876 | 0.9876 | Lower expr. |
| BLCA | AXL | OS | 1.3382 | 0.0536 | 0.0525 | Higher expr. |
| BLCA | AXL | PFS | 1.2702 | 0.0718 | 0.0712 | Higher expr. |
| BLCA | AXL | DSS | 1.3885 | 0.0728 | 0.0715 | Higher expr. |
| BLCA | AXL | DFI | 1.0418 | 0.9105 | 0.9105 | Higher expr. |
| BLCA | NRP1 | OS | 1.2074 | 0.2096 | 0.2085 | Higher expr. |
| BLCA | NRP1 | PFS | 1.0969 | 0.4858 | 0.4856 | Higher expr. |
| BLCA | NRP1 | DSS | 1.2509 | 0.2191 | 0.2184 | Higher expr. |
| BLCA | NRP1 | DFI | 0.7113 | 0.3532 | 0.3509 | Lower expr. |
| BLCA | SCARB1 | OS | 1.0511 | 0.7399 | 0.7407 | Higher expr. |
| BLCA | SCARB1 | PFS | 1.0609 | 0.6562 | 0.6573 | Higher expr. |
| BLCA | SCARB1 | DSS | 1.0953 | 0.6170 | 0.6171 | Higher expr. |
| BLCA | SCARB1 | DFI | 0.9850 | 0.9666 | 0.9666 | Lower expr. |
| BLCA | TMPRSS2 | OS | 0.7654 | 0.0770 | 0.0763 | Lower expr. |
| BLCA | TMPRSS2 | PFS | 0.8291 | 0.1584 | 0.1577 | Lower expr. |
| BLCA | TMPRSS2 | DSS | 0.6849 | 0.0407 | 0.0396 | Lower expr. |
| BLCA | TMPRSS2 | DFI | 0.8836 | 0.7320 | 0.7318 | Lower expr. |
| BRCA | ACE2 | OS | 0.9101 | 0.5596 | 0.5578 | Lower expr. |
| BRCA | ACE2 | PFS | 0.8744 | 0.3349 | 0.3332 | Lower expr. |
| BRCA | ACE2 | DSS | 1.0019 | 0.9929 | 0.9951 | Higher expr. |
| BRCA | ACE2 | DFI | 0.8555 | 0.4755 | 0.4751 | Lower expr. |
| BRCA | AXL | OS | 1.0052 | 0.9744 | 0.9762 | Higher expr. |
| BRCA | AXL | PFS | 0.9201 | 0.5550 | 0.5534 | Lower expr. |
| BRCA | AXL | DSS | 1.2017 | 0.4006 | 0.4007 | Higher expr. |
| BRCA | AXL | DFI | 0.8811 | 0.5644 | 0.5641 | Lower expr. |
| BRCA | NRP1 | OS | 0.9414 | 0.7087 | 0.7066 | Lower expr. |
| BRCA | NRP1 | PFS | 0.9886 | 0.9344 | 0.9321 | Lower expr. |
| BRCA | NRP1 | DSS | 1.0494 | 0.8249 | 0.8278 | Higher expr. |
| BRCA | NRP1 | DFI | 1.1051 | 0.6482 | 0.6481 | Higher expr. |
| BRCA | SCARB1 | OS | 0.8986 | 0.5104 | 0.5101 | Lower expr. |
| BRCA | SCARB1 | PFS | 0.9262 | 0.5847 | 0.5851 | Lower expr. |
| BRCA | SCARB1 | DSS | 0.8562 | 0.4780 | 0.4777 | Lower expr. |
| BRCA | SCARB1 | DFI | 1.1616 | 0.4961 | 0.4957 | Higher expr. |
| BRCA | TMPRSS2 | OS | 1.5246 | 0.0105 | 0.0100 | Higher expr. |
| BRCA | TMPRSS2 | PFS | 1.1913 | 0.2106 | 0.2105 | Higher expr. |
| BRCA | TMPRSS2 | DSS | 1.7676 | 0.0112 | 0.0103 | Higher expr. |
| BRCA | TMPRSS2 | DFI | 0.9862 | 0.9492 | 0.9492 | Lower expr. |
| CESC | ACE2 | OS | 0.8554 | 0.5069 | 0.5055 | Lower expr. |
| CESC | ACE2 | PFS | 0.8733 | 0.5266 | 0.5259 | Lower expr. |
| CESC | ACE2 | DSS | 0.8104 | 0.4383 | 0.4371 | Lower expr. |
| CESC | ACE2 | DFI | 0.7063 | 0.3826 | 0.3808 | Lower expr. |
| CESC | AXL | OS | 1.1608 | 0.5270 | 0.5265 | Higher expr. |
| CESC | AXL | PFS | 1.1829 | 0.4326 | 0.4323 | Higher expr. |
| CESC | AXL | DSS | 1.0259 | 0.9245 | 0.9245 | Higher expr. |
| CESC | AXL | DFI | 1.8284 | 0.1361 | 0.1306 | Higher expr. |
| CESC | NRP1 | OS | 1.7315 | 0.0222 | 0.0205 | Higher expr. |
| CESC | NRP1 | PFS | 1.9379 | 0.0027 | 0.0023 | Higher expr. |
| CESC | NRP1 | DSS | 1.9784 | 0.0153 | 0.0134 | Higher expr. |
| CESC | NRP1 | DFI | 2.8064 | 0.0125 | 0.0091 | Higher expr. |
| CESC | SCARB1 | OS | 1.3948 | 0.1596 | 0.1576 | Higher expr. |
| CESC | SCARB1 | PFS | 1.4629 | 0.0787 | 0.0768 | Higher expr. |
| CESC | SCARB1 | DSS | 1.2733 | 0.3730 | 0.3717 | Higher expr. |
| CESC | SCARB1 | DFI | 1.6381 | 0.2148 | 0.2102 | Higher expr. |
| CESC | TMPRSS2 | OS | 1.0918 | 0.7081 | 0.7082 | Higher expr. |
| CESC | TMPRSS2 | PFS | 1.1256 | 0.5796 | 0.5804 | Higher expr. |
| CESC | TMPRSS2 | DSS | 1.0033 | 0.9903 | 0.9899 | Higher expr. |
| CESC | TMPRSS2 | DFI | 1.2972 | 0.5127 | 0.5122 | Higher expr. |
| CHOL | ACE2 | OS | 0.8996 | 0.8231 | 0.8230 | Lower expr. |
| CHOL | ACE2 | PFS | 1.0459 | 0.9163 | 0.9163 | Higher expr. |
| CHOL | ACE2 | DSS | 1.1254 | 0.8153 | 0.8152 | Higher expr. |
| CHOL | ACE2 | DFI | 0.6200 | 0.4599 | 0.4557 | Lower expr. |
| CHOL | AXL | OS | 0.8055 | 0.6564 | 0.6558 | Lower expr. |
| CHOL | AXL | PFS | 0.7193 | 0.4434 | 0.4414 | Lower expr. |
| CHOL | AXL | DSS | 1.0126 | 0.9806 | 0.9806 | Higher expr. |
| CHOL | AXL | DFI | 1.2831 | 0.7010 | 0.7003 | Higher expr. |
| CHOL | NRP1 | OS | 0.9737 | 0.9554 | 0.9554 | Lower expr. |
| CHOL | NRP1 | PFS | 0.6835 | 0.3851 | 0.3824 | Lower expr. |
| CHOL | NRP1 | DSS | 0.9399 | 0.9024 | 0.9024 | Lower expr. |
| CHOL | NRP1 | DFI | 1.0319 | 0.9613 | 0.9613 | Higher expr. |
| CHOL | SCARB1 | OS | 0.8487 | 0.7317 | 0.7314 | Lower expr. |
| CHOL | SCARB1 | PFS | 0.9011 | 0.8084 | 0.8083 | Lower expr. |
| CHOL | SCARB1 | DSS | 0.8049 | 0.6696 | 0.6691 | Lower expr. |
| CHOL | SCARB1 | DFI | 0.9762 | 0.9697 | 0.9697 | Lower expr. |
| CHOL | TMPRSS2 | OS | 1.4795 | 0.4115 | 0.4086 | Higher expr. |
| CHOL | TMPRSS2 | PFS | 1.6915 | 0.2254 | 0.2204 | Higher expr. |
| CHOL | TMPRSS2 | DSS | 1.5188 | 0.4100 | 0.4068 | Higher expr. |
| CHOL | TMPRSS2 | DFI | 1.2301 | 0.7470 | 0.7466 | Higher expr. |
| COAD | ACE2 | OS | 0.7119 | 0.1680 | 0.1660 | Lower expr. |
| COAD | ACE2 | PFS | 0.7117 | 0.0832 | 0.0817 | Lower expr. |
| COAD | ACE2 | DSS | 0.6479 | 0.2003 | 0.1968 | Lower expr. |
| COAD | ACE2 | DFI | 0.7837 | 0.6265 | 0.6256 | Lower expr. |
| COAD | AXL | OS | 1.4367 | 0.1348 | 0.1327 | Higher expr. |
| COAD | AXL | PFS | 1.2550 | 0.2418 | 0.2404 | Higher expr. |
| COAD | AXL | DSS | 2.2792 | 0.0200 | 0.0167 | Higher expr. |
| COAD | AXL | DFI | 0.9761 | 0.9618 | 0.9618 | Lower expr. |
| COAD | NRP1 | OS | 1.5481 | 0.0726 | 0.0704 | Higher expr. |
| COAD | NRP1 | PFS | 1.3601 | 0.1145 | 0.1128 | Higher expr. |
| COAD | NRP1 | DSS | 2.5065 | 0.0098 | 0.0075 | Higher expr. |
| COAD | NRP1 | DFI | 1.0256 | 0.9600 | 0.9600 | Higher expr. |
| COAD | SCARB1 | OS | 0.8388 | 0.4739 | 0.4733 | Lower expr. |
| COAD | SCARB1 | PFS | 1.0114 | 0.9535 | 0.9536 | Higher expr. |
| COAD | SCARB1 | DSS | 0.6940 | 0.2871 | 0.2845 | Lower expr. |
| COAD | SCARB1 | DFI | 1.5970 | 0.3663 | 0.3620 | Higher expr. |
| COAD | TMPRSS2 | OS | 0.9225 | 0.7382 | 0.7382 | Lower expr. |
| COAD | TMPRSS2 | PFS | 1.0220 | 0.9106 | 0.9119 | Higher expr. |
| COAD | TMPRSS2 | DSS | 0.7994 | 0.5045 | 0.5036 | Lower expr. |
| COAD | TMPRSS2 | DFI | 0.6629 | 0.4272 | 0.4240 | Lower expr. |
| DLBC | ACE2 | OS | 1.4497 | 0.6082 | 0.6063 | Higher expr. |
| DLBC | ACE2 | PFS | 1.8004 | 0.2714 | 0.2655 | Higher expr. |
| DLBC | ACE2 | DSS | 0.4203 | 0.4554 | 0.4418 | Lower expr. |
| DLBC | ACE2 | DFI | 6.5057 | 0.1314 | 0.0853 | Higher expr. |
| DLBC | AXL | OS | 3.2149 | 0.1537 | 0.1317 | Higher expr. |
| DLBC | AXL | PFS | 2.0878 | 0.1828 | 0.1735 | Higher expr. |
| DLBC | AXL | DSS | 3.2377 | 0.3096 | 0.2824 | Higher expr. |
| DLBC | AXL | DFI | 1.5175 | 0.7404 | 0.7388 | Higher expr. |
| DLBC | NRP1 | OS | 1.3060 | 0.7197 | 0.7190 | Higher expr. |
| DLBC | NRP1 | PFS | 0.8140 | 0.7057 | 0.7053 | Lower expr. |
| DLBC | NRP1 | DSS | 2.6274 | 0.4063 | 0.3889 | Higher expr. |
| DLBC | NRP1 | DFI | 0.0000 | 0.9994 | 0.0487 | Lower expr. |
| DLBC | SCARB1 | OS | 0.2972 | 0.1386 | 0.1161 | Lower expr. |
| DLBC | SCARB1 | PFS | 0.3147 | 0.0485 | 0.0374 | Lower expr. |
| DLBC | SCARB1 | DSS | 0.2801 | 0.2708 | 0.2393 | Lower expr. |
| DLBC | SCARB1 | DFI | 0.0000 | 0.9993 | 0.0887 | Lower expr. |
| DLBC | TMPRSS2 | OS | 0.8684 | 0.8454 | 0.8453 | Lower expr. |
| DLBC | TMPRSS2 | PFS | 0.7450 | 0.5803 | 0.5792 | Lower expr. |
| DLBC | TMPRSS2 | DSS | 0.9326 | 0.9445 | 0.9445 | Lower expr. |
| DLBC | TMPRSS2 | DFI | 1.1853 | 0.8927 | 0.8926 | Higher expr. |
| ESCA | ACE2 | OS | 1.3310 | 0.2087 | 0.2065 | Higher expr. |
| ESCA | ACE2 | PFS | 1.2051 | 0.3207 | 0.3199 | Higher expr. |
| ESCA | ACE2 | DSS | 1.0942 | 0.7478 | 0.7478 | Higher expr. |
| ESCA | ACE2 | DFI | 1.2009 | 0.6639 | 0.6634 | Higher expr. |
| ESCA | AXL | OS | 0.6617 | 0.0751 | 0.0732 | Lower expr. |
| ESCA | AXL | PFS | 0.7957 | 0.2251 | 0.2248 | Lower expr. |
| ESCA | AXL | DSS | 0.9349 | 0.8090 | 0.8098 | Lower expr. |
| ESCA | AXL | DFI | 1.2780 | 0.5756 | 0.5746 | Higher expr. |
| ESCA | NRP1 | OS | 0.9590 | 0.8545 | 0.8537 | Lower expr. |
| ESCA | NRP1 | PFS | 1.0233 | 0.9024 | 0.9005 | Higher expr. |
| ESCA | NRP1 | DSS | 1.0241 | 0.9318 | 0.9310 | Higher expr. |
| ESCA | NRP1 | DFI | 2.0986 | 0.0971 | 0.0905 | Higher expr. |
| ESCA | SCARB1 | OS | 1.1133 | 0.6389 | 0.6389 | Higher expr. |
| ESCA | SCARB1 | PFS | 1.0563 | 0.7718 | 0.7725 | Higher expr. |
| ESCA | SCARB1 | DSS | 1.0806 | 0.7805 | 0.7797 | Higher expr. |
| ESCA | SCARB1 | DFI | 0.4740 | 0.1165 | 0.1082 | Lower expr. |
| ESCA | TMPRSS2 | OS | 1.0179 | 0.9397 | 0.9386 | Higher expr. |
| ESCA | TMPRSS2 | PFS | 0.9501 | 0.7881 | 0.7880 | Lower expr. |
| ESCA | TMPRSS2 | DSS | 0.9873 | 0.9648 | 0.9655 | Lower expr. |
| ESCA | TMPRSS2 | DFI | 0.5643 | 0.2655 | 0.2597 | Lower expr. |
| GBM | ACE2 | OS | 1.3416 | 0.0937 | 0.0920 | Higher expr. |
| GBM | ACE2 | PFS | 1.2999 | 0.1214 | 0.1189 | Higher expr. |
| GBM | ACE2 | DSS | 1.3590 | 0.0997 | 0.0978 | Higher expr. |
| GBM | AXL | OS | 1.0583 | 0.7469 | 0.7389 | Higher expr. |
| GBM | AXL | PFS | 1.1037 | 0.5587 | 0.5595 | Higher expr. |
| GBM | AXL | DSS | 1.0325 | 0.8639 | 0.8566 | Higher expr. |
| GBM | NRP1 | OS | 1.3271 | 0.1036 | 0.1071 | Higher expr. |
| GBM | NRP1 | PFS | 1.3576 | 0.0677 | 0.0683 | Higher expr. |
| GBM | NRP1 | DSS | 1.3906 | 0.0739 | 0.0763 | Higher expr. |
| GBM | SCARB1 | OS | 1.0475 | 0.7914 | 0.7614 | Higher expr. |
| GBM | SCARB1 | PFS | 1.0603 | 0.7254 | 0.7157 | Higher expr. |
| GBM | SCARB1 | DSS | 1.0171 | 0.9275 | 0.8950 | Higher expr. |
| GBM | TMPRSS2 | OS | 0.9687 | 0.8555 | 0.8455 | Lower expr. |
| GBM | TMPRSS2 | PFS | 0.9060 | 0.5519 | 0.5500 | Lower expr. |
| GBM | TMPRSS2 | DSS | 0.9662 | 0.8536 | 0.8443 | Lower expr. |
| HNSC | ACE2 | OS | 0.8050 | 0.1092 | 0.1085 | Lower expr. |
| HNSC | ACE2 | PFS | 0.8612 | 0.2195 | 0.2190 | Lower expr. |
| HNSC | ACE2 | DSS | 0.7358 | 0.0823 | 0.0811 | Lower expr. |
| HNSC | ACE2 | DFI | 1.0793 | 0.8439 | 0.8438 | Higher expr. |
| HNSC | AXL | OS | 1.1378 | 0.3400 | 0.3393 | Higher expr. |
| HNSC | AXL | PFS | 1.0715 | 0.5704 | 0.5703 | Higher expr. |
| HNSC | AXL | DSS | 1.1888 | 0.3261 | 0.3252 | Higher expr. |
| HNSC | AXL | DFI | 1.4198 | 0.3594 | 0.3570 | Higher expr. |
| HNSC | NRP1 | OS | 1.0661 | 0.6366 | 0.6365 | Higher expr. |
| HNSC | NRP1 | PFS | 1.0407 | 0.7432 | 0.7437 | Higher expr. |
| HNSC | NRP1 | DSS | 1.2184 | 0.2656 | 0.2648 | Higher expr. |
| HNSC | NRP1 | DFI | 1.2982 | 0.5052 | 0.5040 | Higher expr. |
| HNSC | SCARB1 | OS | 1.4194 | 0.0101 | 0.0097 | Higher expr. |
| HNSC | SCARB1 | PFS | 1.2772 | 0.0451 | 0.0446 | Higher expr. |
| HNSC | SCARB1 | DSS | 1.4859 | 0.0255 | 0.0245 | Higher expr. |
| HNSC | SCARB1 | DFI | 0.9447 | 0.8857 | 0.8857 | Lower expr. |
| HNSC | TMPRSS2 | OS | 0.8054 | 0.1125 | 0.1117 | Lower expr. |
| HNSC | TMPRSS2 | PFS | 0.8170 | 0.1000 | 0.0994 | Lower expr. |
| HNSC | TMPRSS2 | DSS | 0.6216 | 0.0084 | 0.0078 | Lower expr. |
| HNSC | TMPRSS2 | DFI | 0.9098 | 0.8056 | 0.8055 | Lower expr. |
| KICH | ACE2 | OS | 0.4751 | 0.2928 | 0.2817 | Lower expr. |
| KICH | ACE2 | PFS | 0.2891 | 0.0628 | 0.0474 | Lower expr. |
| KICH | ACE2 | DSS | 0.3783 | 0.2456 | 0.2273 | Lower expr. |
| KICH | ACE2 | DFI | 0.0000 | 0.9993 | 0.0409 | Lower expr. |
| KICH | AXL | OS | 0.5409 | 0.3849 | 0.3775 | Lower expr. |
| KICH | AXL | PFS | 0.5250 | 0.2929 | 0.2846 | Lower expr. |
| KICH | AXL | DSS | 0.4296 | 0.3127 | 0.2984 | Lower expr. |
| KICH | AXL | DFI | 0.6007 | 0.6774 | 0.6741 | Lower expr. |
| KICH | NRP1 | OS | 1.3510 | 0.6540 | 0.6527 | Higher expr. |
| KICH | NRP1 | PFS | 1.5293 | 0.4683 | 0.4650 | Higher expr. |
| KICH | NRP1 | DSS | 1.4435 | 0.6309 | 0.6290 | Higher expr. |
| KICH | NRP1 | DFI | 1.5222 | 0.7318 | 0.7299 | Higher expr. |
| KICH | SCARB1 | OS | 0.8336 | 0.7864 | 0.7861 | Lower expr. |
| KICH | SCARB1 | PFS | 1.0312 | 0.9576 | 0.9576 | Higher expr. |
| KICH | SCARB1 | DSS | 0.7603 | 0.7201 | 0.7193 | Lower expr. |
| KICH | SCARB1 | DFI | 1.7623 | 0.6440 | 0.6396 | Higher expr. |
| KICH | TMPRSS2 | OS | 0.1187 | 0.0446 | 0.0160 | Lower expr. |
| KICH | TMPRSS2 | PFS | 0.1859 | 0.0299 | 0.0148 | Lower expr. |
| KICH | TMPRSS2 | DSS | 0.1534 | 0.0827 | 0.0455 | Lower expr. |
| KICH | TMPRSS2 | DFI | 0.6007 | 0.6774 | 0.6741 | Lower expr. |
| KIRC | ACE2 | OS | 0.5873 | 0.0006 | 0.0005 | Lower expr. |
| KIRC | ACE2 | PFS | 0.6172 | 0.0004 | 0.0003 | Lower expr. |
| KIRC | ACE2 | DSS | 0.4013 | 0.0000 | 0.0000 | Lower expr. |
| KIRC | ACE2 | DFI | 0.6467 | 0.4006 | 0.3970 | Lower expr. |
| KIRC | AXL | OS | 1.7564 | 0.0003 | 0.0002 | Higher expr. |
| KIRC | AXL | PFS | 1.6241 | 0.0004 | 0.0003 | Higher expr. |
| KIRC | AXL | DSS | 2.0935 | 0.0002 | 0.0002 | Higher expr. |
| KIRC | AXL | DFI | 1.3848 | 0.5391 | 0.5374 | Higher expr. |
| KIRC | NRP1 | OS | 0.6400 | 0.0039 | 0.0036 | Lower expr. |
| KIRC | NRP1 | PFS | 0.6986 | 0.0082 | 0.0079 | Lower expr. |
| KIRC | NRP1 | DSS | 0.6766 | 0.0441 | 0.0427 | Lower expr. |
| KIRC | NRP1 | DFI | 0.9443 | 0.9135 | 0.9135 | Lower expr. |
| KIRC | SCARB1 | OS | 0.7625 | 0.0749 | 0.0739 | Lower expr. |
| KIRC | SCARB1 | PFS | 0.8990 | 0.4277 | 0.4273 | Lower expr. |
| KIRC | SCARB1 | DSS | 0.7399 | 0.1180 | 0.1165 | Lower expr. |
| KIRC | SCARB1 | DFI | 1.5200 | 0.4297 | 0.4265 | Higher expr. |
| KIRC | TMPRSS2 | OS | 1.1144 | 0.4767 | 0.4768 | Higher expr. |
| KIRC | TMPRSS2 | PFS | 1.1956 | 0.1847 | 0.1842 | Higher expr. |
| KIRC | TMPRSS2 | DSS | 1.0993 | 0.6233 | 0.6239 | Higher expr. |
| KIRC | TMPRSS2 | DFI | 1.0963 | 0.8617 | 0.8617 | Higher expr. |
| KIRP | ACE2 | OS | 0.5875 | 0.0902 | 0.0865 | Lower expr. |
| KIRP | ACE2 | PFS | 0.9640 | 0.8761 | 0.8757 | Lower expr. |
| KIRP | ACE2 | DSS | 0.3936 | 0.0259 | 0.0210 | Lower expr. |
| KIRP | ACE2 | DFI | 1.4329 | 0.3437 | 0.3416 | Higher expr. |
| KIRP | AXL | OS | 0.5052 | 0.0320 | 0.0288 | Lower expr. |
| KIRP | AXL | PFS | 0.4709 | 0.0023 | 0.0018 | Lower expr. |
| KIRP | AXL | DSS | 0.2286 | 0.0028 | 0.0011 | Lower expr. |
| KIRP | AXL | DFI | 0.5812 | 0.1590 | 0.1541 | Lower expr. |
| KIRP | NRP1 | OS | 1.2020 | 0.5449 | 0.5443 | Higher expr. |
| KIRP | NRP1 | PFS | 1.6568 | 0.0370 | 0.0352 | Higher expr. |
| KIRP | NRP1 | DSS | 0.9490 | 0.8903 | 0.8902 | Lower expr. |
| KIRP | NRP1 | DFI | 1.5639 | 0.2506 | 0.2471 | Higher expr. |
| KIRP | SCARB1 | OS | 1.1796 | 0.5864 | 0.5859 | Higher expr. |
| KIRP | SCARB1 | PFS | 0.9762 | 0.9183 | 0.9183 | Lower expr. |
| KIRP | SCARB1 | DSS | 1.0780 | 0.8426 | 0.8425 | Higher expr. |
| KIRP | SCARB1 | DFI | 1.1128 | 0.7755 | 0.7753 | Higher expr. |
| KIRP | TMPRSS2 | OS | 0.7502 | 0.3442 | 0.3426 | Lower expr. |
| KIRP | TMPRSS2 | PFS | 0.8811 | 0.5905 | 0.5902 | Lower expr. |
| KIRP | TMPRSS2 | DSS | 0.6210 | 0.2186 | 0.2143 | Lower expr. |
| KIRP | TMPRSS2 | DFI | 1.2122 | 0.6132 | 0.6126 | Higher expr. |
| LAML | ACE2 | OS | 0.7716 | 0.1933 | 0.1875 | Lower expr. |
| LAML | AXL | OS | 0.7738 | 0.1930 | 0.1893 | Lower expr. |
| LAML | NRP1 | OS | 0.7125 | 0.0840 | 0.0817 | Lower expr. |
| LAML | SCARB1 | OS | 1.0351 | 0.8601 | 0.8504 | Higher expr. |
| LAML | TMPRSS2 | OS | 0.8945 | 0.6897 | 0.6879 | Lower expr. |
| LGG | ACE2 | OS | 1.3358 | 0.0939 | 0.0902 | Higher expr. |
| LGG | ACE2 | PFS | 1.0610 | 0.6621 | 0.6612 | Higher expr. |
| LGG | ACE2 | DSS | 1.4050 | 0.0618 | 0.0586 | Higher expr. |
| LGG | ACE2 | DFI | 1.1959 | 0.6628 | 0.6463 | Higher expr. |
| LGG | AXL | OS | 1.8705 | 0.0004 | 0.0003 | Higher expr. |
| LGG | AXL | PFS | 1.3136 | 0.0446 | 0.0440 | Higher expr. |
| LGG | AXL | DSS | 1.9232 | 0.0005 | 0.0003 | Higher expr. |
| LGG | AXL | DFI | 0.4452 | 0.0724 | 0.0671 | Lower expr. |
| LGG | NRP1 | OS | 1.4756 | 0.0288 | 0.0278 | Higher expr. |
| LGG | NRP1 | PFS | 1.2657 | 0.0843 | 0.0829 | Higher expr. |
| LGG | NRP1 | DSS | 1.5062 | 0.0286 | 0.0275 | Higher expr. |
| LGG | NRP1 | DFI | 0.5645 | 0.1767 | 0.1733 | Lower expr. |
| LGG | SCARB1 | OS | 0.4701 | 0.0000 | 0.0000 | Lower expr. |
| LGG | SCARB1 | PFS | 0.5341 | 0.0000 | 0.0000 | Lower expr. |
| LGG | SCARB1 | DSS | 0.4658 | 0.0001 | 0.0000 | Lower expr. |
| LGG | SCARB1 | DFI | 0.7213 | 0.4240 | 0.4122 | Lower expr. |
| LGG | TMPRSS2 | OS | 0.8246 | 0.2647 | 0.2586 | Lower expr. |
| LGG | TMPRSS2 | PFS | 0.8706 | 0.3082 | 0.3052 | Lower expr. |
| LGG | TMPRSS2 | DSS | 0.8398 | 0.3376 | 0.3299 | Lower expr. |
| LGG | TMPRSS2 | DFI | 0.8772 | 0.7494 | 0.7430 | Lower expr. |
| LIHC | ACE2 | OS | 0.6196 | 0.0074 | 0.0068 | Lower expr. |
| LIHC | ACE2 | PFS | 0.6902 | 0.0055 | 0.0053 | Lower expr. |
| LIHC | ACE2 | DSS | 0.4595 | 0.0008 | 0.0006 | Lower expr. |
| LIHC | ACE2 | DFI | 0.5190 | 0.0001 | 0.0001 | Lower expr. |
| LIHC | AXL | OS | 0.8844 | 0.4856 | 0.4848 | Lower expr. |
| LIHC | AXL | PFS | 0.8027 | 0.0987 | 0.0989 | Lower expr. |
| LIHC | AXL | DSS | 0.8051 | 0.3377 | 0.3365 | Lower expr. |
| LIHC | AXL | DFI | 0.7281 | 0.0575 | 0.0572 | Lower expr. |
| LIHC | NRP1 | OS | 1.0145 | 0.9348 | 0.9356 | Higher expr. |
| LIHC | NRP1 | PFS | 1.0728 | 0.5964 | 0.5970 | Higher expr. |
| LIHC | NRP1 | DSS | 0.9399 | 0.7837 | 0.7830 | Lower expr. |
| LIHC | NRP1 | DFI | 0.9831 | 0.9183 | 0.9182 | Lower expr. |
| LIHC | SCARB1 | OS | 1.4054 | 0.0563 | 0.0551 | Higher expr. |
| LIHC | SCARB1 | PFS | 1.0639 | 0.6404 | 0.6418 | Higher expr. |
| LIHC | SCARB1 | DSS | 1.6235 | 0.0368 | 0.0350 | Higher expr. |
| LIHC | SCARB1 | DFI | 1.0974 | 0.5746 | 0.5762 | Higher expr. |
| LIHC | TMPRSS2 | OS | 0.8612 | 0.3955 | 0.3958 | Lower expr. |
| LIHC | TMPRSS2 | PFS | 0.7412 | 0.0244 | 0.0240 | Lower expr. |
| LIHC | TMPRSS2 | DSS | 0.6441 | 0.0524 | 0.0507 | Lower expr. |
| LIHC | TMPRSS2 | DFI | 0.6284 | 0.0054 | 0.0050 | Lower expr. |
| LUAD | ACE2 | OS | 1.1296 | 0.4127 | 0.4121 | Higher expr. |
| LUAD | ACE2 | PFS | 1.1106 | 0.4005 | 0.3995 | Higher expr. |
| LUAD | ACE2 | DSS | 1.1316 | 0.5144 | 0.5139 | Higher expr. |
| LUAD | ACE2 | DFI | 1.0066 | 0.9753 | 0.9755 | Higher expr. |
| LUAD | AXL | OS | 0.9125 | 0.5365 | 0.5362 | Lower expr. |
| LUAD | AXL | PFS | 0.9629 | 0.7608 | 0.7595 | Lower expr. |
| LUAD | AXL | DSS | 0.9320 | 0.7099 | 0.7095 | Lower expr. |
| LUAD | AXL | DFI | 1.1981 | 0.3950 | 0.3948 | Higher expr. |
| LUAD | NRP1 | OS | 0.9106 | 0.5278 | 0.5278 | Lower expr. |
| LUAD | NRP1 | PFS | 0.9945 | 0.9646 | 0.9642 | Lower expr. |
| LUAD | NRP1 | DSS | 0.8710 | 0.4661 | 0.4656 | Lower expr. |
| LUAD | NRP1 | DFI | 1.0083 | 0.9689 | 0.9691 | Higher expr. |
| LUAD | SCARB1 | OS | 1.1458 | 0.3598 | 0.3595 | Higher expr. |
| LUAD | SCARB1 | PFS | 1.1258 | 0.3411 | 0.3411 | Higher expr. |
| LUAD | SCARB1 | DSS | 1.1214 | 0.5459 | 0.5454 | Higher expr. |
| LUAD | SCARB1 | DFI | 0.8141 | 0.3373 | 0.3360 | Lower expr. |
| LUAD | TMPRSS2 | OS | 0.6685 | 0.0075 | 0.0071 | Lower expr. |
| LUAD | TMPRSS2 | PFS | 0.7573 | 0.0264 | 0.0260 | Lower expr. |
| LUAD | TMPRSS2 | DSS | 0.6391 | 0.0207 | 0.0197 | Lower expr. |
| LUAD | TMPRSS2 | DFI | 0.8827 | 0.5558 | 0.5556 | Lower expr. |
| LUSC | ACE2 | OS | 0.7893 | 0.0876 | 0.0868 | Lower expr. |
| LUSC | ACE2 | PFS | 0.7896 | 0.0621 | 0.0614 | Lower expr. |
| LUSC | ACE2 | DSS | 0.6409 | 0.0413 | 0.0397 | Lower expr. |
| LUSC | ACE2 | DFI | 0.6510 | 0.0998 | 0.0972 | Lower expr. |
| LUSC | AXL | OS | 1.1782 | 0.2359 | 0.2353 | Higher expr. |
| LUSC | AXL | PFS | 1.1674 | 0.2204 | 0.2203 | Higher expr. |
| LUSC | AXL | DSS | 1.2162 | 0.3627 | 0.3619 | Higher expr. |
| LUSC | AXL | DFI | 1.5731 | 0.0802 | 0.0776 | Higher expr. |
| LUSC | NRP1 | OS | 1.1878 | 0.2157 | 0.2150 | Higher expr. |
| LUSC | NRP1 | PFS | 1.0974 | 0.4632 | 0.4629 | Higher expr. |
| LUSC | NRP1 | DSS | 1.4446 | 0.0907 | 0.0890 | Higher expr. |
| LUSC | NRP1 | DFI | 0.9154 | 0.7321 | 0.7324 | Lower expr. |
| LUSC | SCARB1 | OS | 1.0879 | 0.5410 | 0.5406 | Higher expr. |
| LUSC | SCARB1 | PFS | 1.1898 | 0.1685 | 0.1681 | Higher expr. |
| LUSC | SCARB1 | DSS | 1.3981 | 0.1243 | 0.1225 | Higher expr. |
| LUSC | SCARB1 | DFI | 1.6042 | 0.0698 | 0.0673 | Higher expr. |
| LUSC | TMPRSS2 | OS | 1.0333 | 0.8124 | 0.8130 | Higher expr. |
| LUSC | TMPRSS2 | PFS | 1.1659 | 0.2249 | 0.2248 | Higher expr. |
| LUSC | TMPRSS2 | DSS | 0.8890 | 0.5850 | 0.5848 | Lower expr. |
| LUSC | TMPRSS2 | DFI | 0.9751 | 0.9221 | 0.9220 | Lower expr. |
| MESO | ACE2 | OS | 0.6111 | 0.0416 | 0.0397 | Lower expr. |
| MESO | ACE2 | PFS | 0.5751 | 0.0223 | 0.0207 | Lower expr. |
| MESO | ACE2 | DSS | 0.6711 | 0.2008 | 0.1987 | Lower expr. |
| MESO | ACE2 | DFI | 0.1818 | 0.1679 | 0.1230 | Lower expr. |
| MESO | AXL | OS | 2.7824 | 0.0000 | 0.0000 | Higher expr. |
| MESO | AXL | PFS | 2.0464 | 0.0028 | 0.0024 | Higher expr. |
| MESO | AXL | DSS | 2.4982 | 0.0035 | 0.0025 | Higher expr. |
| MESO | AXL | DFI | 3.4940 | 0.1778 | 0.1531 | Higher expr. |
| MESO | NRP1 | OS | 1.4422 | 0.1291 | 0.1276 | Higher expr. |
| MESO | NRP1 | PFS | 1.1202 | 0.6232 | 0.6230 | Higher expr. |
| MESO | NRP1 | DSS | 1.5371 | 0.1674 | 0.1650 | Higher expr. |
| MESO | NRP1 | DFI | 2.4966 | 0.3243 | 0.3091 | Higher expr. |
| MESO | SCARB1 | OS | 0.8780 | 0.5874 | 0.5856 | Lower expr. |
| MESO | SCARB1 | PFS | 0.8779 | 0.5754 | 0.5764 | Lower expr. |
| MESO | SCARB1 | DSS | 1.1664 | 0.6197 | 0.6189 | Higher expr. |
| MESO | SCARB1 | DFI | 0.0000 | 0.9990 | 0.0082 | Lower expr. |
| MESO | TMPRSS2 | OS | 1.2364 | 0.3701 | 0.3698 | Higher expr. |
| MESO | TMPRSS2 | PFS | 1.3007 | 0.2560 | 0.2552 | Higher expr. |
| MESO | TMPRSS2 | DSS | 1.6021 | 0.1364 | 0.1334 | Higher expr. |
| MESO | TMPRSS2 | DFI | 7.4487 | 0.0867 | 0.0476 | Higher expr. |
| OV | ACE2 | OS | 0.8495 | 0.2728 | 0.2724 | Lower expr. |
| OV | ACE2 | PFS | 0.8423 | 0.1879 | 0.1872 | Lower expr. |
| OV | ACE2 | DSS | 0.8543 | 0.3311 | 0.3307 | Lower expr. |
| OV | ACE2 | DFI | 0.8884 | 0.5500 | 0.5515 | Lower expr. |
| OV | AXL | OS | 1.2145 | 0.1927 | 0.1931 | Higher expr. |
| OV | AXL | PFS | 0.9839 | 0.9011 | 0.8983 | Lower expr. |
| OV | AXL | DSS | 1.1223 | 0.4775 | 0.4796 | Higher expr. |
| OV | AXL | DFI | 0.9022 | 0.6044 | 0.5997 | Lower expr. |
| OV | NRP1 | OS | 1.0523 | 0.7308 | 0.7274 | Higher expr. |
| OV | NRP1 | PFS | 0.9884 | 0.9294 | 0.9298 | Lower expr. |
| OV | NRP1 | DSS | 1.0519 | 0.7541 | 0.7500 | Higher expr. |
| OV | NRP1 | DFI | 1.0524 | 0.7967 | 0.7940 | Higher expr. |
| OV | SCARB1 | OS | 0.8498 | 0.2797 | 0.2813 | Lower expr. |
| OV | SCARB1 | PFS | 0.8096 | 0.1073 | 0.1074 | Lower expr. |
| OV | SCARB1 | DSS | 0.8456 | 0.3046 | 0.3068 | Lower expr. |
| OV | SCARB1 | DFI | 0.6106 | 0.0139 | 0.0131 | Lower expr. |
| OV | TMPRSS2 | OS | 1.2010 | 0.2169 | 0.2159 | Higher expr. |
| OV | TMPRSS2 | PFS | 1.0284 | 0.8298 | 0.8290 | Higher expr. |
| OV | TMPRSS2 | DSS | 1.2050 | 0.2472 | 0.2461 | Higher expr. |
| OV | TMPRSS2 | DFI | 0.9867 | 0.9459 | 0.9504 | Lower expr. |
| PAAD | ACE2 | OS | 1.1268 | 0.5658 | 0.5666 | Higher expr. |
| PAAD | ACE2 | PFS | 1.1077 | 0.5730 | 0.5732 | Higher expr. |
| PAAD | ACE2 | DSS | 1.1138 | 0.6459 | 0.6467 | Higher expr. |
| PAAD | ACE2 | DFI | 0.8782 | 0.7563 | 0.7562 | Lower expr. |
| PAAD | AXL | OS | 1.4421 | 0.0836 | 0.0816 | Higher expr. |
| PAAD | AXL | PFS | 1.5847 | 0.0132 | 0.0124 | Higher expr. |
| PAAD | AXL | DSS | 1.4593 | 0.1138 | 0.1110 | Higher expr. |
| PAAD | AXL | DFI | 4.5160 | 0.0021 | 0.0009 | Higher expr. |
| PAAD | NRP1 | OS | 0.9528 | 0.8164 | 0.8165 | Lower expr. |
| PAAD | NRP1 | PFS | 1.0296 | 0.8730 | 0.8742 | Higher expr. |
| PAAD | NRP1 | DSS | 0.9595 | 0.8598 | 0.8607 | Lower expr. |
| PAAD | NRP1 | DFI | 1.7198 | 0.2235 | 0.2184 | Higher expr. |
| PAAD | SCARB1 | OS | 0.7218 | 0.1201 | 0.1185 | Lower expr. |
| PAAD | SCARB1 | PFS | 0.7756 | 0.1631 | 0.1626 | Lower expr. |
| PAAD | SCARB1 | DSS | 0.6781 | 0.1003 | 0.0981 | Lower expr. |
| PAAD | SCARB1 | DFI | 0.5132 | 0.1198 | 0.1132 | Lower expr. |
| PAAD | TMPRSS2 | OS | 1.2982 | 0.2146 | 0.2133 | Higher expr. |
| PAAD | TMPRSS2 | PFS | 1.1125 | 0.5594 | 0.5588 | Higher expr. |
| PAAD | TMPRSS2 | DSS | 1.2409 | 0.3606 | 0.3603 | Higher expr. |
| PAAD | TMPRSS2 | DFI | 1.1679 | 0.7138 | 0.7135 | Higher expr. |
| PCPG | ACE2 | OS | 0.6758 | 0.5977 | 0.5962 | Lower expr. |
| PCPG | ACE2 | PFS | 1.2464 | 0.5913 | 0.5894 | Higher expr. |
| PCPG | ACE2 | DSS | 0.2299 | 0.1837 | 0.1474 | Lower expr. |
| PCPG | ACE2 | DFI | 2.8280 | 0.3685 | 0.3472 | Higher expr. |
| PCPG | AXL | OS | 0.6487 | 0.5540 | 0.5485 | Lower expr. |
| PCPG | AXL | PFS | 1.0147 | 0.9716 | 0.9727 | Higher expr. |
| PCPG | AXL | DSS | 1.0826 | 0.9228 | 0.9246 | Higher expr. |
| PCPG | AXL | DFI | 0.3191 | 0.3226 | 0.2966 | Lower expr. |
| PCPG | NRP1 | OS | 0.2694 | 0.1185 | 0.0977 | Lower expr. |
| PCPG | NRP1 | PFS | 0.6409 | 0.2924 | 0.2899 | Lower expr. |
| PCPG | NRP1 | DSS | 0.1436 | 0.0854 | 0.0514 | Lower expr. |
| PCPG | NRP1 | DFI | 0.4159 | 0.4491 | 0.4349 | Lower expr. |
| PCPG | SCARB1 | OS | 0.5020 | 0.3583 | 0.3452 | Lower expr. |
| PCPG | SCARB1 | PFS | 0.8278 | 0.6451 | 0.6415 | Lower expr. |
| PCPG | SCARB1 | DSS | 0.7976 | 0.7877 | 0.7805 | Lower expr. |
| PCPG | SCARB1 | DFI | 2.9213 | 0.3535 | 0.3307 | Higher expr. |
| PCPG | TMPRSS2 | OS | 2.3978 | 0.2183 | 0.2075 | Higher expr. |
| PCPG | TMPRSS2 | PFS | 1.5318 | 0.3040 | 0.3031 | Higher expr. |
| PCPG | TMPRSS2 | DSS | 2.4462 | 0.2762 | 0.2653 | Higher expr. |
| PCPG | TMPRSS2 | DFI | 0.0000 | 0.9988 | 0.1297 | Lower expr. |
| PRAD | ACE2 | OS | 0.5690 | 0.3856 | 0.3796 | Lower expr. |
| PRAD | ACE2 | PFS | 1.0038 | 0.9851 | 0.9853 | Higher expr. |
| PRAD | ACE2 | DSS | 0.5104 | 0.4675 | 0.4598 | Lower expr. |
| PRAD | ACE2 | DFI | 0.9683 | 0.9286 | 0.9282 | Lower expr. |
| PRAD | AXL | OS | 0.7193 | 0.6128 | 0.6113 | Lower expr. |
| PRAD | AXL | PFS | 0.9635 | 0.8542 | 0.8540 | Lower expr. |
| PRAD | AXL | DSS | 0.2464 | 0.2111 | 0.1758 | Lower expr. |
| PRAD | AXL | DFI | 0.6534 | 0.2427 | 0.2391 | Lower expr. |
| PRAD | NRP1 | OS | 0.5443 | 0.3881 | 0.3814 | Lower expr. |
| PRAD | NRP1 | PFS | 2.1763 | 0.0003 | 0.0002 | Higher expr. |
| PRAD | NRP1 | DSS | 0.7674 | 0.7750 | 0.7744 | Lower expr. |
| PRAD | NRP1 | DFI | 4.2177 | 0.0008 | 0.0003 | Higher expr. |
| PRAD | SCARB1 | OS | 1.3852 | 0.6164 | 0.6149 | Higher expr. |
| PRAD | SCARB1 | PFS | 0.9173 | 0.6702 | 0.6701 | Lower expr. |
| PRAD | SCARB1 | DSS | 4.0293 | 0.2135 | 0.1784 | Higher expr. |
| PRAD | SCARB1 | DFI | 0.8686 | 0.6955 | 0.6948 | Lower expr. |
| PRAD | TMPRSS2 | OS | 0.6088 | 0.4839 | 0.4798 | Lower expr. |
| PRAD | TMPRSS2 | PFS | 0.7908 | 0.2515 | 0.2505 | Lower expr. |
| PRAD | TMPRSS2 | DSS | 0.3264 | 0.3252 | 0.3019 | Lower expr. |
| PRAD | TMPRSS2 | DFI | 0.6780 | 0.2860 | 0.2832 | Lower expr. |
| READ | ACE2 | OS | 0.7457 | 0.5371 | 0.5339 | Lower expr. |
| READ | ACE2 | PFS | 0.8348 | 0.6286 | 0.6271 | Lower expr. |
| READ | ACE2 | DSS | 0.1996 | 0.1519 | 0.1127 | Lower expr. |
| READ | ACE2 | DFI | 0.7128 | 0.7219 | 0.7209 | Lower expr. |
| READ | AXL | OS | 1.4131 | 0.4674 | 0.4746 | Higher expr. |
| READ | AXL | PFS | 1.5808 | 0.2257 | 0.2264 | Higher expr. |
| READ | AXL | DSS | 0.8073 | 0.8150 | 0.8147 | Lower expr. |
| READ | AXL | DFI | 2.2892 | 0.4706 | 0.4595 | Higher expr. |
| READ | NRP1 | OS | 3.1729 | 0.0260 | 0.0206 | Higher expr. |
| READ | NRP1 | PFS | 2.8414 | 0.0101 | 0.0076 | Higher expr. |
| READ | NRP1 | DSS | 4.8801 | 0.1568 | 0.1170 | Higher expr. |
| READ | NRP1 | DFI | 2.6797 | 0.3795 | 0.3607 | Higher expr. |
| READ | SCARB1 | OS | 0.4696 | 0.1360 | 0.1275 | Lower expr. |
| READ | SCARB1 | PFS | 0.5408 | 0.1061 | 0.1007 | Lower expr. |
| READ | SCARB1 | DSS | 0.6538 | 0.6435 | 0.6411 | Lower expr. |
| READ | SCARB1 | DFI | 0.5013 | 0.4527 | 0.4441 | Lower expr. |
| READ | TMPRSS2 | OS | 1.1206 | 0.8165 | 0.8050 | Higher expr. |
| READ | TMPRSS2 | PFS | 0.5893 | 0.1809 | 0.1791 | Lower expr. |
| READ | TMPRSS2 | DSS | 1.4079 | 0.7080 | 0.7066 | Higher expr. |
| READ | TMPRSS2 | DFI | 0.4607 | 0.4896 | 0.4789 | Lower expr. |
| SARC | ACE2 | OS | 0.8734 | 0.5016 | 0.5003 | Lower expr. |
| SARC | ACE2 | PFS | 0.8493 | 0.3071 | 0.3049 | Lower expr. |
| SARC | ACE2 | DSS | 1.0066 | 0.9762 | 0.9773 | Higher expr. |
| SARC | ACE2 | DFI | 0.9447 | 0.8138 | 0.8100 | Lower expr. |
| SARC | AXL | OS | 1.0070 | 0.9723 | 0.9735 | Higher expr. |
| SARC | AXL | PFS | 0.8832 | 0.4372 | 0.4361 | Lower expr. |
| SARC | AXL | DSS | 1.0685 | 0.7650 | 0.7662 | Higher expr. |
| SARC | AXL | DFI | 0.8955 | 0.6472 | 0.6468 | Lower expr. |
| SARC | NRP1 | OS | 0.9748 | 0.8992 | 0.9000 | Lower expr. |
| SARC | NRP1 | PFS | 1.0809 | 0.6267 | 0.6251 | Higher expr. |
| SARC | NRP1 | DSS | 0.9766 | 0.9146 | 0.9153 | Lower expr. |
| SARC | NRP1 | DFI | 1.0005 | 0.9982 | 0.9967 | Higher expr. |
| SARC | SCARB1 | OS | 0.9593 | 0.8369 | 0.8389 | Lower expr. |
| SARC | SCARB1 | PFS | 0.9396 | 0.6970 | 0.7001 | Lower expr. |
| SARC | SCARB1 | DSS | 0.8730 | 0.5431 | 0.5445 | Lower expr. |
| SARC | SCARB1 | DFI | 0.7576 | 0.2646 | 0.2661 | Lower expr. |
| SARC | TMPRSS2 | OS | 1.3823 | 0.1100 | 0.1085 | Higher expr. |
| SARC | TMPRSS2 | PFS | 1.2328 | 0.1909 | 0.1900 | Higher expr. |
| SARC | TMPRSS2 | DSS | 1.4527 | 0.0940 | 0.0921 | Higher expr. |
| SARC | TMPRSS2 | DFI | 1.0251 | 0.9196 | 0.9190 | Higher expr. |
| SKCM | ACE2 | OS | 1.1332 | 0.3539 | 0.3541 | Higher expr. |
| SKCM | ACE2 | PFS | 1.0191 | 0.8656 | 0.8667 | Higher expr. |
| SKCM | ACE2 | DSS | 1.1323 | 0.3955 | 0.3959 | Higher expr. |
| SKCM | AXL | OS | 0.5828 | 0.0001 | 0.0001 | Lower expr. |
| SKCM | AXL | PFS | 0.7374 | 0.0067 | 0.0065 | Lower expr. |
| SKCM | AXL | DSS | 0.5637 | 0.0001 | 0.0001 | Lower expr. |
| SKCM | NRP1 | OS | 0.9602 | 0.7637 | 0.7639 | Lower expr. |
| SKCM | NRP1 | PFS | 1.0272 | 0.8123 | 0.8127 | Higher expr. |
| SKCM | NRP1 | DSS | 1.0211 | 0.8869 | 0.8867 | Higher expr. |
| SKCM | SCARB1 | OS | 1.3742 | 0.0203 | 0.0198 | Higher expr. |
| SKCM | SCARB1 | PFS | 1.3317 | 0.0118 | 0.0116 | Higher expr. |
| SKCM | SCARB1 | DSS | 1.2609 | 0.1198 | 0.1191 | Higher expr. |
| SKCM | TMPRSS2 | OS | 1.1507 | 0.2974 | 0.2966 | Higher expr. |
| SKCM | TMPRSS2 | PFS | 0.9198 | 0.4534 | 0.4534 | Lower expr. |
| SKCM | TMPRSS2 | DSS | 1.1809 | 0.2554 | 0.2544 | Higher expr. |
| STAD | ACE2 | OS | 0.8977 | 0.4991 | 0.4994 | Lower expr. |
| STAD | ACE2 | PFS | 1.0312 | 0.8301 | 0.8295 | Higher expr. |
| STAD | ACE2 | DSS | 0.8471 | 0.4199 | 0.4196 | Lower expr. |
| STAD | ACE2 | DFI | 0.9521 | 0.8708 | 0.8708 | Lower expr. |
| STAD | AXL | OS | 1.4761 | 0.0157 | 0.0151 | Higher expr. |
| STAD | AXL | PFS | 1.4698 | 0.0077 | 0.0074 | Higher expr. |
| STAD | AXL | DSS | 1.4361 | 0.0796 | 0.0779 | Higher expr. |
| STAD | AXL | DFI | 1.8792 | 0.0418 | 0.0386 | Higher expr. |
| STAD | NRP1 | OS | 1.9004 | 0.0001 | 0.0001 | Higher expr. |
| STAD | NRP1 | PFS | 1.7430 | 0.0001 | 0.0001 | Higher expr. |
| STAD | NRP1 | DSS | 1.7720 | 0.0063 | 0.0057 | Higher expr. |
| STAD | NRP1 | DFI | 1.9094 | 0.0365 | 0.0335 | Higher expr. |
| STAD | SCARB1 | OS | 0.9887 | 0.9429 | 0.9425 | Lower expr. |
| STAD | SCARB1 | PFS | 0.9979 | 0.9880 | 0.9870 | Lower expr. |
| STAD | SCARB1 | DSS | 1.1172 | 0.5901 | 0.5896 | Higher expr. |
| STAD | SCARB1 | DFI | 1.1608 | 0.6217 | 0.6213 | Higher expr. |
| STAD | TMPRSS2 | OS | 0.8898 | 0.4637 | 0.4630 | Lower expr. |
| STAD | TMPRSS2 | PFS | 0.8731 | 0.3434 | 0.3425 | Lower expr. |
| STAD | TMPRSS2 | DSS | 0.8548 | 0.4462 | 0.4457 | Lower expr. |
| STAD | TMPRSS2 | DFI | 1.0719 | 0.8190 | 0.8183 | Higher expr. |
| TGCT | ACE2 | OS | 0.3346 | 0.3431 | 0.3192 | Lower expr. |
| TGCT | ACE2 | PFS | 1.0984 | 0.7662 | 0.7614 | Higher expr. |
| TGCT | ACE2 | DSS | 0.4942 | 0.5650 | 0.5569 | Lower expr. |
| TGCT | ACE2 | DFI | 1.3556 | 0.4033 | 0.3925 | Higher expr. |
| TGCT | AXL | OS | 5.87E+08 | 0.9987 | 0.0468 | Higher expr. |
| TGCT | AXL | PFS | 1.0944 | 0.7751 | 0.7671 | Higher expr. |
| TGCT | AXL | DSS | 5.95E+08 | 0.9989 | 0.0831 | Higher expr. |
| TGCT | AXL | DFI | 0.9674 | 0.9266 | 0.9431 | Lower expr. |
| TGCT | NRP1 | OS | 3.1462 | 0.3209 | 0.2948 | Higher expr. |
| TGCT | NRP1 | PFS | 1.0253 | 0.9363 | 0.9322 | Higher expr. |
| TGCT | NRP1 | DSS | 2.1677 | 0.5276 | 0.5173 | Higher expr. |
| TGCT | NRP1 | DFI | 0.8478 | 0.6436 | 0.6488 | Lower expr. |
| TGCT | SCARB1 | OS | 2.4220 | 0.4505 | 0.4369 | Higher expr. |
| TGCT | SCARB1 | PFS | 1.3269 | 0.3713 | 0.3636 | Higher expr. |
| TGCT | SCARB1 | DSS | 2.0416 | 0.5601 | 0.5517 | Higher expr. |
| TGCT | SCARB1 | DFI | 1.5195 | 0.2388 | 0.2276 | Higher expr. |
| TGCT | TMPRSS2 | OS | 2.2210 | 0.4952 | 0.4845 | Higher expr. |
| TGCT | TMPRSS2 | PFS | 1.5923 | 0.1585 | 0.1512 | Higher expr. |
| TGCT | TMPRSS2 | DSS | 1.8418 | 0.6181 | 0.6127 | Higher expr. |
| TGCT | TMPRSS2 | DFI | 1.5504 | 0.2294 | 0.2167 | Higher expr. |
| THCA | ACE2 | OS | 1.2770 | 0.6258 | 0.6249 | Higher expr. |
| THCA | ACE2 | PFS | 0.9988 | 0.9964 | 0.9965 | Lower expr. |
| THCA | ACE2 | DSS | 1.5727 | 0.5540 | 0.5506 | Higher expr. |
| THCA | ACE2 | DFI | 0.7369 | 0.4360 | 0.4338 | Lower expr. |
| THCA | AXL | OS | 2.0762 | 0.2088 | 0.1992 | Higher expr. |
| THCA | AXL | PFS | 1.3605 | 0.2371 | 0.2350 | Higher expr. |
| THCA | AXL | DSS | 4.6970 | 0.1531 | 0.1157 | Higher expr. |
| THCA | AXL | DFI | 1.3382 | 0.4573 | 0.4560 | Higher expr. |
| THCA | NRP1 | OS | 2.2316 | 0.1372 | 0.1270 | Higher expr. |
| THCA | NRP1 | PFS | 0.7786 | 0.3241 | 0.3231 | Lower expr. |
| THCA | NRP1 | DSS | 1.3786 | 0.6747 | 0.6734 | Higher expr. |
| THCA | NRP1 | DFI | 0.8191 | 0.6065 | 0.6054 | Lower expr. |
| THCA | SCARB1 | OS | 1.3802 | 0.5230 | 0.5212 | Higher expr. |
| THCA | SCARB1 | PFS | 0.9193 | 0.7388 | 0.7384 | Lower expr. |
| THCA | SCARB1 | DSS | 0.7901 | 0.7579 | 0.7573 | Lower expr. |
| THCA | SCARB1 | DFI | 0.6907 | 0.3449 | 0.3424 | Lower expr. |
| THCA | TMPRSS2 | OS | 0.6997 | 0.4799 | 0.4776 | Lower expr. |
| THCA | TMPRSS2 | PFS | 0.8297 | 0.4599 | 0.4594 | Lower expr. |
| THCA | TMPRSS2 | DSS | 0.6961 | 0.6353 | 0.6335 | Lower expr. |
| THCA | TMPRSS2 | DFI | 0.7889 | 0.5384 | 0.5370 | Lower expr. |
| THYM | ACE2 | OS | 1.6678 | 0.4714 | 0.4667 | Higher expr. |
| THYM | ACE2 | PFS | 1.0729 | 0.8636 | 0.8636 | Higher expr. |
| THYM | ACE2 | DSS | 2.3970 | 0.4524 | 0.4387 | Higher expr. |
| THYM | AXL | OS | 0.6990 | 0.6192 | 0.6175 | Lower expr. |
| THYM | AXL | PFS | 0.4471 | 0.0743 | 0.0669 | Lower expr. |
| THYM | AXL | DSS | 0.0000 | 0.9988 | 0.1128 | Lower expr. |
| THYM | NRP1 | OS | 0.8639 | 0.8277 | 0.8275 | Lower expr. |
| THYM | NRP1 | PFS | 0.7213 | 0.4305 | 0.4285 | Lower expr. |
| THYM | NRP1 | DSS | 0.0000 | 0.9987 | 0.0652 | Lower expr. |
| THYM | SCARB1 | OS | 2.4640 | 0.2366 | 0.2243 | Higher expr. |
| THYM | SCARB1 | PFS | 0.8105 | 0.6162 | 0.6156 | Lower expr. |
| THYM | SCARB1 | DSS | 3.7058 | 0.3236 | 0.3030 | Higher expr. |
| THYM | TMPRSS2 | OS | 0.1824 | 0.0427 | 0.0258 | Lower expr. |
| THYM | TMPRSS2 | PFS | 0.7324 | 0.4486 | 0.4468 | Lower expr. |
| THYM | TMPRSS2 | DSS | 0.1570 | 0.1357 | 0.1020 | Lower expr. |
| UCEC | ACE2 | OS | 0.9778 | 0.9497 | 0.9497 | Lower expr. |
| UCEC | ACE2 | PFS | 1.0818 | 0.7836 | 0.7835 | Higher expr. |
| UCEC | ACE2 | DSS | 1.1564 | 0.7311 | 0.7309 | Higher expr. |
| UCEC | ACE2 | DFI | 1.0594 | 0.9082 | 0.9082 | Higher expr. |
| UCEC | AXL | OS | 1.4578 | 0.2834 | 0.2806 | Higher expr. |
| UCEC | AXL | PFS | 0.8888 | 0.6797 | 0.6805 | Lower expr. |
| UCEC | AXL | DSS | 2.6017 | 0.0357 | 0.0294 | Higher expr. |
| UCEC | AXL | DFI | 0.4359 | 0.1242 | 0.1138 | Lower expr. |
| UCEC | NRP1 | OS | 1.6596 | 0.1575 | 0.1533 | Higher expr. |
| UCEC | NRP1 | PFS | 1.4386 | 0.2047 | 0.2022 | Higher expr. |
| UCEC | NRP1 | DSS | 2.4281 | 0.0515 | 0.0445 | Higher expr. |
| UCEC | NRP1 | DFI | 1.7051 | 0.3022 | 0.2965 | Higher expr. |
| UCEC | SCARB1 | OS | 1.6430 | 0.1607 | 0.1566 | Higher expr. |
| UCEC | SCARB1 | PFS | 1.5235 | 0.1416 | 0.1387 | Higher expr. |
| UCEC | SCARB1 | DSS | 1.3837 | 0.4374 | 0.4354 | Higher expr. |
| UCEC | SCARB1 | DFI | 1.9873 | 0.1838 | 0.1753 | Higher expr. |
| UCEC | TMPRSS2 | OS | 0.6661 | 0.2506 | 0.2475 | Lower expr. |
| UCEC | TMPRSS2 | PFS | 0.4660 | 0.0085 | 0.0071 | Lower expr. |
| UCEC | TMPRSS2 | DSS | 0.4266 | 0.0533 | 0.0467 | Lower expr. |
| UCEC | TMPRSS2 | DFI | 0.5328 | 0.2118 | 0.2044 | Lower expr. |
| UCS | ACE2 | OS | 0.5827 | 0.1194 | 0.1149 | Lower expr. |
| UCS | ACE2 | PFS | 0.4389 | 0.0106 | 0.0087 | Lower expr. |
| UCS | ACE2 | DSS | 0.4914 | 0.0584 | 0.0533 | Lower expr. |
| UCS | ACE2 | DFI | 0.2878 | 0.0647 | 0.0494 | Lower expr. |
| UCS | AXL | OS | 1.2392 | 0.5288 | 0.5300 | Higher expr. |
| UCS | AXL | PFS | 1.0228 | 0.9425 | 0.9391 | Higher expr. |
| UCS | AXL | DSS | 1.2275 | 0.5767 | 0.5784 | Higher expr. |
| UCS | AXL | DFI | 0.6366 | 0.5244 | 0.5209 | Lower expr. |
| UCS | NRP1 | OS | 1.2354 | 0.5426 | 0.5416 | Higher expr. |
| UCS | NRP1 | PFS | 1.5572 | 0.1705 | 0.1684 | Higher expr. |
| UCS | NRP1 | DSS | 1.1142 | 0.7655 | 0.7651 | Higher expr. |
| UCS | NRP1 | DFI | 1.6654 | 0.4289 | 0.4243 | Higher expr. |
| UCS | SCARB1 | OS | 1.1169 | 0.7523 | 0.7550 | Higher expr. |
| UCS | SCARB1 | PFS | 0.9688 | 0.9217 | 0.9176 | Lower expr. |
| UCS | SCARB1 | DSS | 1.2772 | 0.5062 | 0.5079 | Higher expr. |
| UCS | SCARB1 | DFI | 0.1642 | 0.0892 | 0.0529 | Lower expr. |
| UCS | TMPRSS2 | OS | 1.2601 | 0.5049 | 0.5036 | Higher expr. |
| UCS | TMPRSS2 | PFS | 1.7497 | 0.0858 | 0.0812 | Higher expr. |
| UCS | TMPRSS2 | DSS | 1.1226 | 0.7496 | 0.7489 | Higher expr. |
| UCS | TMPRSS2 | DFI | 5.5184 | 0.0320 | 0.0164 | Higher expr. |
| UVM | ACE2 | OS | 0.3336 | 0.0214 | 0.0157 | Lower expr. |
| UVM | ACE2 | PFS | 0.5175 | 0.0742 | 0.0698 | Lower expr. |
| UVM | ACE2 | DSS | 0.3460 | 0.0296 | 0.0230 | Lower expr. |
| UVM | AXL | OS | 1.1186 | 0.7889 | 0.7888 | Higher expr. |
| UVM | AXL | PFS | 1.2387 | 0.5411 | 0.5406 | Higher expr. |
| UVM | AXL | DSS | 1.0907 | 0.8428 | 0.8428 | Higher expr. |
| UVM | NRP1 | OS | 3.2419 | 0.0103 | 0.0068 | Higher expr. |
| UVM | NRP1 | PFS | 3.0584 | 0.0038 | 0.0025 | Higher expr. |
| UVM | NRP1 | DSS | 3.4176 | 0.0117 | 0.0075 | Higher expr. |
| UVM | SCARB1 | OS | 4.0115 | 0.0050 | 0.0025 | Higher expr. |
| UVM | SCARB1 | PFS | 2.6777 | 0.0111 | 0.0084 | Higher expr. |
| UVM | SCARB1 | DSS | 4.5939 | 0.0039 | 0.0016 | Higher expr. |
| UVM | TMPRSS2 | OS | 1.4063 | 0.4375 | 0.4353 | Higher expr. |
| UVM | TMPRSS2 | PFS | 1.5166 | 0.2512 | 0.2473 | Higher expr. |
| UVM | TMPRSS2 | DSS | 1.5675 | 0.3326 | 0.3285 | Higher expr. |

Table S4. SnvSummaryTable

| cancertype | symbol | EffectiveMut | NonEffectiveMut | sample_size | percentage |
| --- | --- | --- | --- | --- | --- |
| ACC | AXL | 2 | 0 | 92 | 2.1739 |
| ACC | SCARB1 | 1 | 0 | 92 | 1.0870 |
| ACC | TMPRSS2 | 0 | 1 | 92 | 0.0000 |
| BLCA | ACE2 | 4 | 5 | 411 | 0.9732 |
| BLCA | AXL | 5 | 2 | 411 | 1.2165 |
| BLCA | NRP1 | 10 | 4 | 411 | 2.4331 |
| BLCA | SCARB1 | 1 | 5 | 411 | 0.2433 |
| BLCA | TMPRSS2 | 7 | 2 | 411 | 1.7032 |
| BRCA | ACE2 | 3 | 2 | 1026 | 0.2924 |
| BRCA | AXL | 8 | 0 | 1026 | 0.7797 |
| BRCA | NRP1 | 6 | 6 | 1026 | 0.5848 |
| BRCA | SCARB1 | 8 | 0 | 1026 | 0.7797 |
| BRCA | TMPRSS2 | 2 | 1 | 1026 | 0.1949 |
| CESC | ACE2 | 3 | 0 | 291 | 1.0309 |
| CESC | AXL | 4 | 1 | 291 | 1.3746 |
| CESC | NRP1 | 6 | 6 | 291 | 2.0619 |
| CESC | SCARB1 | 2 | 5 | 291 | 0.6873 |
| CESC | TMPRSS2 | 3 | 1 | 291 | 1.0309 |
| COAD | ACE2 | 10 | 5 | 407 | 2.4570 |
| COAD | AXL | 14 | 5 | 407 | 3.4398 |
| COAD | NRP1 | 8 | 11 | 407 | 1.9656 |
| COAD | SCARB1 | 5 | 5 | 407 | 1.2285 |
| COAD | TMPRSS2 | 9 | 1 | 407 | 2.2113 |
| DLBC | ACE2 | 1 | 0 | 37 | 2.7027 |
| DLBC | AXL | 1 | 0 | 37 | 2.7027 |
| ESCA | ACE2 | 2 | 0 | 185 | 1.0811 |
| ESCA | AXL | 1 | 2 | 185 | 0.5405 |
| ESCA | NRP1 | 1 | 2 | 185 | 0.5405 |
| GBM | ACE2 | 4 | 0 | 403 | 0.9926 |
| GBM | AXL | 4 | 1 | 403 | 0.9926 |
| GBM | NRP1 | 2 | 2 | 403 | 0.4963 |
| GBM | SCARB1 | 1 | 3 | 403 | 0.2481 |
| GBM | TMPRSS2 | 2 | 0 | 403 | 0.4963 |
| HNSC | ACE2 | 4 | 2 | 509 | 0.7859 |
| HNSC | AXL | 6 | 2 | 509 | 1.1788 |
| HNSC | NRP1 | 6 | 6 | 509 | 1.1788 |
| HNSC | SCARB1 | 0 | 1 | 509 | 0.0000 |
| HNSC | TMPRSS2 | 3 | 2 | 509 | 0.5894 |
| KICH | SCARB1 | 0 | 1 | 66 | 0.0000 |
| KIRC | ACE2 | 1 | 0 | 370 | 0.2703 |
| KIRC | AXL | 3 | 1 | 370 | 0.8108 |
| KIRC | NRP1 | 2 | 1 | 370 | 0.5405 |
| KIRC | SCARB1 | 1 | 0 | 370 | 0.2703 |
| KIRC | TMPRSS2 | 0 | 1 | 370 | 0.0000 |
| KIRP | ACE2 | 1 | 1 | 282 | 0.3546 |
| KIRP | AXL | 1 | 0 | 282 | 0.3546 |
| KIRP | NRP1 | 0 | 2 | 282 | 0.0000 |
| KIRP | TMPRSS2 | 0 | 1 | 282 | 0.0000 |
| LGG | ACE2 | 1 | 2 | 526 | 0.1901 |
| LGG | AXL | 2 | 1 | 526 | 0.3802 |
| LGG | NRP1 | 2 | 1 | 526 | 0.3802 |
| LGG | SCARB1 | 0 | 1 | 526 | 0.0000 |
| LGG | TMPRSS2 | 0 | 1 | 526 | 0.0000 |
| LIHC | ACE2 | 1 | 0 | 365 | 0.2740 |
| LIHC | AXL | 0 | 2 | 365 | 0.0000 |
| LIHC | NRP1 | 2 | 1 | 365 | 0.5479 |
| LIHC | SCARB1 | 1 | 0 | 365 | 0.2740 |
| LIHC | TMPRSS2 | 1 | 1 | 365 | 0.2740 |
| LUAD | ACE2 | 9 | 3 | 567 | 1.5873 |
| LUAD | AXL | 6 | 4 | 567 | 1.0582 |
| LUAD | NRP1 | 9 | 7 | 567 | 1.5873 |
| LUAD | SCARB1 | 5 | 0 | 567 | 0.8818 |
| LUAD | TMPRSS2 | 3 | 4 | 567 | 0.5291 |
| LUSC | ACE2 | 7 | 1 | 485 | 1.4433 |
| LUSC | AXL | 5 | 4 | 485 | 1.0309 |
| LUSC | NRP1 | 6 | 2 | 485 | 1.2371 |
| LUSC | SCARB1 | 5 | 2 | 485 | 1.0309 |
| LUSC | TMPRSS2 | 0 | 3 | 485 | 0.0000 |
| MESO | AXL | 1 | 0 | 82 | 1.2195 |
| MESO | NRP1 | 2 | 0 | 82 | 2.4390 |
| OV | ACE2 | 1 | 2 | 412 | 0.2427 |
| OV | AXL | 4 | 1 | 412 | 0.9709 |
| OV | NRP1 | 3 | 5 | 412 | 0.7282 |
| OV | SCARB1 | 2 | 0 | 412 | 0.4854 |
| OV | TMPRSS2 | 3 | 2 | 412 | 0.7282 |
| PAAD | ACE2 | 2 | 0 | 178 | 1.1236 |
| PAAD | AXL | 2 | 1 | 178 | 1.1236 |
| PAAD | NRP1 | 0 | 2 | 178 | 0.0000 |
| PAAD | SCARB1 | 1 | 0 | 178 | 0.5618 |
| PCPG | SCARB1 | 1 | 0 | 184 | 0.5435 |
| PRAD | ACE2 | 1 | 0 | 498 | 0.2008 |
| PRAD | AXL | 3 | 2 | 498 | 0.6024 |
| PRAD | NRP1 | 1 | 1 | 498 | 0.2008 |
| PRAD | SCARB1 | 3 | 0 | 498 | 0.6024 |
| PRAD | TMPRSS2 | 4 | 2 | 498 | 0.8032 |
| READ | ACE2 | 3 | 1 | 149 | 2.0134 |
| READ | AXL | 1 | 0 | 149 | 0.6711 |
| READ | NRP1 | 2 | 2 | 149 | 1.3423 |
| READ | SCARB1 | 1 | 0 | 149 | 0.6711 |
| READ | TMPRSS2 | 2 | 1 | 149 | 1.3423 |
| SARC | ACE2 | 0 | 2 | 239 | 0.0000 |
| SARC | AXL | 4 | 1 | 239 | 1.6736 |
| SARC | NRP1 | 0 | 1 | 239 | 0.0000 |
| SARC | SCARB1 | 0 | 1 | 239 | 0.0000 |
| SARC | TMPRSS2 | 2 | 0 | 239 | 0.8368 |
| SKCM | ACE2 | 15 | 8 | 468 | 3.2051 |
| SKCM | AXL | 36 | 20 | 468 | 7.6923 |
| SKCM | NRP1 | 42 | 25 | 468 | 8.9744 |
| SKCM | SCARB1 | 8 | 13 | 468 | 1.7094 |
| SKCM | TMPRSS2 | 18 | 10 | 468 | 3.8462 |
| STAD | ACE2 | 11 | 4 | 439 | 2.5057 |
| STAD | AXL | 20 | 6 | 439 | 4.5558 |
| STAD | NRP1 | 15 | 10 | 439 | 3.4169 |
| STAD | SCARB1 | 5 | 2 | 439 | 1.1390 |
| STAD | TMPRSS2 | 3 | 2 | 439 | 0.6834 |
| THCA | AXL | 1 | 0 | 500 | 0.2000 |
| THCA | NRP1 | 2 | 0 | 500 | 0.4000 |
| THYM | AXL | 2 | 0 | 123 | 1.6260 |
| THYM | SCARB1 | 1 | 0 | 123 | 0.8130 |
| UCEC | ACE2 | 34 | 19 | 531 | 6.4030 |
| UCEC | AXL | 43 | 30 | 531 | 8.0979 |
| UCEC | NRP1 | 35 | 41 | 531 | 6.5913 |
| UCEC | SCARB1 | 18 | 9 | 531 | 3.3898 |
| UCEC | TMPRSS2 | 22 | 11 | 531 | 4.1431 |
| UCS | ACE2 | 2 | 0 | 57 | 3.5088 |
| UCS | AXL | 1 | 0 | 57 | 1.7544 |
| UVM | SCARB1 | 1 | 0 | 80 | 1.2500 |

Table S5. SnvAndSurvivalTable

| cancertype | symbol | sur_type | log_rank_p | cox_p | hr | higher_risk_of_death | Mutant | WT |
| --- | --- | --- | --- | --- | --- | --- | --- | --- |
| BLCA | ACE2 | OS | 0.6061 | 0.6079 | 0.6938 | WT | 4 | 405 |
| BLCA | ACE2 | PFS | 0.9501 | 0.9499 | 1.0373 | Mutant | 4 | 407 |
| BLCA | ACE2 | DSS | 0.9219 | 0.9216 | 0.9321 | WT | 4 | 349 |
| BLCA | AXL | OS | 0.6093 | 0.6108 | 1.4368 | Mutant | 5 | 404 |
| BLCA | AXL | PFS | 0.8860 | 0.8867 | 1.1067 | Mutant | 5 | 406 |
| BLCA | AXL | DSS | 0.3991 | 0.4051 | 1.8123 | Mutant | 5 | 348 |
| BLCA | NRP1 | OS | 0.3275 | 0.3317 | 1.5539 | Mutant | 10 | 399 |
| BLCA | NRP1 | PFS | 0.4880 | 0.4899 | 1.3309 | Mutant | 10 | 401 |
| BLCA | NRP1 | DSS | 0.5324 | 0.5387 | 0.5393 | WT | 6 | 347 |
| BLCA | NRP1 | DFI | 0.4409 | 0.9967 | 0.0000 | WT | 3 | 159 |
| BLCA | TMPRSS2 | OS | 0.9532 | 0.9535 | 0.9665 | WT | 7 | 402 |
| BLCA | TMPRSS2 | PFS | 0.4385 | 0.4417 | 0.6321 | WT | 7 | 404 |
| BLCA | TMPRSS2 | DSS | 0.8425 | 0.8433 | 0.8683 | WT | 6 | 347 |
| BLCA | TMPRSS2 | DFI | 0.2912 | 0.9972 | 0.0000 | WT | 4 | 158 |
| BRCA | ACE2 | OS | 0.6331 | 0.9951 | 0.0000 | WT | 3 | 1022 |
| BRCA | ACE2 | PFS | 0.5266 | 0.9936 | 0.0000 | WT | 3 | 1022 |
| BRCA | ACE2 | DSS | 0.6986 | 0.9961 | 0.0000 | WT | 3 | 956 |
| BRCA | ACE2 | DFI | 0.6282 | 0.9951 | 0.0000 | WT | 3 | 847 |
| BRCA | AXL | OS | 0.9068 | 0.9067 | 0.8890 | WT | 8 | 1017 |
| BRCA | AXL | PFS | 0.2491 | 0.2618 | 2.2252 | Mutant | 8 | 1017 |
| BRCA | AXL | DSS | 0.6632 | 0.6659 | 1.5453 | Mutant | 8 | 951 |
| BRCA | AXL | DFI | 0.0343 | 0.0509 | 4.0759 | Mutant | 8 | 842 |
| BRCA | NRP1 | OS | 0.2621 | 0.2851 | 2.9345 | Mutant | 6 | 1019 |
| BRCA | NRP1 | PFS | 0.6218 | 0.6247 | 1.6343 | Mutant | 6 | 1019 |
| BRCA | NRP1 | DSS | 0.1087 | 0.1427 | 4.4055 | Mutant | 6 | 953 |
| BRCA | NRP1 | DFI | 0.5612 | 0.9962 | 0.0000 | WT | 5 | 845 |
| BRCA | SCARB1 | OS | 0.3229 | 0.9935 | 0.0000 | WT | 8 | 1017 |
| BRCA | SCARB1 | PFS | 0.9660 | 0.9661 | 1.0436 | Mutant | 8 | 1017 |
| BRCA | SCARB1 | DSS | 0.4364 | 0.9949 | 0.0000 | WT | 8 | 951 |
| BRCA | SCARB1 | DFI | 0.4719 | 0.4811 | 2.0350 | Mutant | 7 | 843 |
| CESC | ACE2 | OS | 0.5066 | 0.9956 | 0.0000 | WT | 3 | 288 |
| CESC | ACE2 | PFS | 0.4643 | 0.9952 | 0.0000 | WT | 3 | 288 |
| CESC | ACE2 | DSS | 0.5457 | 0.9960 | 0.0000 | WT | 3 | 271 |
| CESC | AXL | OS | 0.9284 | 0.9282 | 0.9131 | WT | 4 | 287 |
| CESC | AXL | PFS | 0.6936 | 0.6954 | 0.6742 | WT | 4 | 287 |
| CESC | AXL | DSS | 0.9798 | 0.9800 | 1.0257 | Mutant | 4 | 270 |
| CESC | AXL | DFI | 0.4163 | 0.4292 | 2.2438 | Mutant | 3 | 157 |
| CESC | NRP1 | OS | 0.6149 | 0.6185 | 0.6052 | WT | 6 | 285 |
| CESC | NRP1 | PFS | 0.4480 | 0.4584 | 0.4740 | WT | 6 | 285 |
| CESC | NRP1 | DSS | 0.8578 | 0.8579 | 0.8345 | WT | 6 | 268 |
| CESC | NRP1 | DFI | 0.3645 | 0.9976 | 0.0000 | WT | 5 | 155 |
| CESC | TMPRSS2 | OS | 0.1762 | 0.2066 | 3.6181 | Mutant | 3 | 288 |
| CESC | TMPRSS2 | PFS | 0.4878 | 0.4965 | 1.9881 | Mutant | 3 | 288 |
| CESC | TMPRSS2 | DSS | 0.1557 | 0.1878 | 3.8382 | Mutant | 3 | 271 |
| COAD | ACE2 | OS | 0.6914 | 0.6931 | 1.3273 | Mutant | 10 | 394 |
| COAD | ACE2 | PFS | 0.6711 | 0.6730 | 0.7401 | WT | 10 | 394 |
| COAD | ACE2 | DSS | 0.4379 | 0.4447 | 1.7363 | Mutant | 10 | 358 |
| COAD | ACE2 | DFI | 0.4909 | 0.9971 | 0.0000 | WT | 4 | 154 |
| COAD | AXL | OS | 0.9573 | 0.9567 | 0.9686 | WT | 14 | 390 |
| COAD | AXL | PFS | 0.5428 | 0.5437 | 0.7347 | WT | 14 | 390 |
| COAD | AXL | DSS | 0.4680 | 0.4773 | 0.4881 | WT | 12 | 356 |
| COAD | AXL | DFI | 0.3251 | 0.9974 | 0.0000 | WT | 7 | 151 |
| COAD | NRP1 | OS | 0.3474 | 0.3646 | 0.4016 | WT | 8 | 396 |
| COAD | NRP1 | PFS | 0.3619 | 0.3698 | 0.5277 | WT | 8 | 396 |
| COAD | NRP1 | DSS | 0.6161 | 0.6210 | 0.6069 | WT | 8 | 360 |
| COAD | NRP1 | DFI | 0.8088 | 0.8094 | 1.2838 | Mutant | 4 | 154 |
| COAD | SCARB1 | OS | 0.3791 | 0.9942 | 0.0000 | WT | 5 | 399 |
| COAD | SCARB1 | PFS | 0.8078 | 0.8078 | 0.7831 | WT | 5 | 399 |
| COAD | SCARB1 | DSS | 0.4523 | 0.9951 | 0.0000 | WT | 5 | 363 |
| COAD | TMPRSS2 | OS | 0.1147 | 0.1238 | 2.2009 | Mutant | 9 | 395 |
| COAD | TMPRSS2 | PFS | 0.2387 | 0.2448 | 1.8055 | Mutant | 9 | 395 |
| COAD | TMPRSS2 | DSS | 0.2032 | 0.2168 | 2.4366 | Mutant | 7 | 361 |
| GBM | ACE2 | OS | 0.6473 | 0.6507 | 0.7251 | WT | 4 | 393 |
| GBM | ACE2 | PFS | 0.1853 | 0.2014 | 0.4040 | WT | 4 | 396 |
| GBM | ACE2 | DSS | 0.9866 | 0.9923 | 0.9903 | WT | 3 | 355 |
| GBM | AXL | OS | 0.4298 | 0.4410 | 0.4620 | WT | 4 | 393 |
| GBM | AXL | PFS | 0.4487 | 0.4538 | 0.5877 | WT | 4 | 396 |
| GBM | AXL | DSS | 0.4704 | 0.4794 | 0.4921 | WT | 4 | 354 |
| HNSC | ACE2 | OS | 0.8945 | 0.8944 | 0.8754 | WT | 4 | 504 |
| HNSC | ACE2 | PFS | 0.5632 | 0.5688 | 0.5649 | WT | 4 | 505 |
| HNSC | ACE2 | DSS | 0.8515 | 0.8519 | 1.2064 | Mutant | 4 | 415 |
| HNSC | AXL | OS | 0.7650 | 0.7650 | 1.2369 | Mutant | 6 | 502 |
| HNSC | AXL | PFS | 0.6083 | 0.6095 | 1.3457 | Mutant | 6 | 503 |
| HNSC | AXL | DSS | 0.3149 | 0.9934 | 0.0000 | WT | 4 | 415 |
| HNSC | AXL | DFI | 0.3868 | 0.9977 | 0.0000 | WT | 3 | 112 |
| HNSC | NRP1 | OS | 0.7511 | 0.7514 | 0.7984 | WT | 6 | 502 |
| HNSC | NRP1 | PFS | 0.4458 | 0.4490 | 1.4652 | Mutant | 6 | 503 |
| HNSC | NRP1 | DSS | 0.8618 | 0.8621 | 1.1319 | Mutant | 6 | 413 |
| HNSC | TMPRSS2 | OS | 0.8121 | 0.8117 | 1.1847 | Mutant | 3 | 505 |
| HNSC | TMPRSS2 | PFS | 0.8615 | 0.8621 | 0.8839 | WT | 3 | 506 |
| KIRC | AXL | OS | 0.3551 | 0.9939 | 0.0000 | WT | 3 | 367 |
| KIRC | AXL | PFS | 0.9801 | 0.9800 | 0.9751 | WT | 3 | 365 |
| KIRC | AXL | DSS | 0.4204 | 0.9966 | 0.0000 | WT | 3 | 329 |
| LUAD | ACE2 | OS | 0.4337 | 0.4394 | 0.5769 | WT | 9 | 499 |
| LUAD | ACE2 | PFS | 0.5029 | 0.5046 | 0.7143 | WT | 9 | 499 |
| LUAD | ACE2 | DSS | 0.2457 | 0.9951 | 0.0000 | WT | 7 | 432 |
| LUAD | ACE2 | DFI | 0.8243 | 0.8244 | 0.7999 | WT | 4 | 273 |
| LUAD | AXL | OS | 0.0117 | 0.0196 | 3.9176 | Mutant | 4 | 504 |
| LUAD | AXL | PFS | 0.2291 | 0.2387 | 1.9846 | Mutant | 4 | 504 |
| LUAD | NRP1 | OS | 0.7580 | 0.7584 | 0.8033 | WT | 7 | 501 |
| LUAD | NRP1 | PFS | 0.8466 | 0.8471 | 0.8940 | WT | 7 | 501 |
| LUAD | NRP1 | DSS | 0.8386 | 0.8388 | 1.1564 | Mutant | 7 | 432 |
| LUAD | NRP1 | DFI | 0.8574 | 0.8587 | 0.8359 | WT | 5 | 272 |
| LUAD | SCARB1 | OS | 0.1282 | 0.9936 | 0.0000 | WT | 5 | 503 |
| LUAD | SCARB1 | PFS | 0.2376 | 0.2459 | 1.9653 | Mutant | 5 | 503 |
| LUAD | SCARB1 | DSS | 0.1887 | 0.9944 | 0.0000 | WT | 5 | 434 |
| LUSC | ACE2 | OS | 0.3573 | 0.3618 | 1.5858 | Mutant | 7 | 472 |
| LUSC | ACE2 | PFS | 0.7653 | 0.7661 | 1.1620 | Mutant | 7 | 473 |
| LUSC | ACE2 | DSS | 0.9362 | 0.9360 | 0.9223 | WT | 4 | 353 |
| LUSC | AXL | OS | 0.4480 | 0.4533 | 0.5866 | WT | 5 | 474 |
| LUSC | AXL | PFS | 0.2953 | 0.3059 | 0.4831 | WT | 5 | 475 |
| LUSC | AXL | DSS | 0.5913 | 0.5956 | 0.5858 | WT | 4 | 353 |
| LUSC | NRP1 | OS | 0.6972 | 0.6974 | 1.2546 | Mutant | 6 | 473 |
| LUSC | NRP1 | PFS | 0.1834 | 0.1913 | 1.9350 | Mutant | 6 | 474 |
| LUSC | NRP1 | DSS | 0.3411 | 0.9960 | 0.0000 | WT | 3 | 354 |
| LUSC | NRP1 | DFI | 0.8292 | 0.8296 | 1.2439 | Mutant | 3 | 239 |
| LUSC | SCARB1 | OS | 0.9485 | 0.9487 | 1.0470 | Mutant | 4 | 475 |
| LUSC | SCARB1 | PFS | 0.4219 | 0.4264 | 1.5884 | Mutant | 4 | 476 |
| OV | AXL | OS | 0.9556 | 0.9563 | 1.0324 | Mutant | 4 | 404 |
| OV | AXL | PFS | 0.9165 | 0.9165 | 0.9483 | WT | 4 | 404 |
| OV | AXL | DSS | 0.3546 | 0.3635 | 1.9105 | Mutant | 3 | 373 |
| OV | NRP1 | OS | 0.3476 | 0.3560 | 0.5179 | WT | 3 | 405 |
| OV | NRP1 | PFS | 0.2400 | 0.2528 | 0.4433 | WT | 3 | 405 |
| OV | TMPRSS2 | OS | 0.4462 | 0.4565 | 0.4737 | WT | 3 | 405 |
| OV | TMPRSS2 | PFS | 0.3409 | 0.3495 | 0.5139 | WT | 3 | 405 |
| PRAD | AXL | OS | 0.8225 | 0.9985 | 0.0000 | WT | 3 | 495 |
| PRAD | AXL | PFS | 0.4642 | 0.4739 | 2.0575 | Mutant | 3 | 495 |
| PRAD | AXL | DSS | 0.8604 | 0.9988 | 0.0000 | WT | 3 | 490 |
| PRAD | SCARB1 | OS | 0.8283 | 0.9986 | 0.0000 | WT | 3 | 495 |
| PRAD | SCARB1 | PFS | 0.4376 | 0.4487 | 2.1453 | Mutant | 3 | 495 |
| PRAD | SCARB1 | DSS | 0.8955 | 0.9991 | 0.0000 | WT | 3 | 490 |
| PRAD | SCARB1 | DFI | 0.6121 | 0.9967 | 0.0000 | WT | 3 | 332 |
| PRAD | TMPRSS2 | OS | 0.8086 | 0.9984 | 0.0000 | WT | 4 | 494 |
| PRAD | TMPRSS2 | PFS | 0.3669 | 0.9941 | 0.0000 | WT | 4 | 494 |
| PRAD | TMPRSS2 | DSS | 0.8555 | 0.9988 | 0.0000 | WT | 4 | 489 |
| READ | ACE2 | OS | 0.5210 | 0.9973 | 0.0000 | WT | 3 | 145 |
| READ | ACE2 | PFS | 0.3310 | 0.9974 | 0.0000 | WT | 3 | 145 |
| READ | ACE2 | DSS | 0.6081 | 0.9978 | 0.0000 | WT | 3 | 133 |
| READ | ACE2 | DFI | 0.5544 | 0.9990 | 0.0000 | WT | 3 | 38 |
| SARC | AXL | OS | 0.5077 | 0.5151 | 0.5193 | WT | 4 | 235 |
| SARC | AXL | PFS | 0.9435 | 0.9432 | 1.0425 | Mutant | 4 | 235 |
| SARC | AXL | DSS | 0.6269 | 0.6302 | 0.6154 | WT | 4 | 219 |
| SKCM | ACE2 | OS | 0.4257 | 0.4274 | 0.7370 | WT | 15 | 444 |
| SKCM | ACE2 | PFS | 0.6509 | 0.6507 | 0.8643 | WT | 13 | 441 |
| SKCM | ACE2 | DSS | 0.3597 | 0.3638 | 0.6317 | WT | 12 | 415 |
| SKCM | AXL | OS | 0.7297 | 0.7293 | 0.9180 | WT | 36 | 423 |
| SKCM | AXL | PFS | 0.3544 | 0.3550 | 0.8242 | WT | 36 | 418 |
| SKCM | AXL | DSS | 0.5022 | 0.5026 | 0.8180 | WT | 30 | 397 |
| SKCM | NRP1 | OS | 0.0054 | 0.0059 | 1.7713 | Mutant | 44 | 415 |
| SKCM | NRP1 | PFS | 0.3137 | 0.3149 | 1.2071 | Mutant | 43 | 411 |
| SKCM | NRP1 | DSS | 0.0120 | 0.0130 | 1.7489 | Mutant | 40 | 387 |
| SKCM | SCARB1 | OS | 0.1183 | 0.1260 | 2.0028 | Mutant | 8 | 451 |
| SKCM | SCARB1 | PFS | 0.2832 | 0.2872 | 1.5037 | Mutant | 8 | 446 |
| SKCM | SCARB1 | DSS | 0.2184 | 0.2281 | 2.0204 | Mutant | 6 | 421 |
| SKCM | TMPRSS2 | OS | 0.0926 | 0.1021 | 0.4379 | WT | 18 | 441 |
| SKCM | TMPRSS2 | PFS | 0.5190 | 0.5198 | 0.8129 | WT | 18 | 436 |
| SKCM | TMPRSS2 | DSS | 0.0648 | 0.0771 | 0.3571 | WT | 17 | 410 |
| STAD | ACE2 | OS | 0.2512 | 0.2597 | 0.5182 | WT | 11 | 421 |
| STAD | ACE2 | PFS | 0.6442 | 0.6447 | 0.8113 | WT | 11 | 424 |
| STAD | ACE2 | DSS | 0.4634 | 0.4682 | 0.5953 | WT | 10 | 354 |
| STAD | ACE2 | DFI | 0.7859 | 0.7864 | 0.7601 | WT | 6 | 217 |
| STAD | AXL | OS | 0.2044 | 0.2108 | 0.5664 | WT | 20 | 412 |
| STAD | AXL | PFS | 0.0490 | 0.0563 | 0.4213 | WT | 20 | 415 |
| STAD | AXL | DSS | 0.1762 | 0.1919 | 0.3936 | WT | 17 | 347 |
| STAD | AXL | DFI | 0.1225 | 0.9959 | 0.0000 | WT | 11 | 212 |
| STAD | NRP1 | OS | 0.5286 | 0.5307 | 0.7281 | WT | 15 | 417 |
| STAD | NRP1 | PFS | 0.3584 | 0.3615 | 0.6615 | WT | 15 | 420 |
| STAD | NRP1 | DSS | 0.4508 | 0.4560 | 0.5870 | WT | 13 | 351 |
| STAD | NRP1 | DFI | 0.7775 | 0.7781 | 1.2263 | Mutant | 8 | 215 |
| STAD | SCARB1 | OS | 0.2967 | 0.3168 | 0.3656 | WT | 4 | 428 |
| STAD | SCARB1 | PFS | 0.1958 | 0.2237 | 0.2948 | WT | 4 | 431 |
| STAD | SCARB1 | DSS | 0.1782 | 0.9943 | 0.0000 | WT | 3 | 361 |
| STAD | TMPRSS2 | OS | 0.7386 | 0.7397 | 0.7149 | WT | 3 | 429 |
| STAD | TMPRSS2 | PFS | 0.5737 | 0.5786 | 0.5717 | WT | 3 | 432 |
| STAD | TMPRSS2 | DSS | 0.9079 | 0.9081 | 1.1243 | Mutant | 3 | 361 |
| UCEC | ACE2 | OS | 0.0188 | 0.0326 | 0.2159 | WT | 33 | 497 |
| UCEC | ACE2 | PFS | 0.0007 | 0.0042 | 0.1297 | WT | 33 | 497 |
| UCEC | ACE2 | DSS | 0.0497 | 0.0830 | 0.1737 | WT | 32 | 469 |
| UCEC | ACE2 | DFI | 0.0151 | 0.9960 | 0.0000 | WT | 28 | 363 |
| UCEC | AXL | OS | 0.0660 | 0.0757 | 0.4021 | WT | 43 | 487 |
| UCEC | AXL | PFS | 0.0195 | 0.0237 | 0.4153 | WT | 43 | 487 |
| UCEC | AXL | DSS | 0.0321 | 0.0630 | 0.1532 | WT | 40 | 461 |
| UCEC | AXL | DFI | 0.1203 | 0.1383 | 0.3436 | WT | 32 | 359 |
| UCEC | NRP1 | OS | 0.1323 | 0.1443 | 0.4239 | WT | 35 | 495 |
| UCEC | NRP1 | PFS | 0.2159 | 0.2201 | 0.6215 | WT | 35 | 495 |
| UCEC | NRP1 | DSS | 0.1915 | 0.2068 | 0.4029 | WT | 34 | 467 |
| UCEC | NRP1 | DFI | 0.2446 | 0.2577 | 0.4423 | WT | 27 | 364 |
| UCEC | SCARB1 | OS | 0.9899 | 0.9898 | 0.9934 | WT | 18 | 512 |
| UCEC | SCARB1 | PFS | 0.2544 | 0.2607 | 0.5649 | WT | 18 | 512 |
| UCEC | SCARB1 | DSS | 0.7301 | 0.7307 | 0.7804 | WT | 16 | 485 |
| UCEC | SCARB1 | DFI | 0.3802 | 0.3947 | 0.4235 | WT | 12 | 379 |
| UCEC | TMPRSS2 | OS | 0.0244 | 0.9941 | 0.0000 | WT | 22 | 508 |
| UCEC | TMPRSS2 | PFS | 0.0349 | 0.0512 | 0.2489 | WT | 22 | 508 |
| UCEC | TMPRSS2 | DSS | 0.0685 | 0.9952 | 0.0000 | WT | 22 | 479 |
| UCEC | TMPRSS2 | DFI | 0.2481 | 0.2722 | 0.3301 | WT | 18 | 373 |

Table S6. CnvSummaryTable

| cancertype | symbol | a_total | d_total | a_hete | d_hete | a_homo | d_homo |
| --- | --- | --- | --- | --- | --- | --- | --- |
| ACC | ACE2 | 50.0000 | 14.4444 | 50.0000 | 14.4444 | 0.0000 | 0.0000 |
| ACC | AXL | 58.8889 | 7.7778 | 57.7778 | 7.7778 | 1.1111 | 0.0000 |
| ACC | NRP1 | 27.7778 | 11.1111 | 27.7778 | 11.1111 | 0.0000 | 0.0000 |
| ACC | SCARB1 | 71.1111 | 7.7778 | 68.8889 | 5.5556 | 2.2222 | 2.2222 |
| ACC | TMPRSS2 | 36.6667 | 16.6667 | 36.6667 | 15.5556 | 0.0000 | 1.1111 |
| BLCA | ACE2 | 19.1176 | 18.6275 | 18.3824 | 18.1373 | 0.7353 | 0.4902 |
| BLCA | AXL | 38.2353 | 12.9902 | 36.5196 | 12.9902 | 1.7157 | 0.0000 |
| BLCA | NRP1 | 31.1275 | 16.9118 | 29.6569 | 16.1765 | 1.4706 | 0.7353 |
| BLCA | SCARB1 | 23.7745 | 17.8922 | 23.0392 | 17.6471 | 0.7353 | 0.2451 |
| BLCA | TMPRSS2 | 30.3922 | 21.8137 | 30.1471 | 21.3235 | 0.2451 | 0.4902 |
| BRCA | ACE2 | 17.0370 | 18.3333 | 16.1111 | 17.7778 | 0.9259 | 0.5556 |
| BRCA | AXL | 21.9444 | 17.3148 | 20.4630 | 16.9444 | 1.4815 | 0.3704 |
| BRCA | NRP1 | 21.8519 | 13.2407 | 20.7407 | 13.2407 | 1.1111 | 0.0000 |
| BRCA | SCARB1 | 17.6852 | 20.0000 | 16.9444 | 19.8148 | 0.7407 | 0.1852 |
| BRCA | TMPRSS2 | 21.3889 | 16.8519 | 19.9074 | 16.7593 | 1.4815 | 0.0926 |
| CESC | ACE2 | 17.6271 | 21.3559 | 15.5932 | 21.0169 | 2.0339 | 0.3390 |
| CESC | AXL | 28.8136 | 10.1695 | 27.1186 | 10.1695 | 1.6949 | 0.0000 |
| CESC | NRP1 | 9.4915 | 21.0169 | 9.1525 | 21.0169 | 0.3390 | 0.0000 |
| CESC | SCARB1 | 15.9322 | 7.7966 | 15.9322 | 7.7966 | 0.0000 | 0.0000 |
| CESC | TMPRSS2 | 15.2542 | 17.2881 | 14.2373 | 16.9492 | 1.0169 | 0.3390 |
| CHOL | ACE2 | 2.7778 | 44.4444 | 2.7778 | 44.4444 | 0.0000 | 0.0000 |
| CHOL | AXL | 19.4444 | 8.3333 | 16.6667 | 8.3333 | 2.7778 | 0.0000 |
| CHOL | NRP1 | 13.8889 | 11.1111 | 13.8889 | 11.1111 | 0.0000 | 0.0000 |
| CHOL | SCARB1 | 22.2222 | 19.4444 | 22.2222 | 19.4444 | 0.0000 | 0.0000 |
| CHOL | TMPRSS2 | 13.8889 | 38.8889 | 13.8889 | 38.8889 | 0.0000 | 0.0000 |
| COAD | ACE2 | 17.2949 | 15.5211 | 17.0732 | 14.6341 | 0.2217 | 0.8869 |
| COAD | AXL | 19.9557 | 8.4257 | 19.7339 | 8.4257 | 0.2217 | 0.0000 |
| COAD | NRP1 | 8.8692 | 14.6341 | 8.6475 | 14.6341 | 0.2217 | 0.0000 |
| COAD | SCARB1 | 19.7339 | 12.4169 | 19.7339 | 12.4169 | 0.0000 | 0.0000 |
| COAD | TMPRSS2 | 5.7650 | 28.1596 | 5.7650 | 27.9379 | 0.0000 | 0.2217 |
| DLBC | ACE2 | 6.2500 | 20.8333 | 6.2500 | 16.6667 | 0.0000 | 4.1667 |
| DLBC | AXL | 10.4167 | 0.0000 | 10.4167 | 0.0000 | 0.0000 | 0.0000 |
| DLBC | NRP1 | 10.4167 | 4.1667 | 10.4167 | 4.1667 | 0.0000 | 0.0000 |
| DLBC | SCARB1 | 18.7500 | 4.1667 | 18.7500 | 4.1667 | 0.0000 | 0.0000 |
| DLBC | TMPRSS2 | 20.8333 | 2.0833 | 20.8333 | 2.0833 | 0.0000 | 0.0000 |
| ESCA | ACE2 | 14.6739 | 35.3261 | 14.1304 | 32.6087 | 0.5435 | 2.7174 |
| ESCA | AXL | 22.8261 | 27.1739 | 22.2826 | 26.6304 | 0.5435 | 0.5435 |
| ESCA | NRP1 | 27.1739 | 22.2826 | 25.0000 | 22.2826 | 2.1739 | 0.0000 |
| ESCA | SCARB1 | 21.7391 | 25.5435 | 20.6522 | 25.5435 | 1.0870 | 0.0000 |
| ESCA | TMPRSS2 | 9.2391 | 60.3261 | 8.6957 | 59.2391 | 0.5435 | 1.0870 |
| GBM | ACE2 | 5.3726 | 19.5841 | 5.1993 | 19.0641 | 0.1733 | 0.5199 |
| GBM | AXL | 31.5425 | 11.7851 | 31.5425 | 11.7851 | 0.0000 | 0.0000 |
| GBM | NRP1 | 1.3865 | 82.8423 | 1.3865 | 82.8423 | 0.0000 | 0.0000 |
| GBM | SCARB1 | 9.0121 | 10.0520 | 8.1456 | 9.7054 | 0.8666 | 0.3466 |
| GBM | TMPRSS2 | 11.0919 | 8.6655 | 11.0919 | 8.6655 | 0.0000 | 0.0000 |
| HNSC | ACE2 | 9.1954 | 29.1188 | 8.8123 | 27.0115 | 0.3831 | 2.1073 |
| HNSC | AXL | 14.1762 | 18.9655 | 13.7931 | 18.9655 | 0.3831 | 0.0000 |
| HNSC | NRP1 | 9.9617 | 29.5019 | 9.1954 | 29.3103 | 0.7663 | 0.1916 |
| HNSC | SCARB1 | 14.9425 | 11.6858 | 14.3678 | 11.4943 | 0.5747 | 0.1916 |
| HNSC | TMPRSS2 | 7.2797 | 39.6552 | 7.0881 | 38.5057 | 0.1916 | 1.1494 |
| KICH | ACE2 | 12.1212 | 59.0909 | 12.1212 | 59.0909 | 0.0000 | 0.0000 |
| KICH | AXL | 27.2727 | 6.0606 | 27.2727 | 6.0606 | 0.0000 | 0.0000 |
| KICH | NRP1 | 6.0606 | 72.7273 | 6.0606 | 72.7273 | 0.0000 | 0.0000 |
| KICH | SCARB1 | 31.8182 | 3.0303 | 31.8182 | 3.0303 | 0.0000 | 0.0000 |
| KICH | TMPRSS2 | 6.0606 | 53.0303 | 6.0606 | 53.0303 | 0.0000 | 0.0000 |
| KIRC | ACE2 | 5.3030 | 9.8485 | 5.3030 | 9.4697 | 0.0000 | 0.3788 |
| KIRC | AXL | 10.7955 | 1.3258 | 10.6061 | 1.3258 | 0.1894 | 0.0000 |
| KIRC | NRP1 | 2.8409 | 12.5000 | 2.8409 | 12.5000 | 0.0000 | 0.0000 |
| KIRC | SCARB1 | 23.1061 | 1.1364 | 22.9167 | 1.1364 | 0.1894 | 0.0000 |
| KIRC | TMPRSS2 | 9.8485 | 10.2273 | 9.6591 | 10.2273 | 0.1894 | 0.0000 |
| KIRP | ACE2 | 31.2500 | 11.4583 | 31.2500 | 11.1111 | 0.0000 | 0.3472 |
| KIRP | AXL | 1.3889 | 5.9028 | 1.3889 | 5.5556 | 0.0000 | 0.3472 |
| KIRP | NRP1 | 3.1250 | 5.5556 | 2.7778 | 5.5556 | 0.3472 | 0.0000 |
| KIRP | SCARB1 | 39.9306 | 0.0000 | 39.5833 | 0.0000 | 0.3472 | 0.0000 |
| KIRP | TMPRSS2 | 6.5972 | 14.5833 | 6.5972 | 14.5833 | 0.0000 | 0.0000 |
| LAML | ACE2 | 1.5707 | 2.6178 | 1.5707 | 2.6178 | 0.0000 | 0.0000 |
| LAML | AXL | 3.1414 | 1.5707 | 3.1414 | 1.5707 | 0.0000 | 0.0000 |
| LAML | NRP1 | 1.0471 | 0.5236 | 1.0471 | 0.5236 | 0.0000 | 0.0000 |
| LAML | SCARB1 | 0.5236 | 1.5707 | 0.0000 | 1.0471 | 0.5236 | 0.5236 |
| LAML | TMPRSS2 | 6.8063 | 0.5236 | 3.6649 | 0.5236 | 3.1414 | 0.0000 |
| LGG | ACE2 | 5.0682 | 20.4678 | 4.4834 | 19.1033 | 0.5848 | 1.3645 |
| LGG | AXL | 5.2632 | 45.6140 | 4.4834 | 43.8596 | 0.7797 | 1.7544 |
| LGG | NRP1 | 9.9415 | 16.1793 | 8.9669 | 16.1793 | 0.9747 | 0.0000 |
| LGG | SCARB1 | 4.2885 | 10.5263 | 2.9240 | 9.9415 | 1.3645 | 0.5848 |
| LGG | TMPRSS2 | 4.8733 | 7.4074 | 4.6784 | 7.4074 | 0.1949 | 0.0000 |
| LIHC | ACE2 | 9.7297 | 29.7297 | 9.4595 | 28.6486 | 0.2703 | 1.0811 |
| LIHC | AXL | 20.0000 | 10.5405 | 19.4595 | 10.5405 | 0.5405 | 0.0000 |
| LIHC | NRP1 | 19.1892 | 8.6486 | 17.2973 | 8.6486 | 1.8919 | 0.0000 |
| LIHC | SCARB1 | 12.4324 | 15.1351 | 11.6216 | 15.1351 | 0.8108 | 0.0000 |
| LIHC | TMPRSS2 | 8.3784 | 30.5405 | 8.3784 | 30.2703 | 0.0000 | 0.2703 |
| LUAD | ACE2 | 10.2713 | 26.5504 | 10.0775 | 25.5814 | 0.1938 | 0.9690 |
| LUAD | AXL | 22.2868 | 28.2946 | 20.7364 | 28.2946 | 1.5504 | 0.0000 |
| LUAD | NRP1 | 26.9380 | 17.2481 | 25.7752 | 17.2481 | 1.1628 | 0.0000 |
| LUAD | SCARB1 | 25.0000 | 23.4496 | 24.6124 | 22.8682 | 0.3876 | 0.5814 |
| LUAD | TMPRSS2 | 19.5736 | 34.3023 | 18.9922 | 34.1085 | 0.5814 | 0.1938 |
| LUSC | ACE2 | 15.3693 | 28.9421 | 14.1717 | 27.3453 | 1.1976 | 1.5968 |
| LUSC | AXL | 36.7265 | 23.3533 | 33.7325 | 22.9541 | 2.9940 | 0.3992 |
| LUSC | NRP1 | 19.5609 | 38.5230 | 18.3633 | 38.5230 | 1.1976 | 0.0000 |
| LUSC | SCARB1 | 24.9501 | 17.5649 | 24.9501 | 17.3653 | 0.0000 | 0.1996 |
| LUSC | TMPRSS2 | 11.1776 | 56.0878 | 10.7784 | 54.8902 | 0.3992 | 1.1976 |
| MESO | ACE2 | 5.7471 | 31.0345 | 5.7471 | 31.0345 | 0.0000 | 0.0000 |
| MESO | AXL | 12.6437 | 11.4943 | 10.3448 | 11.4943 | 2.2989 | 0.0000 |
| MESO | NRP1 | 5.7471 | 25.2874 | 4.5977 | 25.2874 | 1.1494 | 0.0000 |
| MESO | SCARB1 | 22.9885 | 3.4483 | 22.9885 | 3.4483 | 0.0000 | 0.0000 |
| MESO | TMPRSS2 | 6.8966 | 12.6437 | 6.8966 | 12.6437 | 0.0000 | 0.0000 |
| OV | ACE2 | 16.0622 | 59.5855 | 13.4715 | 58.0311 | 2.5907 | 1.5544 |
| OV | AXL | 25.7340 | 40.4145 | 22.1071 | 39.0328 | 3.6269 | 1.3817 |
| OV | NRP1 | 41.2781 | 12.0898 | 37.6511 | 12.0898 | 3.6269 | 0.0000 |
| OV | SCARB1 | 30.9154 | 33.3333 | 27.8066 | 33.3333 | 3.1088 | 0.0000 |
| OV | TMPRSS2 | 22.6252 | 39.2055 | 20.3800 | 38.3420 | 2.2453 | 0.8636 |
| PAAD | ACE2 | 4.8913 | 14.6739 | 4.3478 | 14.1304 | 0.5435 | 0.5435 |
| PAAD | AXL | 19.5652 | 8.1522 | 16.8478 | 8.1522 | 2.7174 | 0.0000 |
| PAAD | NRP1 | 6.5217 | 16.8478 | 5.9783 | 16.8478 | 0.5435 | 0.0000 |
| PAAD | SCARB1 | 9.7826 | 21.1957 | 9.7826 | 21.1957 | 0.0000 | 0.0000 |
| PAAD | TMPRSS2 | 1.6304 | 32.0652 | 1.6304 | 32.0652 | 0.0000 | 0.0000 |
| PCPG | ACE2 | 4.3210 | 27.7778 | 4.3210 | 27.7778 | 0.0000 | 0.0000 |
| PCPG | AXL | 7.4074 | 4.9383 | 7.4074 | 4.9383 | 0.0000 | 0.0000 |
| PCPG | NRP1 | 6.7901 | 0.6173 | 6.7901 | 0.6173 | 0.0000 | 0.0000 |
| PCPG | SCARB1 | 9.8765 | 1.8519 | 8.0247 | 1.8519 | 1.8519 | 0.0000 |
| PCPG | TMPRSS2 | 2.4691 | 21.6049 | 1.8519 | 21.6049 | 0.6173 | 0.0000 |
| PRAD | ACE2 | 5.0813 | 4.8780 | 3.6585 | 4.6748 | 1.4228 | 0.2033 |
| PRAD | AXL | 2.0325 | 6.9106 | 2.0325 | 6.0976 | 0.0000 | 0.8130 |
| PRAD | NRP1 | 3.2520 | 9.5528 | 3.0488 | 9.1463 | 0.2033 | 0.4065 |
| PRAD | SCARB1 | 4.6748 | 8.3333 | 3.8618 | 7.1138 | 0.8130 | 1.2195 |
| PRAD | TMPRSS2 | 3.8618 | 33.5366 | 3.6585 | 18.0894 | 0.2033 | 15.4472 |
| READ | ACE2 | 19.3939 | 16.9697 | 18.1818 | 16.9697 | 1.2121 | 0.0000 |
| READ | AXL | 22.4242 | 10.3030 | 22.4242 | 10.3030 | 0.0000 | 0.0000 |
| READ | NRP1 | 6.6667 | 16.3636 | 6.6667 | 16.3636 | 0.0000 | 0.0000 |
| READ | SCARB1 | 15.1515 | 13.9394 | 15.1515 | 13.9394 | 0.0000 | 0.0000 |
| READ | TMPRSS2 | 6.6667 | 41.8182 | 6.6667 | 41.8182 | 0.0000 | 0.0000 |
| SARC | ACE2 | 33.0739 | 24.9027 | 29.9611 | 24.5136 | 3.1128 | 0.3891 |
| SARC | AXL | 27.2374 | 25.6809 | 21.7899 | 25.6809 | 5.4475 | 0.0000 |
| SARC | NRP1 | 12.0623 | 41.6342 | 11.2840 | 41.6342 | 0.7782 | 0.0000 |
| SARC | SCARB1 | 19.0661 | 24.1245 | 16.7315 | 24.1245 | 2.3346 | 0.0000 |
| SARC | TMPRSS2 | 24.5136 | 23.3463 | 23.3463 | 23.3463 | 1.1673 | 0.0000 |
| SKCM | ACE2 | 15.8038 | 22.6158 | 14.7139 | 22.6158 | 1.0899 | 0.0000 |
| SKCM | AXL | 15.2589 | 19.6185 | 14.7139 | 19.6185 | 0.5450 | 0.0000 |
| SKCM | NRP1 | 4.0872 | 53.4060 | 3.5422 | 52.8610 | 0.5450 | 0.5450 |
| SKCM | SCARB1 | 9.5368 | 23.4332 | 8.9918 | 23.4332 | 0.5450 | 0.0000 |
| SKCM | TMPRSS2 | 19.0736 | 19.6185 | 18.8011 | 19.3460 | 0.2725 | 0.2725 |
| STAD | ACE2 | 15.4195 | 16.0998 | 14.2857 | 14.5125 | 1.1338 | 1.5873 |
| STAD | AXL | 20.8617 | 17.2336 | 18.8209 | 17.0068 | 2.0408 | 0.2268 |
| STAD | NRP1 | 26.7574 | 10.8844 | 25.8503 | 10.6576 | 0.9070 | 0.2268 |
| STAD | SCARB1 | 22.2222 | 14.2857 | 21.0884 | 14.0590 | 1.1338 | 0.2268 |
| STAD | TMPRSS2 | 6.5760 | 44.2177 | 6.1224 | 42.4036 | 0.4535 | 1.8141 |
| TGCT | ACE2 | 19.3333 | 18.0000 | 19.3333 | 16.6667 | 0.0000 | 1.3333 |
| TGCT | AXL | 18.6667 | 36.6667 | 18.6667 | 36.6667 | 0.0000 | 0.0000 |
| TGCT | NRP1 | 5.3333 | 52.0000 | 5.3333 | 51.3333 | 0.0000 | 0.6667 |
| TGCT | SCARB1 | 56.6667 | 1.3333 | 56.6667 | 1.3333 | 0.0000 | 0.0000 |
| TGCT | TMPRSS2 | 84.6667 | 2.0000 | 84.0000 | 2.0000 | 0.6667 | 0.0000 |
| THCA | ACE2 | 1.8036 | 0.6012 | 1.4028 | 0.6012 | 0.4008 | 0.0000 |
| THCA | AXL | 2.0040 | 0.2004 | 2.0040 | 0.2004 | 0.0000 | 0.0000 |
| THCA | NRP1 | 0.6012 | 1.2024 | 0.4008 | 1.2024 | 0.2004 | 0.0000 |
| THCA | SCARB1 | 3.0060 | 0.0000 | 3.0060 | 0.0000 | 0.0000 | 0.0000 |
| THCA | TMPRSS2 | 1.0020 | 1.6032 | 1.0020 | 1.6032 | 0.0000 | 0.0000 |
| THYM | ACE2 | 1.6260 | 6.5041 | 1.6260 | 6.5041 | 0.0000 | 0.0000 |
| THYM | AXL | 2.4390 | 2.4390 | 2.4390 | 2.4390 | 0.0000 | 0.0000 |
| THYM | NRP1 | 1.6260 | 1.6260 | 1.6260 | 0.8130 | 0.0000 | 0.8130 |
| THYM | SCARB1 | 3.2520 | 5.6911 | 3.2520 | 5.6911 | 0.0000 | 0.0000 |
| THYM | TMPRSS2 | 1.6260 | 8.9431 | 1.6260 | 8.1301 | 0.0000 | 0.8130 |
| UCEC | ACE2 | 13.9147 | 15.3989 | 13.9147 | 15.0278 | 0.0000 | 0.3711 |
| UCEC | AXL | 11.3173 | 10.7607 | 9.8330 | 10.5751 | 1.4842 | 0.1855 |
| UCEC | NRP1 | 23.3766 | 7.0501 | 22.4490 | 7.0501 | 0.9276 | 0.0000 |
| UCEC | SCARB1 | 11.1317 | 12.4304 | 9.2764 | 12.4304 | 1.8553 | 0.0000 |
| UCEC | TMPRSS2 | 9.4620 | 10.3896 | 7.9777 | 10.3896 | 1.4842 | 0.0000 |
| UCS | ACE2 | 35.7143 | 26.7857 | 35.7143 | 26.7857 | 0.0000 | 0.0000 |
| UCS | AXL | 51.7857 | 30.3571 | 42.8571 | 30.3571 | 8.9286 | 0.0000 |
| UCS | NRP1 | 48.2143 | 26.7857 | 44.6429 | 26.7857 | 3.5714 | 0.0000 |
| UCS | SCARB1 | 25.0000 | 32.1429 | 23.2143 | 32.1429 | 1.7857 | 0.0000 |
| UCS | TMPRSS2 | 35.7143 | 30.3571 | 33.9286 | 30.3571 | 1.7857 | 0.0000 |
| UVM | ACE2 | 12.5000 | 15.0000 | 12.5000 | 15.0000 | 0.0000 | 0.0000 |
| UVM | AXL | 2.5000 | 3.7500 | 2.5000 | 3.7500 | 0.0000 | 0.0000 |
| UVM | NRP1 | 0.0000 | 1.2500 | 0.0000 | 1.2500 | 0.0000 | 0.0000 |
| UVM | SCARB1 | 3.7500 | 2.5000 | 3.7500 | 2.5000 | 0.0000 | 0.0000 |
| UVM | TMPRSS2 | 20.0000 | 1.2500 | 18.7500 | 1.2500 | 1.2500 | 0.0000 |

Table S7. CnvAndExpressionTable

| cancertype | symbol | spm | fdr |
| --- | --- | --- | --- |
| ACC | ACE2 | 0.2911 | 0.0537 |
| ACC | AXL | -0.1919 | 0.2651 |
| ACC | NRP1 | 0.2189 | 0.1843 |
| ACC | SCARB1 | -0.0853 | 0.7059 |
| ACC | TMPRSS2 | 0.2120 | 0.2033 |
| BLCA | ACE2 | -0.0516 | 0.4436 |
| BLCA | AXL | -0.0492 | 0.4687 |
| BLCA | NRP1 | -0.0913 | 0.1318 |
| BLCA | SCARB1 | 0.2339 | 0.0000 |
| BLCA | TMPRSS2 | 0.3063 | 0.0000 |
| BRCA | ACE2 | 0.0782 | 0.0185 |
| BRCA | AXL | 0.0781 | 0.0187 |
| BRCA | NRP1 | -0.0187 | 0.6222 |
| BRCA | SCARB1 | 0.1513 | 0.0000 |
| BRCA | TMPRSS2 | 0.1871 | 0.0000 |
| CESC | ACE2 | 0.1349 | 0.0538 |
| CESC | AXL | 0.2568 | 0.0000 |
| CESC | NRP1 | 0.0034 | 0.9749 |
| CESC | SCARB1 | 0.1257 | 0.0758 |
| CESC | TMPRSS2 | 0.1083 | 0.1371 |
| CHOL | ACE2 | -0.1285 | 0.8618 |
| CHOL | AXL | 0.2877 | 0.4885 |
| CHOL | NRP1 | 0.2916 | 0.4780 |
| CHOL | SCARB1 | 0.1670 | 0.7875 |
| CHOL | TMPRSS2 | 0.4648 | 0.1104 |
| COAD | ACE2 | 0.1101 | 0.1582 |
| COAD | AXL | 0.1032 | 0.1944 |
| COAD | NRP1 | -0.0057 | 0.9666 |
| COAD | SCARB1 | 0.1958 | 0.0039 |
| COAD | TMPRSS2 | 0.4380 | 0.0000 |
| DLBC | ACE2 | 0.0950 | 1.0000 |
| DLBC | AXL | -0.1951 | 1.0000 |
| DLBC | NRP1 | -0.1372 | 1.0000 |
| DLBC | SCARB1 | 0.2304 | 1.0000 |
| DLBC | TMPRSS2 | 0.0959 | 1.0000 |
| ESCA | ACE2 | 0.1462 | 0.1077 |
| ESCA | AXL | 0.0801 | 0.4337 |
| ESCA | NRP1 | 0.0620 | 0.5637 |
| ESCA | SCARB1 | 0.2761 | 0.0007 |
| ESCA | TMPRSS2 | 0.0231 | 0.8498 |
| GBM | ACE2 | -0.0897 | 0.5759 |
| GBM | AXL | 0.0211 | 0.9371 |
| GBM | NRP1 | 0.2754 | 0.0077 |
| GBM | SCARB1 | 0.0549 | 0.7856 |
| GBM | TMPRSS2 | 0.1526 | 0.2302 |
| HNSC | ACE2 | 0.1782 | 0.0002 |
| HNSC | AXL | 0.0840 | 0.1091 |
| HNSC | NRP1 | 0.0163 | 0.8062 |
| HNSC | SCARB1 | 0.0960 | 0.0613 |
| HNSC | TMPRSS2 | 0.2102 | 0.0000 |
| KICH | ACE2 | 0.0558 | 0.9120 |
| KICH | AXL | 0.0379 | 0.9480 |
| KICH | NRP1 | 0.2317 | 0.3006 |
| KICH | SCARB1 | 0.4138 | 0.0164 |
| KICH | TMPRSS2 | 0.3526 | 0.0534 |
| KIRC | ACE2 | -0.0934 | 0.0963 |
| KIRC | AXL | 0.0726 | 0.2248 |
| KIRC | NRP1 | 0.1421 | 0.0060 |
| KIRC | SCARB1 | 0.0960 | 0.0849 |
| KIRC | TMPRSS2 | -0.0611 | 0.3286 |
| KIRP | ACE2 | 0.1507 | 0.0434 |
| KIRP | AXL | 0.0567 | 0.5552 |
| KIRP | NRP1 | 0.0632 | 0.4992 |
| KIRP | SCARB1 | 0.2739 | 0.0000 |
| KIRP | TMPRSS2 | 0.0962 | 0.2492 |
| LAML | ACE2 | 0.0399 | 0.9999 |
| LAML | AXL | -0.0543 | 0.9999 |
| LAML | NRP1 | -0.0661 | 0.9999 |
| LAML | SCARB1 | 0.0310 | 0.9999 |
| LAML | TMPRSS2 | 0.1013 | 0.9999 |
| LGG | ACE2 | -0.0008 | 0.9939 |
| LGG | AXL | 0.5099 | 0.0000 |
| LGG | NRP1 | -0.1478 | 0.0043 |
| LGG | SCARB1 | 0.3270 | 0.0000 |
| LGG | TMPRSS2 | -0.0307 | 0.6866 |
| LIHC | ACE2 | 0.0502 | 0.5244 |
| LIHC | AXL | 0.1411 | 0.0228 |
| LIHC | NRP1 | 0.1281 | 0.0426 |
| LIHC | SCARB1 | 0.2386 | 0.0000 |
| LIHC | TMPRSS2 | 0.2910 | 0.0000 |
| LUAD | ACE2 | 0.1016 | 0.0428 |
| LUAD | AXL | 0.0699 | 0.1842 |
| LUAD | NRP1 | 0.1106 | 0.0258 |
| LUAD | SCARB1 | 0.3728 | 0.0000 |
| LUAD | TMPRSS2 | 0.3524 | 0.0000 |
| LUSC | ACE2 | 0.1048 | 0.0346 |
| LUSC | AXL | 0.2473 | 0.0000 |
| LUSC | NRP1 | 0.0913 | 0.0694 |
| LUSC | SCARB1 | 0.3584 | 0.0000 |
| LUSC | TMPRSS2 | 0.2147 | 0.0000 |
| MESO | ACE2 | 0.0541 | 0.8527 |
| MESO | AXL | 0.1341 | 0.5166 |
| MESO | NRP1 | 0.0496 | 0.8675 |
| MESO | SCARB1 | 0.1279 | 0.5424 |
| MESO | TMPRSS2 | 0.1762 | 0.3388 |
| OV | ACE2 | 0.1404 | 0.0263 |
| OV | AXL | 0.1779 | 0.0041 |
| OV | NRP1 | 0.0215 | 0.7716 |
| OV | SCARB1 | 0.4630 | 0.0000 |
| OV | TMPRSS2 | 0.1214 | 0.0580 |
| PAAD | ACE2 | 0.1002 | 0.3826 |
| PAAD | AXL | 0.0805 | 0.5105 |
| PAAD | NRP1 | -0.1296 | 0.2248 |
| PAAD | SCARB1 | 0.3565 | 0.0000 |
| PAAD | TMPRSS2 | 0.2827 | 0.0015 |
| PCPG | ACE2 | 0.0987 | 0.5644 |
| PCPG | AXL | -0.1770 | 0.1634 |
| PCPG | NRP1 | -0.0941 | 0.5891 |
| PCPG | SCARB1 | 0.0788 | 0.6812 |
| PCPG | TMPRSS2 | 0.0061 | 0.9908 |
| PRAD | ACE2 | 0.0823 | 0.2434 |
| PRAD | AXL | 0.1007 | 0.1223 |
| PRAD | NRP1 | 0.0854 | 0.2179 |
| PRAD | SCARB1 | 0.1675 | 0.0030 |
| PRAD | TMPRSS2 | 0.4233 | 0.0000 |
| READ | ACE2 | 0.1920 | 0.2016 |
| READ | AXL | -0.0193 | 0.9557 |
| READ | NRP1 | 0.1898 | 0.2096 |
| READ | SCARB1 | 0.3113 | 0.0147 |
| READ | TMPRSS2 | 0.4635 | 0.0001 |
| SARC | ACE2 | 0.0537 | 0.5444 |
| SARC | AXL | 0.4049 | 0.0000 |
| SARC | NRP1 | 0.3946 | 0.0000 |
| SARC | SCARB1 | 0.1875 | 0.0086 |
| SARC | TMPRSS2 | -0.0599 | 0.4910 |
| SKCM | ACE2 | -0.1382 | 0.0216 |
| SKCM | AXL | 0.1872 | 0.0011 |
| SKCM | NRP1 | 0.0000 | 1.0000 |
| SKCM | SCARB1 | 0.2550 | 0.0000 |
| SKCM | TMPRSS2 | 0.1577 | 0.0075 |
| STAD | ACE2 | 0.0726 | 0.2460 |
| STAD | AXL | 0.1812 | 0.0008 |
| STAD | NRP1 | 0.0188 | 0.8100 |
| STAD | SCARB1 | 0.1546 | 0.0053 |
| STAD | TMPRSS2 | 0.2839 | 0.0000 |
| TGCT | ACE2 | 0.0094 | 0.9639 |
| TGCT | AXL | -0.2672 | 0.0069 |
| TGCT | NRP1 | 0.0456 | 0.7696 |
| TGCT | SCARB1 | 0.1209 | 0.3145 |
| TGCT | TMPRSS2 | 0.1691 | 0.1229 |
| THCA | ACE2 | 0.0133 | 0.9985 |
| THCA | AXL | -0.0805 | 0.6275 |
| THCA | NRP1 | 0.0610 | 0.7991 |
| THCA | SCARB1 | 0.1281 | 0.1820 |
| THCA | TMPRSS2 | -0.1862 | 0.0117 |
| THYM | ACE2 | -0.1297 | 0.6557 |
| THYM | AXL | 0.2457 | 0.1842 |
| THYM | NRP1 | 0.0192 | 0.9880 |
| THYM | SCARB1 | 0.1234 | 0.6848 |
| THYM | TMPRSS2 | 0.0764 | 0.8545 |
| UCEC | ACE2 | 0.1378 | 0.1731 |
| UCEC | AXL | 0.1820 | 0.0537 |
| UCEC | NRP1 | -0.0817 | 0.4796 |
| UCEC | SCARB1 | 0.2232 | 0.0132 |
| UCEC | TMPRSS2 | 0.1818 | 0.0540 |
| UCS | ACE2 | 0.0319 | 0.9219 |
| UCS | AXL | 0.3003 | 0.1009 |
| UCS | NRP1 | 0.2911 | 0.1146 |
| UCS | SCARB1 | 0.3214 | 0.0713 |
| UCS | TMPRSS2 | 0.0302 | 0.9255 |
| UVM | ACE2 | 0.2533 | 0.3419 |
| UVM | AXL | 0.0936 | 0.9160 |
| UVM | NRP1 | 0.0898 | 0.9298 |
| UVM | SCARB1 | 0.0721 | 0.9605 |
| UVM | TMPRSS2 | 0.0100 | 0.9991 |

Table S8. CnvAndSurvivalTable

| cancertype | symbol | sur_type | log_rank_p |
| --- | --- | --- | --- |
| ACC | ACE2 | OS | 0.8697 |
| ACC | ACE2 | PFS | 0.2835 |
| ACC | ACE2 | DSS | 0.9252 |
| ACC | ACE2 | DFI | 0.4949 |
| ACC | AXL | OS | 0.3976 |
| ACC | AXL | PFS | 0.2501 |
| ACC | AXL | DSS | 0.1906 |
| ACC | AXL | DFI | 0.5247 |
| ACC | NRP1 | OS | 0.0132 |
| ACC | NRP1 | PFS | 0.0380 |
| ACC | NRP1 | DSS | 0.0116 |
| ACC | NRP1 | DFI | 0.3609 |
| ACC | SCARB1 | OS | 0.1076 |
| ACC | SCARB1 | PFS | 0.0788 |
| ACC | SCARB1 | DSS | 0.2223 |
| ACC | SCARB1 | DFI | 0.0860 |
| ACC | TMPRSS2 | OS | 0.0928 |
| ACC | TMPRSS2 | PFS | 0.3083 |
| ACC | TMPRSS2 | DSS | 0.0683 |
| ACC | TMPRSS2 | DFI | 0.5089 |
| BLCA | ACE2 | OS | 0.2766 |
| BLCA | ACE2 | PFS | 0.3659 |
| BLCA | ACE2 | DSS | 0.1864 |
| BLCA | ACE2 | DFI | 0.9951 |
| BLCA | AXL | OS | 0.1656 |
| BLCA | AXL | PFS | 0.3802 |
| BLCA | AXL | DSS | 0.0706 |
| BLCA | AXL | DFI | 0.8574 |
| BLCA | NRP1 | OS | 0.0968 |
| BLCA | NRP1 | PFS | 0.1728 |
| BLCA | NRP1 | DSS | 0.2044 |
| BLCA | NRP1 | DFI | 0.4638 |
| BLCA | SCARB1 | OS | 0.4022 |
| BLCA | SCARB1 | PFS | 0.7235 |
| BLCA | SCARB1 | DSS | 0.1333 |
| BLCA | SCARB1 | DFI | 0.5969 |
| BLCA | TMPRSS2 | OS | 0.8741 |
| BLCA | TMPRSS2 | PFS | 0.4282 |
| BLCA | TMPRSS2 | DSS | 0.9557 |
| BLCA | TMPRSS2 | DFI | 0.9276 |
| BRCA | ACE2 | OS | 0.8251 |
| BRCA | ACE2 | PFS | 0.3805 |
| BRCA | ACE2 | DSS | 0.4568 |
| BRCA | ACE2 | DFI | 0.8915 |
| BRCA | AXL | OS | 0.1162 |
| BRCA | AXL | PFS | 0.4220 |
| BRCA | AXL | DSS | 0.0251 |
| BRCA | AXL | DFI | 0.4591 |
| BRCA | NRP1 | OS | 0.2027 |
| BRCA | NRP1 | PFS | 0.4913 |
| BRCA | NRP1 | DSS | 0.5393 |
| BRCA | NRP1 | DFI | 0.8658 |
| BRCA | SCARB1 | OS | 0.0571 |
| BRCA | SCARB1 | PFS | 0.0088 |
| BRCA | SCARB1 | DSS | 0.1361 |
| BRCA | SCARB1 | DFI | 0.0821 |
| BRCA | TMPRSS2 | OS | 0.4421 |
| BRCA | TMPRSS2 | PFS | 0.1241 |
| BRCA | TMPRSS2 | DSS | 0.0821 |
| BRCA | TMPRSS2 | DFI | 0.0029 |
| CESC | ACE2 | OS | 0.8555 |
| CESC | ACE2 | PFS | 0.7980 |
| CESC | ACE2 | DSS | 0.7047 |
| CESC | ACE2 | DFI | 0.9046 |
| CESC | AXL | OS | 0.6202 |
| CESC | AXL | PFS | 0.9003 |
| CESC | AXL | DSS | 0.7796 |
| CESC | AXL | DFI | 0.1945 |
| CESC | NRP1 | OS | 0.0472 |
| CESC | NRP1 | PFS | 0.0193 |
| CESC | NRP1 | DSS | 0.2631 |
| CESC | NRP1 | DFI | 0.0095 |
| CESC | SCARB1 | OS | 0.7571 |
| CESC | SCARB1 | PFS | 0.8701 |
| CESC | SCARB1 | DSS | 0.5674 |
| CESC | SCARB1 | DFI | 0.3030 |
| CESC | TMPRSS2 | OS | 0.1117 |
| CESC | TMPRSS2 | PFS | 0.0822 |
| CESC | TMPRSS2 | DSS | 0.3140 |
| CESC | TMPRSS2 | DFI | 0.2023 |
| CHOL | ACE2 | OS | 0.7562 |
| CHOL | ACE2 | PFS | 0.1784 |
| CHOL | ACE2 | DSS | 0.7530 |
| CHOL | ACE2 | DFI | 0.3498 |
| CHOL | AXL | OS | 0.5202 |
| CHOL | AXL | PFS | 0.6862 |
| CHOL | AXL | DSS | 0.4514 |
| CHOL | AXL | DFI | 0.6734 |
| CHOL | NRP1 | OS | 0.8265 |
| CHOL | NRP1 | PFS | 0.7532 |
| CHOL | NRP1 | DSS | 0.8009 |
| CHOL | NRP1 | DFI | 0.1078 |
| CHOL | SCARB1 | OS | 0.7525 |
| CHOL | SCARB1 | PFS | 0.9438 |
| CHOL | SCARB1 | DSS | 0.2902 |
| CHOL | SCARB1 | DFI | 0.5744 |
| CHOL | TMPRSS2 | OS | 0.7472 |
| CHOL | TMPRSS2 | PFS | 0.6175 |
| CHOL | TMPRSS2 | DSS | 0.8042 |
| CHOL | TMPRSS2 | DFI | 0.6621 |
| COAD | ACE2 | OS | 0.1851 |
| COAD | ACE2 | PFS | 0.2640 |
| COAD | ACE2 | DSS | 0.1324 |
| COAD | ACE2 | DFI | 0.0216 |
| COAD | AXL | OS | 0.2580 |
| COAD | AXL | PFS | 0.0211 |
| COAD | AXL | DSS | 0.2446 |
| COAD | AXL | DFI | 0.8023 |
| COAD | NRP1 | OS | 0.0228 |
| COAD | NRP1 | PFS | 0.0015 |
| COAD | NRP1 | DSS | 0.0216 |
| COAD | NRP1 | DFI | 0.0061 |
| COAD | SCARB1 | OS | 0.5955 |
| COAD | SCARB1 | PFS | 0.4149 |
| COAD | SCARB1 | DSS | 0.8335 |
| COAD | SCARB1 | DFI | 0.3118 |
| COAD | TMPRSS2 | OS | 0.9706 |
| COAD | TMPRSS2 | PFS | 0.4963 |
| COAD | TMPRSS2 | DSS | 0.3262 |
| COAD | TMPRSS2 | DFI | 0.1636 |
| DLBC | ACE2 | OS | 0.5262 |
| DLBC | ACE2 | PFS | 0.5276 |
| DLBC | ACE2 | DSS | 0.8642 |
| DLBC | ACE2 | DFI | 0.7530 |
| DLBC | AXL | OS | 0.2530 |
| DLBC | AXL | PFS | 0.8053 |
| DLBC | AXL | DSS | 0.4097 |
| DLBC | AXL | DFI | 0.1120 |
| DLBC | NRP1 | OS | 0.4721 |
| DLBC | NRP1 | PFS | 0.9088 |
| DLBC | NRP1 | DSS | 0.6094 |
| DLBC | NRP1 | DFI | 0.0155 |
| DLBC | SCARB1 | OS | 0.6525 |
| DLBC | SCARB1 | PFS | 0.6941 |
| DLBC | SCARB1 | DSS | 0.8667 |
| DLBC | SCARB1 | DFI | 0.0000 |
| DLBC | TMPRSS2 | OS | 0.6821 |
| DLBC | TMPRSS2 | PFS | 0.1705 |
| DLBC | TMPRSS2 | DSS | 0.7545 |
| DLBC | TMPRSS2 | DFI | 0.0000 |
| ESCA | ACE2 | OS | 0.1675 |
| ESCA | ACE2 | PFS | 0.0551 |
| ESCA | ACE2 | DSS | 0.1088 |
| ESCA | ACE2 | DFI | 0.6163 |
| ESCA | AXL | OS | 0.8171 |
| ESCA | AXL | PFS | 0.8037 |
| ESCA | AXL | DSS | 0.7673 |
| ESCA | AXL | DFI | 0.9234 |
| ESCA | NRP1 | OS | 0.5540 |
| ESCA | NRP1 | PFS | 0.2526 |
| ESCA | NRP1 | DSS | 0.1356 |
| ESCA | NRP1 | DFI | 0.2738 |
| ESCA | SCARB1 | OS | 0.3579 |
| ESCA | SCARB1 | PFS | 0.5884 |
| ESCA | SCARB1 | DSS | 0.1885 |
| ESCA | SCARB1 | DFI | 0.9162 |
| ESCA | TMPRSS2 | OS | 0.4398 |
| ESCA | TMPRSS2 | PFS | 0.2382 |
| ESCA | TMPRSS2 | DSS | 0.3752 |
| ESCA | TMPRSS2 | DFI | 0.5399 |
| GBM | ACE2 | OS | 0.1635 |
| GBM | ACE2 | PFS | 0.2381 |
| GBM | ACE2 | DSS | 0.1571 |
| GBM | AXL | OS | 0.0704 |
| GBM | AXL | PFS | 0.0926 |
| GBM | AXL | DSS | 0.0923 |
| GBM | NRP1 | OS | 0.0000 |
| GBM | NRP1 | PFS | 0.0000 |
| GBM | NRP1 | DSS | 0.0000 |
| GBM | SCARB1 | OS | 0.2494 |
| GBM | SCARB1 | PFS | 0.1437 |
| GBM | SCARB1 | DSS | 0.7316 |
| GBM | TMPRSS2 | OS | 0.6980 |
| GBM | TMPRSS2 | PFS | 0.4058 |
| GBM | TMPRSS2 | DSS | 0.5720 |
| HNSC | ACE2 | OS | 0.0550 |
| HNSC | ACE2 | PFS | 0.1071 |
| HNSC | ACE2 | DSS | 0.0363 |
| HNSC | ACE2 | DFI | 0.6843 |
| HNSC | AXL | OS | 0.3042 |
| HNSC | AXL | PFS | 0.0133 |
| HNSC | AXL | DSS | 0.1610 |
| HNSC | AXL | DFI | 0.6378 |
| HNSC | NRP1 | OS | 0.5313 |
| HNSC | NRP1 | PFS | 0.7129 |
| HNSC | NRP1 | DSS | 0.9552 |
| HNSC | NRP1 | DFI | 0.0987 |
| HNSC | SCARB1 | OS | 0.6921 |
| HNSC | SCARB1 | PFS | 0.5377 |
| HNSC | SCARB1 | DSS | 0.6697 |
| HNSC | SCARB1 | DFI | 0.3865 |
| HNSC | TMPRSS2 | OS | 0.8236 |
| HNSC | TMPRSS2 | PFS | 0.7715 |
| HNSC | TMPRSS2 | DSS | 0.8042 |
| HNSC | TMPRSS2 | DFI | 0.8602 |
| KICH | ACE2 | OS | 0.5802 |
| KICH | ACE2 | PFS | 0.2757 |
| KICH | ACE2 | DSS | 0.4256 |
| KICH | ACE2 | DFI | 0.3525 |
| KICH | AXL | OS | 0.0007 |
| KICH | AXL | PFS | 0.0060 |
| KICH | AXL | DSS | 0.0001 |
| KICH | AXL | DFI | 0.6213 |
| KICH | NRP1 | OS | 0.3138 |
| KICH | NRP1 | PFS | 0.7276 |
| KICH | NRP1 | DSS | 0.4215 |
| KICH | NRP1 | DFI | 0.1558 |
| KICH | SCARB1 | OS | 0.1955 |
| KICH | SCARB1 | PFS | 0.2441 |
| KICH | SCARB1 | DSS | 0.0446 |
| KICH | SCARB1 | DFI | 0.7574 |
| KICH | TMPRSS2 | OS | 0.1075 |
| KICH | TMPRSS2 | PFS | 0.1702 |
| KICH | TMPRSS2 | DSS | 0.2291 |
| KICH | TMPRSS2 | DFI | 0.2927 |
| KIRC | ACE2 | OS | 0.1317 |
| KIRC | ACE2 | PFS | 0.0409 |
| KIRC | ACE2 | DSS | 0.2252 |
| KIRC | ACE2 | DFI | 0.6761 |
| KIRC | AXL | OS | 0.0037 |
| KIRC | AXL | PFS | 0.0003 |
| KIRC | AXL | DSS | 0.0004 |
| KIRC | AXL | DFI | 0.0003 |
| KIRC | NRP1 | OS | 0.7893 |
| KIRC | NRP1 | PFS | 0.6079 |
| KIRC | NRP1 | DSS | 0.6804 |
| KIRC | NRP1 | DFI | 0.3702 |
| KIRC | SCARB1 | OS | 0.0630 |
| KIRC | SCARB1 | PFS | 0.0307 |
| KIRC | SCARB1 | DSS | 0.0417 |
| KIRC | SCARB1 | DFI | 0.6100 |
| KIRC | TMPRSS2 | OS | 0.6915 |
| KIRC | TMPRSS2 | PFS | 0.3258 |
| KIRC | TMPRSS2 | DSS | 0.6665 |
| KIRC | TMPRSS2 | DFI | 0.2615 |
| KIRP | ACE2 | OS | 0.0012 |
| KIRP | ACE2 | PFS | 0.0009 |
| KIRP | ACE2 | DSS | 0.0000 |
| KIRP | ACE2 | DFI | 0.0003 |
| KIRP | AXL | OS | 0.0855 |
| KIRP | AXL | PFS | 0.0009 |
| KIRP | AXL | DSS | 0.0115 |
| KIRP | AXL | DFI | 0.0009 |
| KIRP | NRP1 | OS | 0.0036 |
| KIRP | NRP1 | PFS | 0.0718 |
| KIRP | NRP1 | DSS | 0.0002 |
| KIRP | NRP1 | DFI | 0.0077 |
| KIRP | SCARB1 | OS | 0.9774 |
| KIRP | SCARB1 | PFS | 0.1991 |
| KIRP | SCARB1 | DSS | 0.6723 |
| KIRP | SCARB1 | DFI | 0.7335 |
| KIRP | TMPRSS2 | OS | 0.4894 |
| KIRP | TMPRSS2 | PFS | 0.9900 |
| KIRP | TMPRSS2 | DSS | 0.3263 |
| KIRP | TMPRSS2 | DFI | 0.9070 |
| LAML | ACE2 | OS | 0.1693 |
| LAML | AXL | OS | 0.4312 |
| LAML | NRP1 | OS | 0.0000 |
| LAML | SCARB1 | OS | 0.0130 |
| LAML | TMPRSS2 | OS | 0.0057 |
| LGG | ACE2 | OS | 0.6253 |
| LGG | ACE2 | PFS | 0.5902 |
| LGG | ACE2 | DSS | 0.6248 |
| LGG | ACE2 | DFI | 0.2415 |
| LGG | AXL | OS | 0.0000 |
| LGG | AXL | PFS | 0.0000 |
| LGG | AXL | DSS | 0.0000 |
| LGG | AXL | DFI | 0.0029 |
| LGG | NRP1 | OS | 0.0000 |
| LGG | NRP1 | PFS | 0.0000 |
| LGG | NRP1 | DSS | 0.0000 |
| LGG | NRP1 | DFI | 0.0000 |
| LGG | SCARB1 | OS | 0.5990 |
| LGG | SCARB1 | PFS | 0.4989 |
| LGG | SCARB1 | DSS | 0.7675 |
| LGG | SCARB1 | DFI | 0.1949 |
| LGG | TMPRSS2 | OS | 0.4275 |
| LGG | TMPRSS2 | PFS | 0.4487 |
| LGG | TMPRSS2 | DSS | 0.6422 |
| LGG | TMPRSS2 | DFI | 0.5080 |
| LIHC | ACE2 | OS | 0.9985 |
| LIHC | ACE2 | PFS | 0.7655 |
| LIHC | ACE2 | DSS | 0.5960 |
| LIHC | ACE2 | DFI | 0.4378 |
| LIHC | AXL | OS | 0.3514 |
| LIHC | AXL | PFS | 0.9874 |
| LIHC | AXL | DSS | 0.5111 |
| LIHC | AXL | DFI | 0.7218 |
| LIHC | NRP1 | OS | 0.0630 |
| LIHC | NRP1 | PFS | 0.1035 |
| LIHC | NRP1 | DSS | 0.0143 |
| LIHC | NRP1 | DFI | 0.1597 |
| LIHC | SCARB1 | OS | 0.0493 |
| LIHC | SCARB1 | PFS | 0.1763 |
| LIHC | SCARB1 | DSS | 0.3848 |
| LIHC | SCARB1 | DFI | 0.6104 |
| LIHC | TMPRSS2 | OS | 0.0289 |
| LIHC | TMPRSS2 | PFS | 0.4935 |
| LIHC | TMPRSS2 | DSS | 0.0716 |
| LIHC | TMPRSS2 | DFI | 0.9217 |
| LUAD | ACE2 | OS | 0.0612 |
| LUAD | ACE2 | PFS | 0.0091 |
| LUAD | ACE2 | DSS | 0.0052 |
| LUAD | ACE2 | DFI | 0.0283 |
| LUAD | AXL | OS | 0.0196 |
| LUAD | AXL | PFS | 0.0054 |
| LUAD | AXL | DSS | 0.0664 |
| LUAD | AXL | DFI | 0.1241 |
| LUAD | NRP1 | OS | 0.1083 |
| LUAD | NRP1 | PFS | 0.3888 |
| LUAD | NRP1 | DSS | 0.1297 |
| LUAD | NRP1 | DFI | 0.2059 |
| LUAD | SCARB1 | OS | 0.2476 |
| LUAD | SCARB1 | PFS | 0.7631 |
| LUAD | SCARB1 | DSS | 0.6966 |
| LUAD | SCARB1 | DFI | 0.8852 |
| LUAD | TMPRSS2 | OS | 0.6410 |
| LUAD | TMPRSS2 | PFS | 0.9063 |
| LUAD | TMPRSS2 | DSS | 0.8512 |
| LUAD | TMPRSS2 | DFI | 0.9134 |
| LUSC | ACE2 | OS | 0.6386 |
| LUSC | ACE2 | PFS | 0.4481 |
| LUSC | ACE2 | DSS | 0.8487 |
| LUSC | ACE2 | DFI | 0.1617 |
| LUSC | AXL | OS | 0.2509 |
| LUSC | AXL | PFS | 0.2808 |
| LUSC | AXL | DSS | 0.3919 |
| LUSC | AXL | DFI | 0.5808 |
| LUSC | NRP1 | OS | 0.8369 |
| LUSC | NRP1 | PFS | 0.9541 |
| LUSC | NRP1 | DSS | 0.8098 |
| LUSC | NRP1 | DFI | 0.8709 |
| LUSC | SCARB1 | OS | 0.5573 |
| LUSC | SCARB1 | PFS | 0.3275 |
| LUSC | SCARB1 | DSS | 0.2560 |
| LUSC | SCARB1 | DFI | 0.0479 |
| LUSC | TMPRSS2 | OS | 0.0545 |
| LUSC | TMPRSS2 | PFS | 0.0349 |
| LUSC | TMPRSS2 | DSS | 0.4543 |
| LUSC | TMPRSS2 | DFI | 0.0423 |
| MESO | ACE2 | OS | 0.4089 |
| MESO | ACE2 | PFS | 0.8145 |
| MESO | ACE2 | DSS | 0.6162 |
| MESO | AXL | OS | 0.5405 |
| MESO | AXL | PFS | 0.2193 |
| MESO | AXL | DSS | 0.5977 |
| MESO | NRP1 | OS | 0.0053 |
| MESO | NRP1 | PFS | 0.0773 |
| MESO | NRP1 | DSS | 0.0017 |
| MESO | NRP1 | DFI | 0.1546 |
| MESO | SCARB1 | OS | 0.1094 |
| MESO | SCARB1 | PFS | 0.0113 |
| MESO | SCARB1 | DSS | 0.1008 |
| MESO | SCARB1 | DFI | 0.8900 |
| MESO | TMPRSS2 | OS | 0.0382 |
| MESO | TMPRSS2 | PFS | 0.3078 |
| MESO | TMPRSS2 | DSS | 0.0436 |
| OV | ACE2 | OS | 0.7730 |
| OV | ACE2 | PFS | 0.8643 |
| OV | ACE2 | DSS | 0.9202 |
| OV | ACE2 | DFI | 0.9941 |
| OV | AXL | OS | 0.0648 |
| OV | AXL | PFS | 0.7519 |
| OV | AXL | DSS | 0.0674 |
| OV | AXL | DFI | 0.7265 |
| OV | NRP1 | OS | 0.5474 |
| OV | NRP1 | PFS | 0.7521 |
| OV | NRP1 | DSS | 0.6700 |
| OV | NRP1 | DFI | 0.7475 |
| OV | SCARB1 | OS | 0.9002 |
| OV | SCARB1 | PFS | 0.2768 |
| OV | SCARB1 | DSS | 0.8763 |
| OV | SCARB1 | DFI | 0.7472 |
| OV | TMPRSS2 | OS | 0.3084 |
| OV | TMPRSS2 | PFS | 0.6617 |
| OV | TMPRSS2 | DSS | 0.1916 |
| OV | TMPRSS2 | DFI | 0.7327 |
| PAAD | ACE2 | OS | 0.0250 |
| PAAD | ACE2 | PFS | 0.0182 |
| PAAD | ACE2 | DSS | 0.0060 |
| PAAD | ACE2 | DFI | 0.4041 |
| PAAD | AXL | OS | 0.0984 |
| PAAD | AXL | PFS | 0.2589 |
| PAAD | AXL | DSS | 0.0331 |
| PAAD | AXL | DFI | 0.9839 |
| PAAD | NRP1 | OS | 0.9731 |
| PAAD | NRP1 | PFS | 0.5971 |
| PAAD | NRP1 | DSS | 0.4820 |
| PAAD | NRP1 | DFI | 0.0360 |
| PAAD | SCARB1 | OS | 0.6204 |
| PAAD | SCARB1 | PFS | 0.1660 |
| PAAD | SCARB1 | DSS | 0.3849 |
| PAAD | SCARB1 | DFI | 0.3934 |
| PAAD | TMPRSS2 | OS | 0.7013 |
| PAAD | TMPRSS2 | PFS | 0.5922 |
| PAAD | TMPRSS2 | DSS | 0.9661 |
| PAAD | TMPRSS2 | DFI | 0.2027 |
| PCPG | ACE2 | OS | 0.7659 |
| PCPG | ACE2 | PFS | 0.5198 |
| PCPG | ACE2 | DSS | 0.9252 |
| PCPG | ACE2 | DFI | 0.8400 |
| PCPG | AXL | OS | 0.3577 |
| PCPG | AXL | PFS | 0.2896 |
| PCPG | AXL | DSS | 0.1343 |
| PCPG | AXL | DFI | 0.6905 |
| PCPG | NRP1 | OS | 0.5312 |
| PCPG | NRP1 | PFS | 0.2259 |
| PCPG | NRP1 | DSS | 0.6285 |
| PCPG | NRP1 | DFI | 0.5962 |
| PCPG | SCARB1 | OS | 0.7235 |
| PCPG | SCARB1 | PFS | 0.6871 |
| PCPG | SCARB1 | DSS | 0.8215 |
| PCPG | SCARB1 | DFI | 0.7738 |
| PCPG | TMPRSS2 | OS | 0.0101 |
| PCPG | TMPRSS2 | PFS | 0.0916 |
| PCPG | TMPRSS2 | DSS | 0.0007 |
| PCPG | TMPRSS2 | DFI | 0.5209 |
| PRAD | ACE2 | OS | 0.0169 |
| PRAD | ACE2 | PFS | 0.0850 |
| PRAD | ACE2 | DSS | 0.0002 |
| PRAD | ACE2 | DFI | 0.4230 |
| PRAD | AXL | OS | 0.6675 |
| PRAD | AXL | PFS | 0.0263 |
| PRAD | AXL | DSS | 0.3147 |
| PRAD | AXL | DFI | 0.0916 |
| PRAD | NRP1 | OS | 0.8769 |
| PRAD | NRP1 | PFS | 0.3926 |
| PRAD | NRP1 | DSS | 0.8595 |
| PRAD | NRP1 | DFI | 0.0446 |
| PRAD | SCARB1 | OS | 0.8457 |
| PRAD | SCARB1 | PFS | 0.7756 |
| PRAD | SCARB1 | DSS | 0.7697 |
| PRAD | SCARB1 | DFI | 0.9823 |
| PRAD | TMPRSS2 | OS | 0.6092 |
| PRAD | TMPRSS2 | PFS | 0.7464 |
| PRAD | TMPRSS2 | DSS | 0.4851 |
| PRAD | TMPRSS2 | DFI | 0.6961 |
| READ | ACE2 | OS | 0.5243 |
| READ | ACE2 | PFS | 0.4616 |
| READ | ACE2 | DSS | 0.7472 |
| READ | ACE2 | DFI | 0.9269 |
| READ | AXL | OS | 0.4690 |
| READ | AXL | PFS | 0.9874 |
| READ | AXL | DSS | 0.7891 |
| READ | AXL | DFI | 0.2493 |
| READ | NRP1 | OS | 0.1017 |
| READ | NRP1 | PFS | 0.8038 |
| READ | NRP1 | DSS | 0.1211 |
| READ | NRP1 | DFI | 0.5424 |
| READ | SCARB1 | OS | 0.5087 |
| READ | SCARB1 | PFS | 0.6301 |
| READ | SCARB1 | DSS | 0.6160 |
| READ | SCARB1 | DFI | 0.4336 |
| READ | TMPRSS2 | OS | 0.9326 |
| READ | TMPRSS2 | PFS | 0.7267 |
| READ | TMPRSS2 | DSS | 0.9409 |
| READ | TMPRSS2 | DFI | 0.0913 |
| SARC | ACE2 | OS | 0.2996 |
| SARC | ACE2 | PFS | 0.2151 |
| SARC | ACE2 | DSS | 0.0897 |
| SARC | ACE2 | DFI | 0.9812 |
| SARC | AXL | OS | 0.5428 |
| SARC | AXL | PFS | 0.1123 |
| SARC | AXL | DSS | 0.7495 |
| SARC | AXL | DFI | 0.6526 |
| SARC | NRP1 | OS | 0.5804 |
| SARC | NRP1 | PFS | 0.5203 |
| SARC | NRP1 | DSS | 0.5712 |
| SARC | NRP1 | DFI | 0.6145 |
| SARC | SCARB1 | OS | 0.5084 |
| SARC | SCARB1 | PFS | 0.5999 |
| SARC | SCARB1 | DSS | 0.6317 |
| SARC | SCARB1 | DFI | 0.3204 |
| SARC | TMPRSS2 | OS | 0.0229 |
| SARC | TMPRSS2 | PFS | 0.3257 |
| SARC | TMPRSS2 | DSS | 0.0201 |
| SARC | TMPRSS2 | DFI | 0.4696 |
| SKCM | ACE2 | OS | 0.3267 |
| SKCM | ACE2 | PFS | 0.1591 |
| SKCM | ACE2 | DSS | 0.3348 |
| SKCM | AXL | OS | 0.5684 |
| SKCM | AXL | PFS | 0.8500 |
| SKCM | AXL | DSS | 0.7086 |
| SKCM | NRP1 | OS | 0.3983 |
| SKCM | NRP1 | PFS | 0.4543 |
| SKCM | NRP1 | DSS | 0.5054 |
| SKCM | SCARB1 | OS | 0.1245 |
| SKCM | SCARB1 | PFS | 0.1814 |
| SKCM | SCARB1 | DSS | 0.2566 |
| SKCM | TMPRSS2 | OS | 0.5249 |
| SKCM | TMPRSS2 | PFS | 0.9706 |
| SKCM | TMPRSS2 | DSS | 0.6059 |
| STAD | ACE2 | OS | 0.6612 |
| STAD | ACE2 | PFS | 0.6200 |
| STAD | ACE2 | DSS | 0.8282 |
| STAD | ACE2 | DFI | 0.0372 |
| STAD | AXL | OS | 0.8369 |
| STAD | AXL | PFS | 0.5186 |
| STAD | AXL | DSS | 0.5133 |
| STAD | AXL | DFI | 0.8164 |
| STAD | NRP1 | OS | 0.4448 |
| STAD | NRP1 | PFS | 0.5868 |
| STAD | NRP1 | DSS | 0.2763 |
| STAD | NRP1 | DFI | 0.1676 |
| STAD | SCARB1 | OS | 0.5329 |
| STAD | SCARB1 | PFS | 0.8183 |
| STAD | SCARB1 | DSS | 0.6911 |
| STAD | SCARB1 | DFI | 0.0911 |
| STAD | TMPRSS2 | OS | 0.6638 |
| STAD | TMPRSS2 | PFS | 0.5228 |
| STAD | TMPRSS2 | DSS | 0.3377 |
| STAD | TMPRSS2 | DFI | 0.9177 |
| TGCT | ACE2 | OS | 0.3851 |
| TGCT | ACE2 | PFS | 0.3406 |
| TGCT | ACE2 | DSS | 0.4491 |
| TGCT | ACE2 | DFI | 0.1290 |
| TGCT | AXL | OS | 0.6997 |
| TGCT | AXL | PFS | 0.8093 |
| TGCT | AXL | DSS | 0.6738 |
| TGCT | AXL | DFI | 0.8667 |
| TGCT | NRP1 | OS | 0.7447 |
| TGCT | NRP1 | PFS | 0.6013 |
| TGCT | NRP1 | DSS | 0.8307 |
| TGCT | NRP1 | DFI | 0.8295 |
| TGCT | SCARB1 | OS | 0.9365 |
| TGCT | SCARB1 | PFS | 0.5957 |
| TGCT | SCARB1 | DSS | 0.6475 |
| TGCT | SCARB1 | DFI | 0.6513 |
| TGCT | TMPRSS2 | OS | 0.0734 |
| TGCT | TMPRSS2 | PFS | 0.0695 |
| TGCT | TMPRSS2 | DSS | 0.0093 |
| TGCT | TMPRSS2 | DFI | 0.0425 |
| THCA | ACE2 | OS | 0.2403 |
| THCA | ACE2 | PFS | 0.4338 |
| THCA | ACE2 | DSS | 0.0201 |
| THCA | ACE2 | DFI | 0.7326 |
| THCA | AXL | OS | 0.0441 |
| THCA | AXL | PFS | 0.1314 |
| THCA | AXL | DSS | 0.0018 |
| THCA | AXL | DFI | 0.0105 |
| THCA | NRP1 | OS | 0.8756 |
| THCA | NRP1 | PFS | 0.1096 |
| THCA | NRP1 | DSS | 0.9478 |
| THCA | NRP1 | DFI | 0.0055 |
| THCA | SCARB1 | OS | 0.4961 |
| THCA | SCARB1 | PFS | 0.5854 |
| THCA | SCARB1 | DSS | 0.6570 |
| THCA | SCARB1 | DFI | 0.3633 |
| THCA | TMPRSS2 | OS | 0.8423 |
| THCA | TMPRSS2 | PFS | 0.6298 |
| THCA | TMPRSS2 | DSS | 0.9243 |
| THCA | TMPRSS2 | DFI | 0.5123 |
| THYM | ACE2 | OS | 0.1572 |
| THYM | ACE2 | PFS | 0.0227 |
| THYM | ACE2 | DSS | 0.2847 |
| THYM | AXL | OS | 0.8839 |
| THYM | AXL | PFS | 0.2927 |
| THYM | AXL | DSS | 0.9436 |
| THYM | NRP1 | OS | 0.9084 |
| THYM | NRP1 | PFS | 0.6904 |
| THYM | NRP1 | DSS | 0.9683 |
| THYM | SCARB1 | OS | 0.2520 |
| THYM | SCARB1 | PFS | 0.3176 |
| THYM | SCARB1 | DSS | 0.0275 |
| THYM | TMPRSS2 | OS | 0.8346 |
| THYM | TMPRSS2 | PFS | 0.9520 |
| THYM | TMPRSS2 | DSS | 0.8820 |
| UCEC | ACE2 | OS | 0.0001 |
| UCEC | ACE2 | PFS | 0.0000 |
| UCEC | ACE2 | DSS | 0.0000 |
| UCEC | ACE2 | DFI | 0.0070 |
| UCEC | AXL | OS | 0.0000 |
| UCEC | AXL | PFS | 0.0000 |
| UCEC | AXL | DSS | 0.0000 |
| UCEC | AXL | DFI | 0.0150 |
| UCEC | NRP1 | OS | 0.4844 |
| UCEC | NRP1 | PFS | 0.1782 |
| UCEC | NRP1 | DSS | 0.5927 |
| UCEC | NRP1 | DFI | 0.6149 |
| UCEC | SCARB1 | OS | 0.0110 |
| UCEC | SCARB1 | PFS | 0.0860 |
| UCEC | SCARB1 | DSS | 0.0007 |
| UCEC | SCARB1 | DFI | 0.6450 |
| UCEC | TMPRSS2 | OS | 0.0045 |
| UCEC | TMPRSS2 | PFS | 0.0397 |
| UCEC | TMPRSS2 | DSS | 0.0191 |
| UCEC | TMPRSS2 | DFI | 0.7636 |
| UCS | ACE2 | OS | 0.2161 |
| UCS | ACE2 | PFS | 0.0905 |
| UCS | ACE2 | DSS | 0.3127 |
| UCS | ACE2 | DFI | 0.6703 |
| UCS | AXL | OS | 0.6752 |
| UCS | AXL | PFS | 0.5687 |
| UCS | AXL | DSS | 0.8105 |
| UCS | AXL | DFI | 0.4669 |
| UCS | NRP1 | OS | 0.5027 |
| UCS | NRP1 | PFS | 0.8555 |
| UCS | NRP1 | DSS | 0.4172 |
| UCS | NRP1 | DFI | 0.9589 |
| UCS | SCARB1 | OS | 0.4790 |
| UCS | SCARB1 | PFS | 0.5854 |
| UCS | SCARB1 | DSS | 0.4323 |
| UCS | SCARB1 | DFI | 0.2768 |
| UCS | TMPRSS2 | OS | 0.7467 |
| UCS | TMPRSS2 | PFS | 0.6580 |
| UCS | TMPRSS2 | DSS | 0.7375 |
| UCS | TMPRSS2 | DFI | 0.9193 |
| UVM | ACE2 | OS | 0.2884 |
| UVM | ACE2 | PFS | 0.1524 |
| UVM | ACE2 | DSS | 0.3037 |
| UVM | AXL | OS | 0.5002 |
| UVM | AXL | PFS | 0.2128 |
| UVM | AXL | DSS | 0.5526 |
| UVM | SCARB1 | OS | 0.7802 |
| UVM | SCARB1 | PFS | 0.9790 |
| UVM | SCARB1 | DSS | 0.7535 |
| UVM | TMPRSS2 | OS | 0.2571 |
| UVM | TMPRSS2 | PFS | 0.2553 |
| UVM | TMPRSS2 | DSS | 0.2566 |

Table S9. ExpressionAndMethylationTable

| cancertype | symbol | spm | fdr |
| --- | --- | --- | --- |
| ACC | ACE2 | -0.1737 | 0.1257 |
| ACC | AXL | -0.2230 | 0.0484 |
| ACC | NRP1 | -0.5320 | 0.0000 |
| ACC | SCARB1 | -0.6723 | 0.0000 |
| ACC | TMPRSS2 | -0.1369 | 0.2291 |
| BLCA | ACE2 | -0.1977 | 0.0001 |
| BLCA | AXL | -0.1353 | 0.0062 |
| BLCA | NRP1 | -0.1653 | 0.0008 |
| BLCA | SCARB1 | -0.2396 | 0.0000 |
| BLCA | TMPRSS2 | -0.4907 | 0.0000 |
| BRCA | ACE2 | -0.1274 | 0.0009 |
| BRCA | AXL | -0.1814 | 0.0000 |
| BRCA | NRP1 | -0.1388 | 0.0003 |
| BRCA | SCARB1 | -0.4213 | 0.0000 |
| BRCA | TMPRSS2 | -0.4527 | 0.0000 |
| CESC | ACE2 | -0.0179 | 0.7557 |
| CESC | AXL | -0.2068 | 0.0003 |
| CESC | NRP1 | -0.4789 | 0.0000 |
| CESC | SCARB1 | -0.3216 | 0.0000 |
| CESC | TMPRSS2 | -0.3893 | 0.0000 |
| CHOL | ACE2 | -0.2366 | 0.1644 |
| CHOL | AXL | -0.5637 | 0.0004 |
| CHOL | NRP1 | -0.3390 | 0.0437 |
| CHOL | SCARB1 | -0.4270 | 0.0099 |
| CHOL | TMPRSS2 | -0.4834 | 0.0031 |
| COAD | ACE2 | -0.6351 | 0.0000 |
| COAD | AXL | -0.1892 | 0.0018 |
| COAD | NRP1 | -0.3864 | 0.0000 |
| COAD | SCARB1 | -0.2786 | 0.0000 |
| COAD | TMPRSS2 | -0.5070 | 0.0000 |
| DLBC | ACE2 | -0.2385 | 0.1026 |
| DLBC | AXL | -0.3786 | 0.0083 |
| DLBC | NRP1 | -0.2914 | 0.0449 |
| DLBC | SCARB1 | -0.4048 | 0.0046 |
| DLBC | TMPRSS2 | -0.1955 | 0.1830 |
| ESCA | ACE2 | -0.0502 | 0.4985 |
| ESCA | AXL | -0.1382 | 0.0614 |
| ESCA | NRP1 | -0.3762 | 0.0000 |
| ESCA | SCARB1 | -0.4035 | 0.0000 |
| ESCA | TMPRSS2 | -0.6344 | 0.0000 |
| GBM | ACE2 | -0.1273 | 0.3735 |
| GBM | AXL | -0.0927 | 0.5167 |
| GBM | NRP1 | -0.3980 | 0.0041 |
| GBM | SCARB1 | -0.4201 | 0.0023 |
| GBM | TMPRSS2 | -0.3515 | 0.0114 |
| HNSC | ACE2 | -0.1483 | 0.0007 |
| HNSC | AXL | -0.1089 | 0.0130 |
| HNSC | NRP1 | -0.3071 | 0.0000 |
| HNSC | SCARB1 | -0.4033 | 0.0000 |
| HNSC | TMPRSS2 | -0.3477 | 0.0000 |
| KICH | ACE2 | -0.1239 | 0.3216 |
| KICH | AXL | -0.2606 | 0.0348 |
| KICH | NRP1 | -0.5090 | 0.0000 |
| KICH | SCARB1 | -0.3882 | 0.0014 |
| KICH | TMPRSS2 | -0.5877 | 0.0000 |
| KIRC | ACE2 | -0.2098 | 0.0002 |
| KIRC | AXL | 0.1064 | 0.0581 |
| KIRC | NRP1 | -0.4812 | 0.0000 |
| KIRC | SCARB1 | -0.5561 | 0.0000 |
| KIRC | TMPRSS2 | -0.1168 | 0.0373 |
| KIRP | ACE2 | -0.3926 | 0.0000 |
| KIRP | AXL | -0.2085 | 0.0005 |
| KIRP | NRP1 | -0.3444 | 0.0000 |
| KIRP | SCARB1 | -0.3792 | 0.0000 |
| KIRP | TMPRSS2 | -0.3914 | 0.0000 |
| LAML | ACE2 | -0.0957 | 0.2144 |
| LAML | AXL | -0.2272 | 0.0030 |
| LAML | NRP1 | -0.2824 | 0.0002 |
| LAML | SCARB1 | -0.1762 | 0.0217 |
| LAML | TMPRSS2 | -0.1128 | 0.1430 |
| LGG | ACE2 | -0.0154 | 0.7265 |
| LGG | AXL | -0.2021 | 0.0000 |
| LGG | NRP1 | -0.2548 | 0.0000 |
| LGG | SCARB1 | -0.3483 | 0.0000 |
| LGG | TMPRSS2 | -0.1418 | 0.0012 |
| LIHC | ACE2 | 0.1426 | 0.0059 |
| LIHC | AXL | -0.0841 | 0.1056 |
| LIHC | NRP1 | -0.4039 | 0.0000 |
| LIHC | SCARB1 | -0.4346 | 0.0000 |
| LIHC | TMPRSS2 | -0.2719 | 0.0000 |
| LUAD | ACE2 | -0.4073 | 0.0000 |
| LUAD | AXL | -0.0037 | 0.9365 |
| LUAD | NRP1 | -0.3266 | 0.0000 |
| LUAD | SCARB1 | -0.4298 | 0.0000 |
| LUAD | TMPRSS2 | -0.5259 | 0.0000 |
| LUSC | ACE2 | -0.2219 | 0.0000 |
| LUSC | AXL | -0.2661 | 0.0000 |
| LUSC | NRP1 | -0.4307 | 0.0000 |
| LUSC | SCARB1 | -0.2758 | 0.0000 |
| LUSC | TMPRSS2 | -0.3051 | 0.0000 |
| MESO | ACE2 | -0.1603 | 0.1381 |
| MESO | AXL | -0.3564 | 0.0008 |
| MESO | NRP1 | -0.6817 | 0.0000 |
| MESO | SCARB1 | -0.4588 | 0.0000 |
| MESO | TMPRSS2 | -0.1032 | 0.3416 |
| OV | ACE2 | -0.4667 | 0.2125 |
| OV | AXL | -0.9333 | 0.0007 |
| OV | NRP1 | -0.6667 | 0.0589 |
| OV | SCARB1 | -0.7000 | 0.0433 |
| OV | TMPRSS2 | -0.3500 | 0.3586 |
| PAAD | ACE2 | -0.0762 | 0.3117 |
| PAAD | AXL | -0.3389 | 0.0000 |
| PAAD | NRP1 | -0.4506 | 0.0000 |
| PAAD | SCARB1 | -0.2572 | 0.0006 |
| PAAD | TMPRSS2 | -0.5616 | 0.0000 |
| PCPG | ACE2 | -0.1282 | 0.0873 |
| PCPG | AXL | -0.1897 | 0.0111 |
| PCPG | NRP1 | -0.2475 | 0.0009 |
| PCPG | SCARB1 | -0.4031 | 0.0000 |
| PCPG | TMPRSS2 | -0.0362 | 0.6304 |
| PRAD | ACE2 | -0.2231 | 0.0000 |
| PRAD | AXL | -0.3269 | 0.0000 |
| PRAD | NRP1 | -0.3148 | 0.0000 |
| PRAD | SCARB1 | -0.2449 | 0.0000 |
| PRAD | TMPRSS2 | -0.5124 | 0.0000 |
| READ | ACE2 | -0.5815 | 0.0000 |
| READ | AXL | -0.1918 | 0.0671 |
| READ | NRP1 | -0.4160 | 0.0000 |
| READ | SCARB1 | -0.2292 | 0.0282 |
| READ | TMPRSS2 | -0.3606 | 0.0004 |
| SARC | ACE2 | -0.0265 | 0.6711 |
| SARC | AXL | -0.4493 | 0.0000 |
| SARC | NRP1 | -0.3094 | 0.0000 |
| SARC | SCARB1 | -0.3664 | 0.0000 |
| SARC | TMPRSS2 | -0.1689 | 0.0064 |
| SKCM | ACE2 | -0.1233 | 0.0075 |
| SKCM | AXL | -0.3356 | 0.0000 |
| SKCM | NRP1 | -0.3942 | 0.0000 |
| SKCM | SCARB1 | -0.3462 | 0.0000 |
| SKCM | TMPRSS2 | -0.2395 | 0.0000 |
| STAD | ACE2 | -0.2512 | 0.0000 |
| STAD | AXL | -0.0882 | 0.0895 |
| STAD | NRP1 | -0.1013 | 0.0510 |
| STAD | SCARB1 | -0.3563 | 0.0000 |
| STAD | TMPRSS2 | -0.6827 | 0.0000 |
| TGCT | ACE2 | 0.2081 | 0.0106 |
| TGCT | AXL | -0.3166 | 0.0001 |
| TGCT | NRP1 | -0.2216 | 0.0065 |
| TGCT | SCARB1 | -0.2591 | 0.0014 |
| TGCT | TMPRSS2 | -0.4847 | 0.0000 |
| THCA | ACE2 | -0.0565 | 0.2051 |
| THCA | AXL | -0.5052 | 0.0000 |
| THCA | NRP1 | -0.2745 | 0.0000 |
| THCA | SCARB1 | -0.4068 | 0.0000 |
| THCA | TMPRSS2 | -0.1850 | 0.0000 |
| THYM | ACE2 | -0.3696 | 0.0000 |
| THYM | AXL | -0.5746 | 0.0000 |
| THYM | NRP1 | -0.6402 | 0.0000 |
| THYM | SCARB1 | -0.4195 | 0.0000 |
| THYM | TMPRSS2 | -0.4021 | 0.0000 |
| UCEC | ACE2 | -0.0820 | 0.2844 |
| UCEC | AXL | -0.1024 | 0.1812 |
| UCEC | NRP1 | -0.2372 | 0.0018 |
| UCEC | SCARB1 | -0.1582 | 0.0384 |
| UCEC | TMPRSS2 | -0.4949 | 0.0000 |
| UCS | ACE2 | -0.1811 | 0.1776 |
| UCS | AXL | -0.4080 | 0.0018 |
| UCS | NRP1 | -0.5523 | 0.0000 |
| UCS | SCARB1 | -0.3286 | 0.0129 |
| UCS | TMPRSS2 | -0.2181 | 0.1031 |
| UVM | ACE2 | -0.1318 | 0.2437 |
| UVM | AXL | -0.4267 | 0.0001 |
| UVM | NRP1 | -0.4780 | 0.0000 |
| UVM | SCARB1 | -0.4667 | 0.0000 |
| UVM | TMPRSS2 | -0.1045 | 0.3561 |

Table S10. MethylationAndSurvivalTable

| cancertype | symbol | sur_type | log_rank_p | cox_p | HR | higher_risk_of_death |
| --- | --- | --- | --- | --- | --- | --- |
| ACC | ACE2 | OS | 0.0413 | 0.0467 | 2.1954 | Higher meth. |
| ACC | AXL | OS | 0.0224 | 0.0269 | 2.4325 | Higher meth. |
| ACC | NRP1 | OS | 0.0001 | 0.0003 | 0.2007 | Lower meth. |
| ACC | SCARB1 | OS | 0.3747 | 0.3771 | 0.7163 | Lower meth. |
| ACC | TMPRSS2 | OS | 0.0698 | 0.0753 | 1.9828 | Higher meth. |
| BLCA | ACE2 | OS | 0.1481 | 0.1489 | 0.8050 | Lower meth. |
| BLCA | AXL | OS | 0.9059 | 0.9066 | 0.9826 | Lower meth. |
| BLCA | NRP1 | OS | 0.2933 | 0.2936 | 0.8549 | Lower meth. |
| BLCA | SCARB1 | OS | 0.2455 | 0.2457 | 0.8404 | Lower meth. |
| BLCA | TMPRSS2 | OS | 0.4727 | 0.4727 | 1.1133 | Higher meth. |
| BRCA | ACE2 | OS | 0.8188 | 0.8189 | 1.0490 | Higher meth. |
| BRCA | AXL | OS | 0.7832 | 0.7829 | 0.9438 | Lower meth. |
| BRCA | NRP1 | OS | 0.2278 | 0.2292 | 0.7760 | Lower meth. |
| BRCA | SCARB1 | OS | 0.1276 | 0.1292 | 0.7261 | Lower meth. |
| BRCA | TMPRSS2 | OS | 0.8718 | 0.8716 | 0.9669 | Lower meth. |
| CESC | ACE2 | OS | 0.9505 | 0.9513 | 1.0145 | Higher meth. |
| CESC | AXL | OS | 0.2305 | 0.2321 | 0.7535 | Lower meth. |
| CESC | NRP1 | OS | 0.3728 | 0.3742 | 0.8094 | Lower meth. |
| CESC | SCARB1 | OS | 0.8523 | 0.8523 | 0.9570 | Lower meth. |
| CESC | TMPRSS2 | OS | 0.4208 | 0.4218 | 0.8270 | Lower meth. |
| CHOL | ACE2 | OS | 0.5467 | 0.5481 | 1.3390 | Higher meth. |
| CHOL | AXL | OS | 0.6091 | 0.6100 | 0.7786 | Lower meth. |
| CHOL | NRP1 | OS | 0.1304 | 0.1394 | 0.4715 | Lower meth. |
| CHOL | SCARB1 | OS | 0.6199 | 0.6206 | 0.7894 | Lower meth. |
| CHOL | TMPRSS2 | OS | 0.3689 | 0.3724 | 0.6527 | Lower meth. |
| COAD | ACE2 | OS | 0.8532 | 0.8532 | 1.0457 | Higher meth. |
| COAD | AXL | OS | 0.0713 | 0.0735 | 0.6460 | Lower meth. |
| COAD | NRP1 | OS | 0.3951 | 0.3956 | 0.8143 | Lower meth. |
| COAD | SCARB1 | OS | 0.8198 | 0.8198 | 1.0566 | Higher meth. |
| COAD | TMPRSS2 | OS | 0.3365 | 0.3374 | 1.2632 | Higher meth. |
| DLBC | ACE2 | OS | 0.4137 | 0.4203 | 0.5537 | Lower meth. |
| DLBC | AXL | OS | 0.3434 | 0.3553 | 0.4611 | Lower meth. |
| DLBC | NRP1 | OS | 0.9728 | 0.9728 | 0.9756 | Lower meth. |
| DLBC | SCARB1 | OS | 0.3886 | 0.3961 | 1.8607 | Higher meth. |
| DLBC | TMPRSS2 | OS | 0.3560 | 0.3646 | 0.5146 | Lower meth. |
| ESCA | ACE2 | OS | 0.0223 | 0.0238 | 0.5882 | Lower meth. |
| ESCA | AXL | OS | 0.8922 | 0.8917 | 1.0317 | Higher meth. |
| ESCA | NRP1 | OS | 0.7247 | 0.7248 | 0.9217 | Lower meth. |
| ESCA | SCARB1 | OS | 0.2772 | 0.2781 | 1.2822 | Higher meth. |
| ESCA | TMPRSS2 | OS | 0.4413 | 0.4420 | 1.1995 | Higher meth. |
| GBM | ACE2 | OS | 0.3686 | 0.3682 | 1.2088 | Higher meth. |
| GBM | AXL | OS | 0.7335 | 0.7343 | 1.0732 | Higher meth. |
| GBM | NRP1 | OS | 0.1455 | 0.1464 | 0.7381 | Lower meth. |
| GBM | SCARB1 | OS | 0.0537 | 0.0550 | 0.6625 | Lower meth. |
| GBM | TMPRSS2 | OS | 0.0067 | 0.0075 | 0.5701 | Lower meth. |
| HNSC | ACE2 | OS | 0.6804 | 0.6804 | 0.9460 | Lower meth. |
| HNSC | AXL | OS | 0.4568 | 0.4569 | 1.1055 | Higher meth. |
| HNSC | NRP1 | OS | 0.0433 | 0.0439 | 0.7612 | Lower meth. |
| HNSC | SCARB1 | OS | 0.5839 | 0.5842 | 0.9291 | Lower meth. |
| HNSC | TMPRSS2 | OS | 0.1575 | 0.1581 | 1.2098 | Higher meth. |
| KICH | ACE2 | OS | 0.3843 | 0.3915 | 1.8344 | Higher meth. |
| KICH | AXL | OS | 0.7167 | 0.7173 | 1.2757 | Higher meth. |
| KICH | NRP1 | OS | 0.8845 | 0.8846 | 1.1023 | Higher meth. |
| KICH | SCARB1 | OS | 0.9562 | 0.9562 | 1.0375 | Higher meth. |
| KICH | TMPRSS2 | OS | 0.4673 | 0.4716 | 1.6213 | Higher meth. |
| KIRC | ACE2 | OS | 0.3496 | 0.3500 | 1.2020 | Higher meth. |
| KIRC | AXL | OS | 0.0617 | 0.0633 | 1.4424 | Higher meth. |
| KIRC | NRP1 | OS | 0.0080 | 0.0087 | 1.6874 | Higher meth. |
| KIRC | SCARB1 | OS | 0.0068 | 0.0075 | 1.7083 | Higher meth. |
| KIRC | TMPRSS2 | OS | 0.0108 | 0.0117 | 1.6564 | Higher meth. |
| KIRP | ACE2 | OS | 0.8350 | 0.8350 | 1.0683 | Higher meth. |
| KIRP | AXL | OS | 0.1015 | 0.1055 | 1.6982 | Higher meth. |
| KIRP | NRP1 | OS | 0.1390 | 0.1427 | 0.6228 | Lower meth. |
| KIRP | SCARB1 | OS | 0.0545 | 0.0584 | 0.5381 | Lower meth. |
| KIRP | TMPRSS2 | OS | 0.0268 | 0.0302 | 2.0530 | Higher meth. |
| LAML | ACE2 | OS | 0.1942 | 0.1886 | 0.7839 | Lower meth. |
| LAML | AXL | OS | 0.6865 | 0.6787 | 1.0794 | Higher meth. |
| LAML | NRP1 | OS | 0.1351 | 0.1382 | 1.3150 | Higher meth. |
| LAML | SCARB1 | OS | 0.7335 | 0.7265 | 0.9376 | Lower meth. |
| LAML | TMPRSS2 | OS | 0.7377 | 0.7423 | 0.9410 | Lower meth. |
| LGG | ACE2 | OS | 0.1904 | 0.1914 | 1.2653 | Higher meth. |
| LGG | AXL | OS | 0.1030 | 0.1042 | 0.7461 | Lower meth. |
| LGG | NRP1 | OS | 0.0000 | 0.0000 | 0.4121 | Lower meth. |
| LGG | SCARB1 | OS | 0.0010 | 0.0012 | 1.8193 | Higher meth. |
| LGG | TMPRSS2 | OS | 0.9191 | 0.9197 | 1.0183 | Higher meth. |
| LIHC | ACE2 | OS | 0.0001 | 0.0002 | 0.5075 | Lower meth. |
| LIHC | AXL | OS | 0.0375 | 0.0385 | 0.6953 | Lower meth. |
| LIHC | NRP1 | OS | 0.0432 | 0.0443 | 0.7023 | Lower meth. |
| LIHC | SCARB1 | OS | 0.4671 | 0.4665 | 0.8796 | Lower meth. |
| LIHC | TMPRSS2 | OS | 0.3882 | 0.3891 | 0.8595 | Lower meth. |
| LUAD | ACE2 | OS | 0.5906 | 0.5905 | 1.0888 | Higher meth. |
| LUAD | AXL | OS | 0.7890 | 0.7890 | 0.9584 | Lower meth. |
| LUAD | NRP1 | OS | 0.7310 | 0.7313 | 0.9472 | Lower meth. |
| LUAD | SCARB1 | OS | 0.1946 | 0.1953 | 0.8126 | Lower meth. |
| LUAD | TMPRSS2 | OS | 0.0002 | 0.0002 | 1.8106 | Higher meth. |
| LUSC | ACE2 | OS | 0.7750 | 0.7757 | 1.0471 | Higher meth. |
| LUSC | AXL | OS | 0.8683 | 0.8679 | 0.9735 | Lower meth. |
| LUSC | NRP1 | OS | 0.8310 | 0.8314 | 0.9660 | Lower meth. |
| LUSC | SCARB1 | OS | 0.2823 | 0.2830 | 0.8406 | Lower meth. |
| LUSC | TMPRSS2 | OS | 0.3245 | 0.3254 | 0.8529 | Lower meth. |
| MESO | ACE2 | OS | 0.7150 | 0.7164 | 1.0904 | Higher meth. |
| MESO | AXL | OS | 0.0007 | 0.0009 | 0.4411 | Lower meth. |
| MESO | NRP1 | OS | 0.0058 | 0.0066 | 0.5134 | Lower meth. |
| MESO | SCARB1 | OS | 0.5629 | 0.5622 | 1.1466 | Higher meth. |
| MESO | TMPRSS2 | OS | 0.3931 | 0.3933 | 0.8164 | Lower meth. |
| OV | ACE2 | OS | 0.9794 | 0.9794 | 1.0244 | Higher meth. |
| OV | AXL | OS | 0.8972 | 0.8973 | 0.8860 | Lower meth. |
| OV | NRP1 | OS | 0.2896 | 0.3133 | 0.3180 | Lower meth. |
| OV | SCARB1 | OS | 0.5799 | 0.5837 | 1.6593 | Higher meth. |
| OV | TMPRSS2 | OS | 0.7247 | 0.7258 | 0.7236 | Lower meth. |
| PAAD | ACE2 | OS | 0.7418 | 0.7425 | 0.9358 | Lower meth. |
| PAAD | AXL | OS | 0.0038 | 0.0043 | 0.5566 | Lower meth. |
| PAAD | NRP1 | OS | 0.7943 | 0.7949 | 1.0539 | Higher meth. |
| PAAD | SCARB1 | OS | 0.8807 | 0.8787 | 0.9696 | Lower meth. |
| PAAD | TMPRSS2 | OS | 0.2754 | 0.2760 | 0.8010 | Lower meth. |
| PCPG | ACE2 | OS | 0.8599 | 0.8600 | 1.1553 | Higher meth. |
| PCPG | AXL | OS | 0.1060 | 0.1444 | 4.9765 | Higher meth. |
| PCPG | NRP1 | OS | 0.1108 | 0.1491 | 4.8845 | Higher meth. |
| PCPG | SCARB1 | OS | 0.5507 | 0.5548 | 1.6847 | Higher meth. |
| PCPG | TMPRSS2 | OS | 0.5282 | 0.5329 | 0.5764 | Lower meth. |
| PRAD | ACE2 | OS | 0.5500 | 0.5525 | 1.4678 | Higher meth. |
| PRAD | AXL | OS | 0.3737 | 0.3800 | 0.5659 | Lower meth. |
| PRAD | NRP1 | OS | 0.9757 | 0.9757 | 0.9797 | Lower meth. |
| PRAD | SCARB1 | OS | 0.0099 | 0.0218 | 0.1547 | Lower meth. |
| PRAD | TMPRSS2 | OS | 0.8815 | 0.8815 | 0.9094 | Lower meth. |
| READ | ACE2 | OS | 0.3324 | 0.3368 | 1.6088 | Higher meth. |
| READ | AXL | OS | 0.1631 | 0.1723 | 0.4827 | Lower meth. |
| READ | NRP1 | OS | 0.6576 | 0.6582 | 0.8053 | Lower meth. |
| READ | SCARB1 | OS | 0.9874 | 0.9874 | 1.0080 | Higher meth. |
| READ | TMPRSS2 | OS | 0.0579 | 0.0696 | 2.8265 | Higher meth. |
| SARC | ACE2 | OS | 0.1536 | 0.1555 | 0.7478 | Lower meth. |
| SARC | AXL | OS | 0.2224 | 0.2236 | 0.7813 | Lower meth. |
| SARC | NRP1 | OS | 0.6153 | 0.6151 | 1.1065 | Higher meth. |
| SARC | SCARB1 | OS | 0.8599 | 0.8604 | 1.0361 | Higher meth. |
| SARC | TMPRSS2 | OS | 0.5404 | 0.5411 | 0.8841 | Lower meth. |
| SKCM | ACE2 | OS | 0.0891 | 0.0898 | 0.7952 | Lower meth. |
| SKCM | AXL | OS | 0.7486 | 0.7494 | 0.9578 | Lower meth. |
| SKCM | NRP1 | OS | 0.7509 | 0.7511 | 0.9581 | Lower meth. |
| SKCM | SCARB1 | OS | 0.0001 | 0.0001 | 0.5868 | Lower meth. |
| SKCM | TMPRSS2 | OS | 0.3173 | 0.3175 | 0.8736 | Lower meth. |
| STAD | ACE2 | OS | 0.3730 | 0.3733 | 1.1577 | Higher meth. |
| STAD | AXL | OS | 0.4787 | 0.4791 | 0.8905 | Lower meth. |
| STAD | NRP1 | OS | 0.9339 | 0.9352 | 0.9868 | Lower meth. |
| STAD | SCARB1 | OS | 0.8999 | 0.9010 | 1.0207 | Higher meth. |
| STAD | TMPRSS2 | OS | 0.2357 | 0.2372 | 1.2139 | Higher meth. |
| TGCT | ACE2 | OS | 0.9338 | 0.9339 | 0.9203 | Lower meth. |
| TGCT | AXL | OS | 0.3309 | 0.3537 | 2.9185 | Higher meth. |
| TGCT | NRP1 | OS | 0.4502 | 0.4630 | 0.4232 | Lower meth. |
| TGCT | SCARB1 | OS | 0.0426 | 0.9987 | 6.11E+08 | Higher meth. |
| TGCT | TMPRSS2 | OS | 0.5346 | 0.5439 | 2.1035 | Higher meth. |
| THCA | ACE2 | OS | 0.3716 | 0.3758 | 0.6325 | Lower meth. |
| THCA | AXL | OS | 0.4917 | 0.4937 | 1.4129 | Higher meth. |
| THCA | NRP1 | OS | 0.4887 | 0.4909 | 0.7065 | Lower meth. |
| THCA | SCARB1 | OS | 0.9269 | 0.9269 | 1.0474 | Higher meth. |
| THCA | TMPRSS2 | OS | 0.9770 | 0.9770 | 1.0145 | Higher meth. |
| THYM | ACE2 | OS | 0.6668 | 0.6678 | 0.7489 | Lower meth. |
| THYM | AXL | OS | 0.0010 | 0.9980 | 0.0000 | Lower meth. |
| THYM | NRP1 | OS | 0.6767 | 0.6776 | 0.7478 | Lower meth. |
| THYM | SCARB1 | OS | 0.0023 | 0.0159 | 0.0751 | Lower meth. |
| THYM | TMPRSS2 | OS | 0.0446 | 0.0643 | 4.7287 | Higher meth. |
| UCEC | ACE2 | OS | 0.1531 | 0.1550 | 0.7115 | Lower meth. |
| UCEC | AXL | OS | 0.6256 | 0.6258 | 1.1212 | Higher meth. |
| UCEC | NRP1 | OS | 0.0510 | 0.0530 | 1.6012 | Higher meth. |
| UCEC | SCARB1 | OS | 0.6969 | 0.6967 | 0.9125 | Lower meth. |
| UCEC | TMPRSS2 | OS | 0.9228 | 0.9225 | 0.9774 | Lower meth. |
| UCS | ACE2 | OS | 0.8868 | 0.8840 | 1.0515 | Higher meth. |
| UCS | AXL | OS | 0.6988 | 0.6993 | 0.8768 | Lower meth. |
| UCS | NRP1 | OS | 0.4456 | 0.4472 | 0.7716 | Lower meth. |
| UCS | SCARB1 | OS | 0.3454 | 0.3478 | 0.7229 | Lower meth. |
| UCS | TMPRSS2 | OS | 0.9502 | 0.9501 | 0.9789 | Lower meth. |
| UVM | ACE2 | OS | 0.0057 | 0.0099 | 3.6918 | Higher meth. |
| UVM | AXL | OS | 0.6165 | 0.6172 | 1.2498 | Higher meth. |
| UVM | NRP1 | OS | 0.1427 | 0.1489 | 0.5373 | Lower meth. |
| UVM | SCARB1 | OS | 0.0117 | 0.0163 | 0.3352 | Lower meth. |
| UVM | TMPRSS2 | OS | 0.0077 | 0.0124 | 0.2818 | Lower meth. |

**Note**: The abnormal data for calculated HR in OS and DSS types of the gene AXL in TGCT (Table S3) and HR calculated in OS type of the gene SCARB1 in TGCT (Table S10) are caused by small number of events and very shorter follow-up **[1]**. But it is not a calculation error, as also noted personally by GSCA database developer on Feb 2, 2023. Looking at COX *p* value close to 1 also reveals that this is not significant in the Cox Proportional-Hazards model.

**Reference:**

**[1]** Liu J, Lichtenberg T, Hoadley KA, Poisson LM, Lazar AJ, Cherniack AD, et al. An integrated TCGA pan-cancer clinical data resource to drive high-quality survival outcome analytics. Cell (2018) 173:400–416.e11. doi: 10.1016/j.cell.2018.02.052
